# Supplementary material for: Gilteritinib overcomes lorlatinib resistance in ALK-rearranged cancer
Source: Nat Commun. 2021 Feb 24;12:1261. doi: 10.1038/s41467-021-21396-w (PMC7904790; doi:10.1038/s41467-021-21396-w)
Supplement: Supplementary file 1 — Supplementary Information [file 41467_2021_21396_MOESM1_ESM.pdf]

A

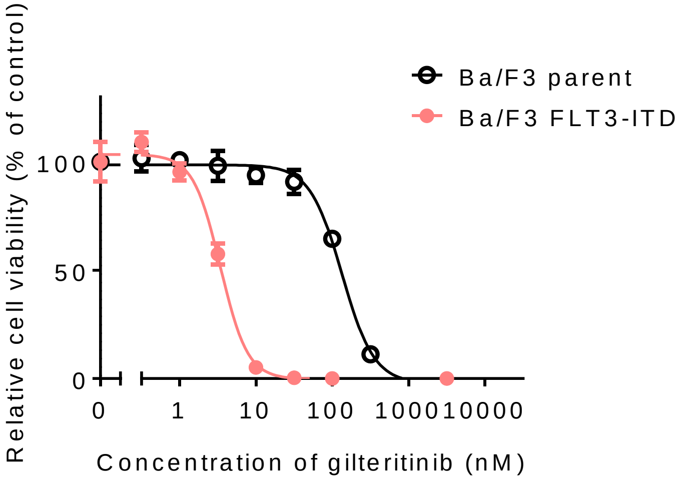

B

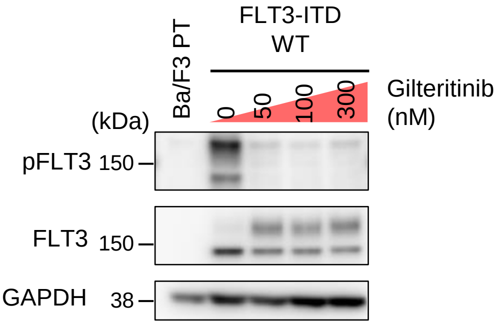

Supplementary Figure 1. Efficacy of gilteritinib in FLT3-ITD expressing Ba/F3 cells.

(A) Parental Ba/F3 cells and FLT3-ITD expressing Ba/F3 cells were treated with gilteritinib for 72 h and the cell viability was assessed using the CellTiter-Glo assay. N=3 independent samples examined over 3 independent experiments and representative experiment data are presented as mean values +/- SD. (B) Phospho-FLT3 in FLT3-ITD expressing Ba/F3 cells were evaluated by western blotting. Cells were treated with the indicated concentrations of gilteritinib for 3 h (n=2).

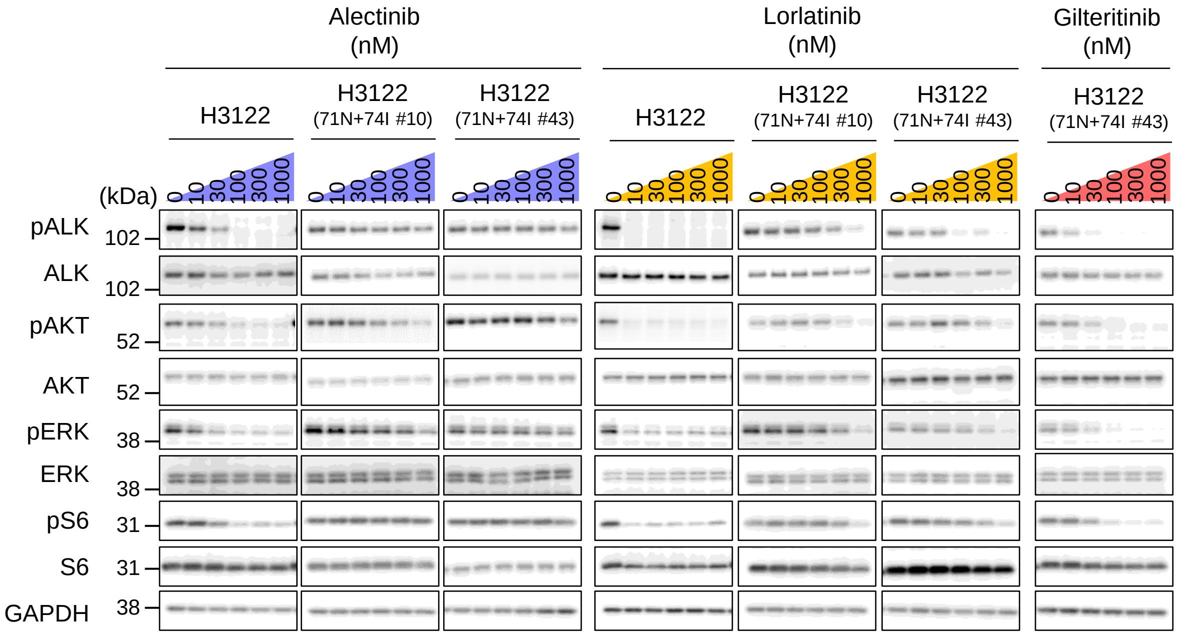

Supplementary Figure 2. Efficacy of gilteritinib in ALK I1171N + F1174I mutant expressed H3122 cells.

Suppression of phospho-ALK and its downstream signals in parental or ALK I1171N + F1174I carrying H3122 cells were evaluated by western blotting. Cells were treated with the indicated concentrations of gilteritinib for 6 h. pALK, ALK and GAPDH were identical to Fig. 1C (n=2).

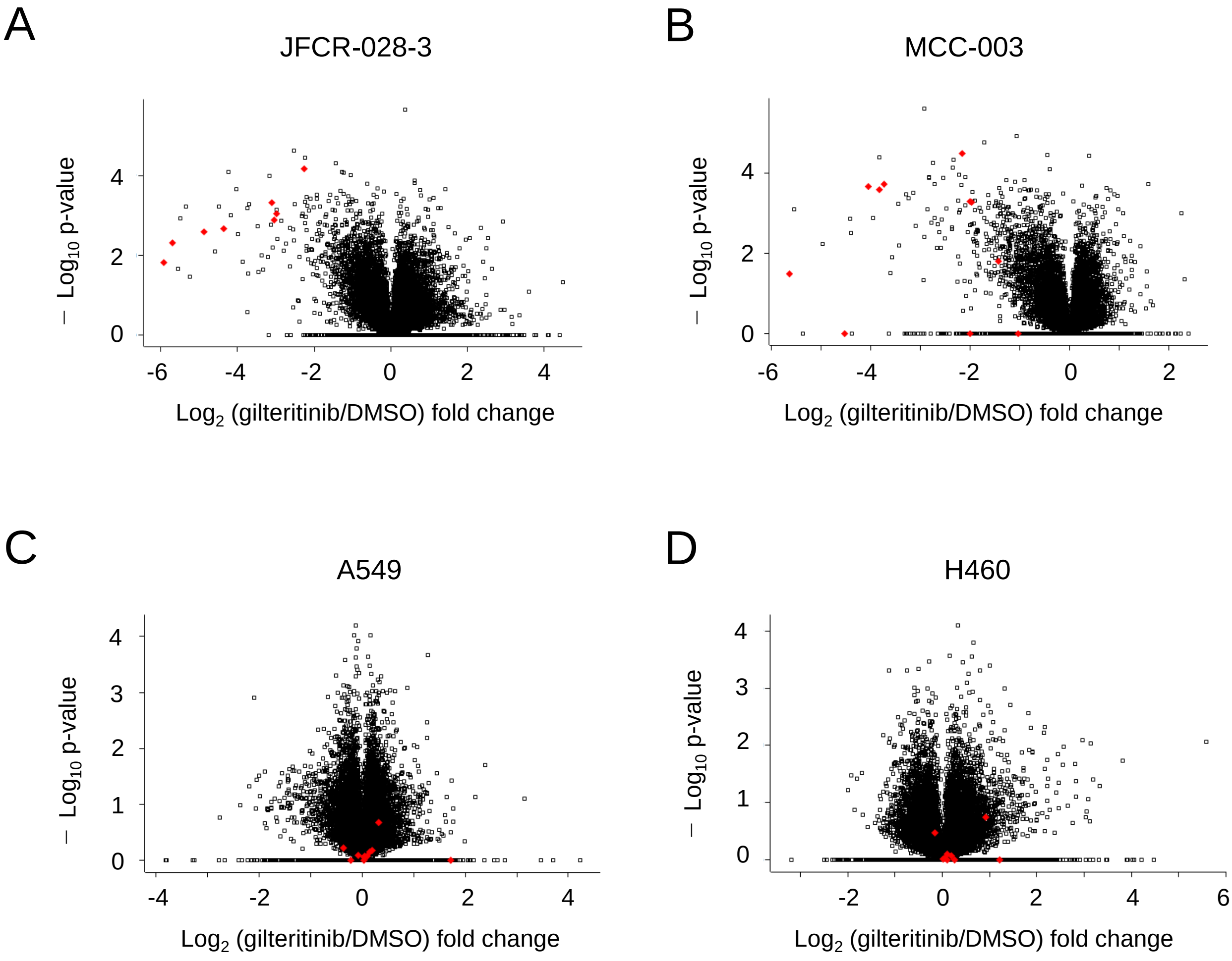

Supplementary Figure 3. Volcano plot of phosphoproteomic data.

Volcano plots are depicted with the fold change of each phosphosite and the p value was calculated by performing a student's t-test. The averages of the phosphoproteomic expression data of each gilteritinib treated group (A: JFCR-028-3, n = 3, B: MCC-003, n= 3, C: A549, n=3, D: H460, n=2) were compared with the averages of the data for each DMSO treated group. Red diamonds indicate phosphosites of ALK.

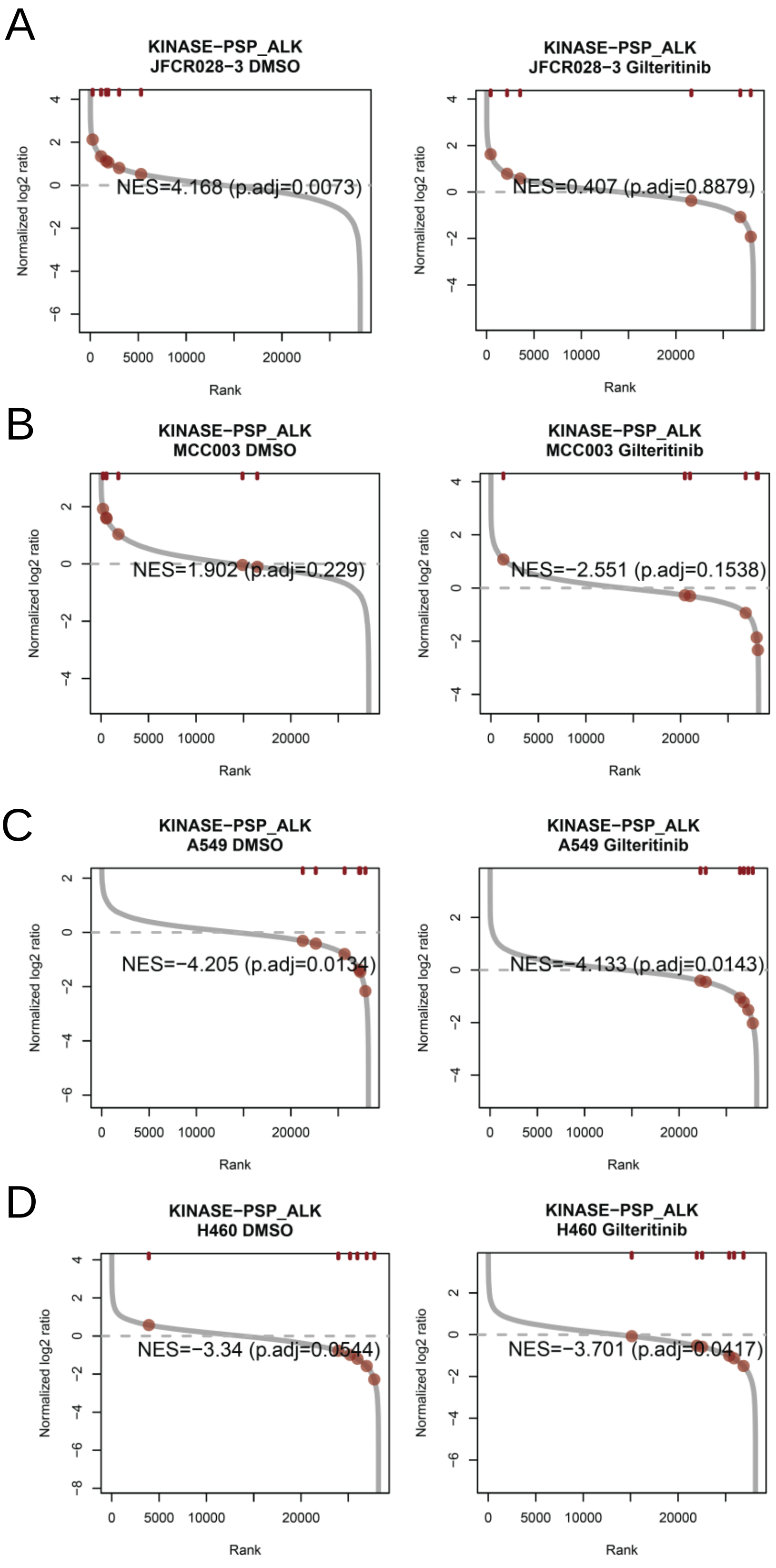

Supplementary Figure 4. Rank plot of ALK substrates.

The averages of the phosphoproteomic expression data of each gilteritinib or DMSO treated group (A: JFCR-028-3, n = 3, B: MCC-003, n= 3, C: A549, n=3, D: H460, n=2) were analyzed by PTM Signature Enrichment Analysis (PTM-SEA) using PTMsigDB v1.9.0. . Red circles indicate substrates of ALK.

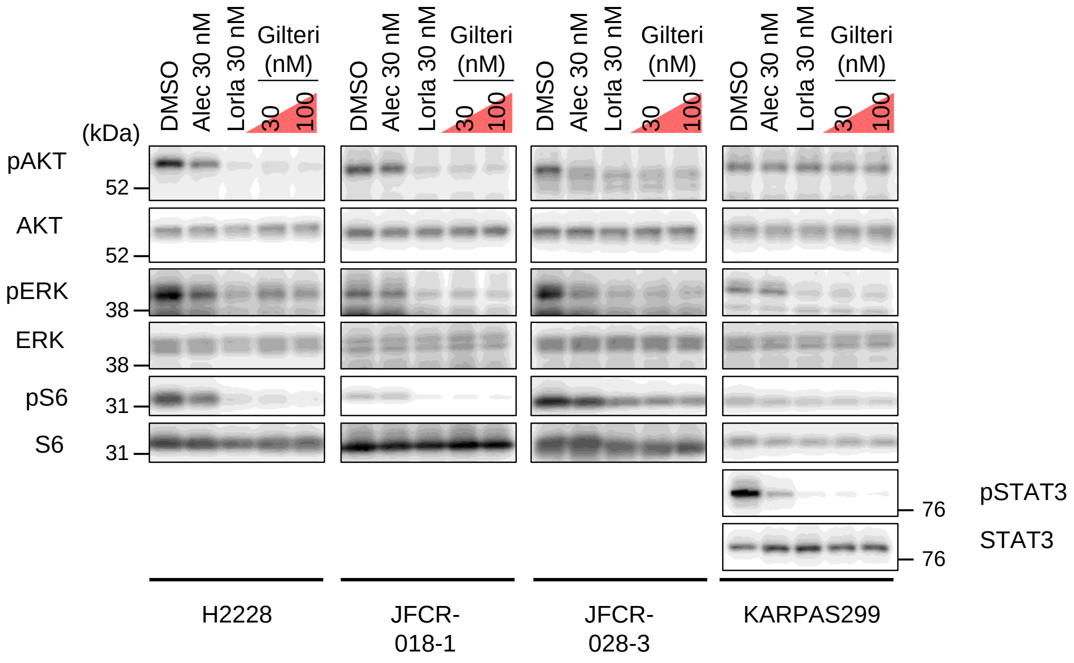

Supplementary Figure 5. Efficacy of gilteritinib in ALK positive cancer cell lines and patient derived primary cancer cell lines.

Suppression of ALK downstream signals in ALK positive cancer cell lines and patient derived primary cancer cell lines were evaluated by western blotting. Cells were treated with the indicated concentrations of gilteritinib for 6 h. Each of GAPDH is identical to Fig. 2B (n=2).

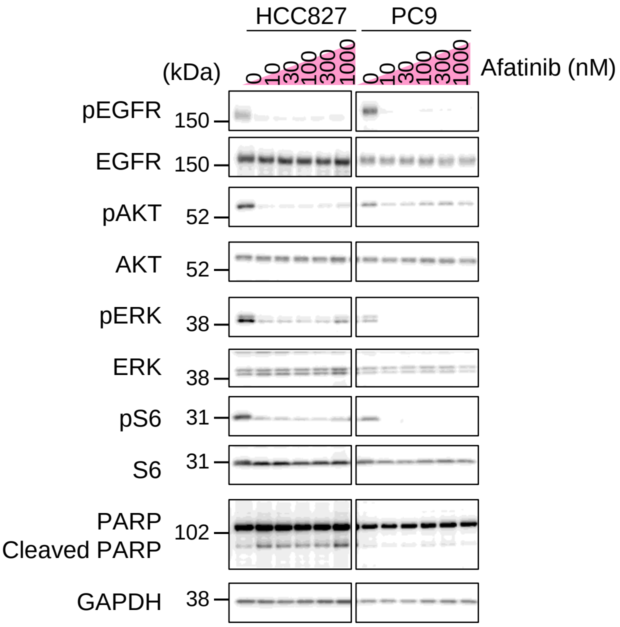

Supplementary Figure 6. Efficacy of Afatinib in EGFR positive cancer cell lines.

Suppression of phosphorylation of EGFR and its downstream signals in EGFR positive cancer cell were evaluated by western blotting. Cells were treated with the indicated concentrations of afatinib for 6 h (n=2).

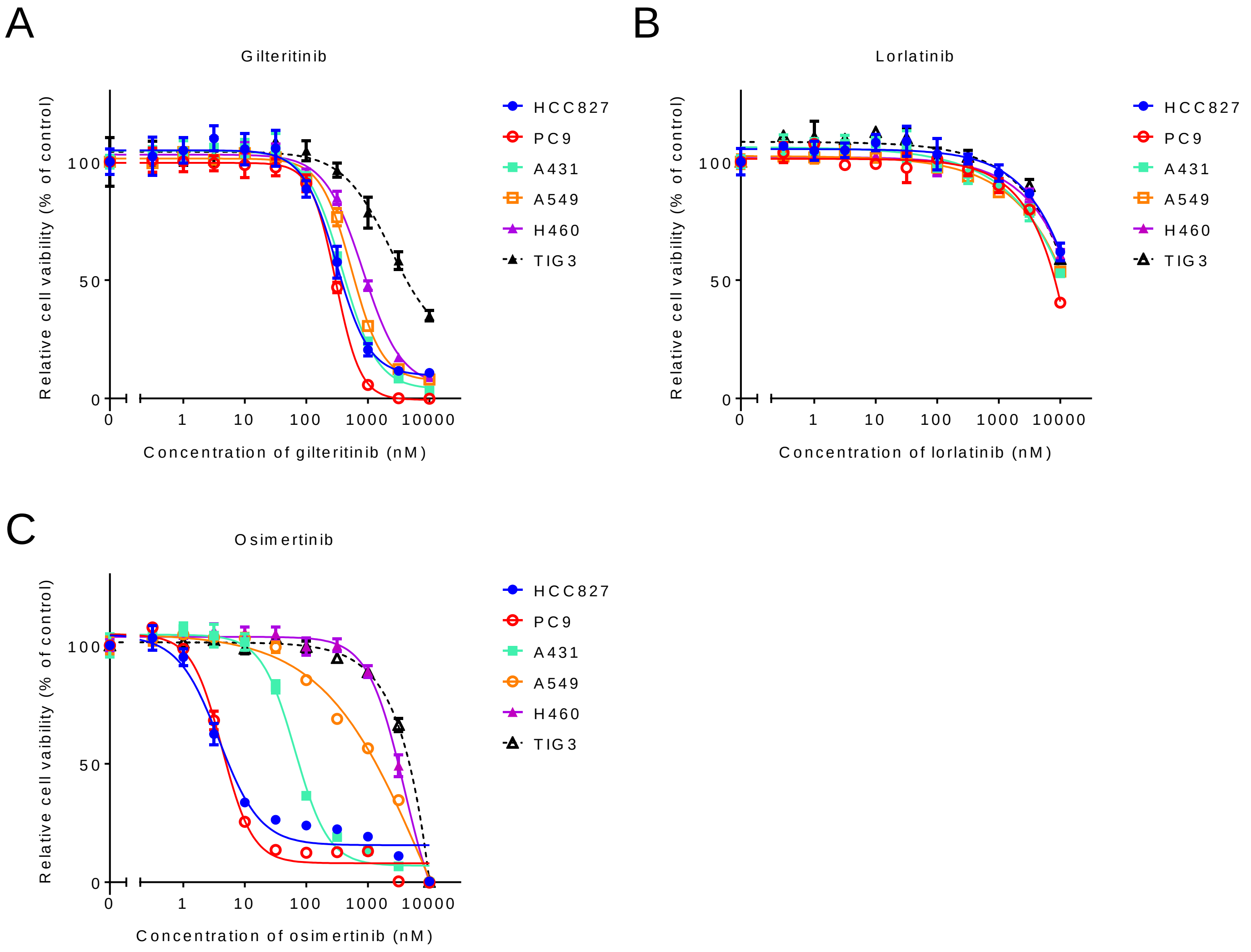

Supplementary Figure 7. Gilteritinib had the less activity in ALK negative NSCLC and TIG-3 cells.

(A-C) The inhibitory activity of gilteritinib in indicated NSCLC cancer cell lines and TIG-3 cells. Cells were treated with inhibitors for 72 h and analyzed cell viability using the CellTiter-Glo assay. N=3 independent samples examined over 3 independent experiments and representative experiment data are presented as mean values +/- SD.

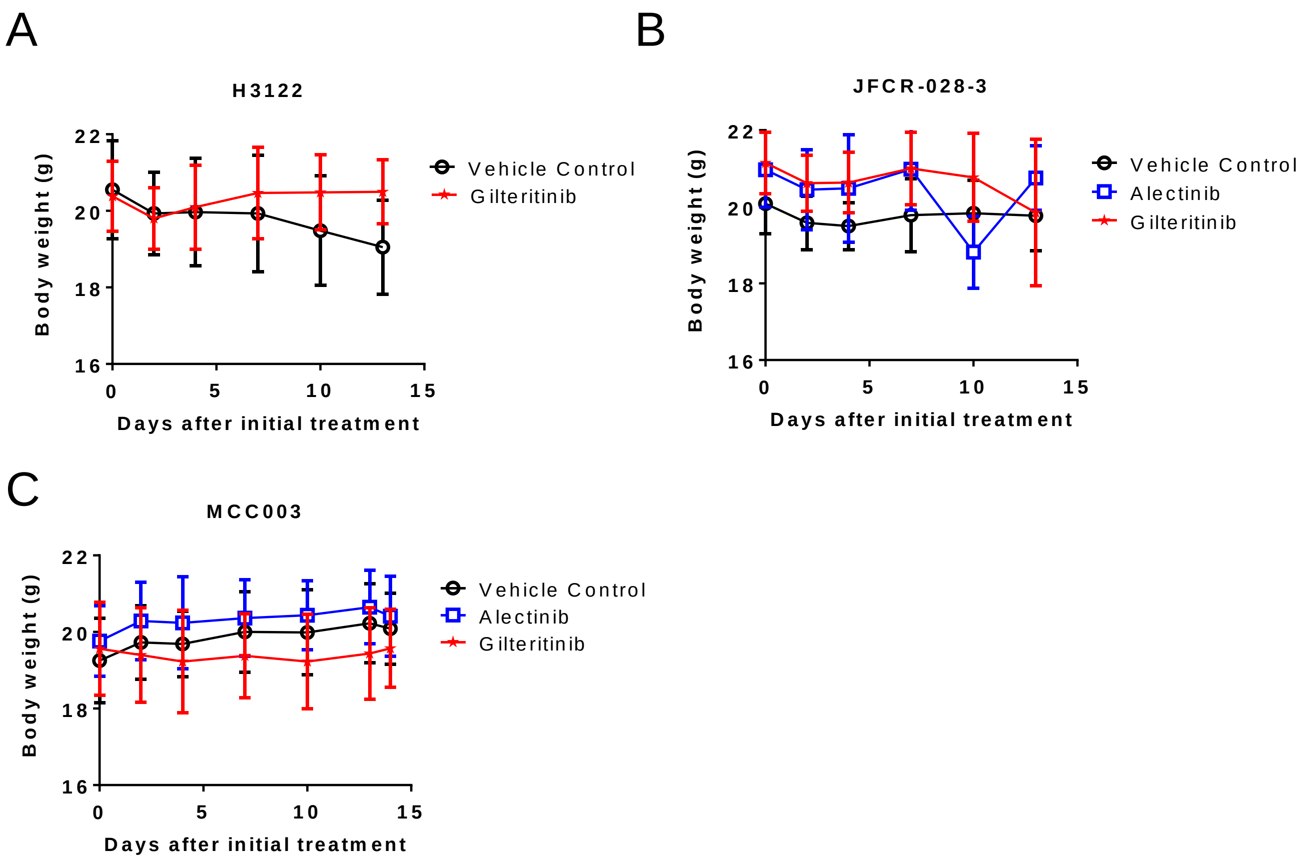

Supplementary Figure 8. Body weights were measured in TKIs treatment mice.

(A-C) H3122, JFCR-028-3 and MCC003 cells were subcutaneously transplanted into Balb/c *nu/nu* mice. When the average tumor volume reached ~150 mm^3, the mice were randomized into vehicle control, alectinib (30 mg/kg) or gilteritinib (30 mg/kg) treatment group. The mice were continuously treated once a day using oral gavage for 5 days/week (A,B: n=6 per treatment group, C: n=8 per treatment group). Body weights were measured three times a week. Data are presented as mean values +/- SD

A

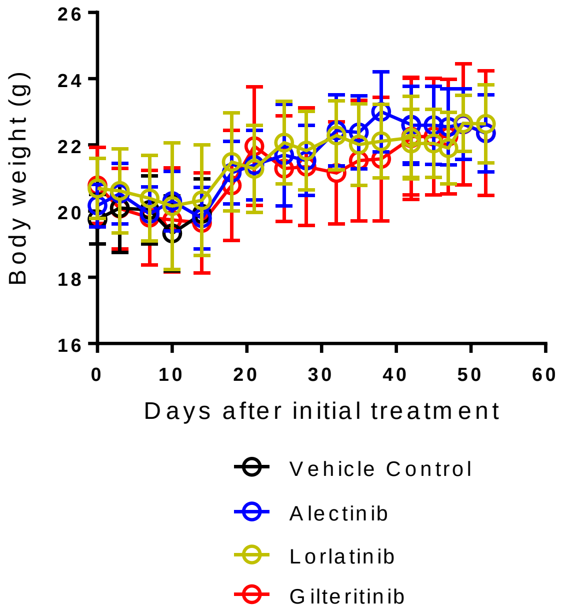

B

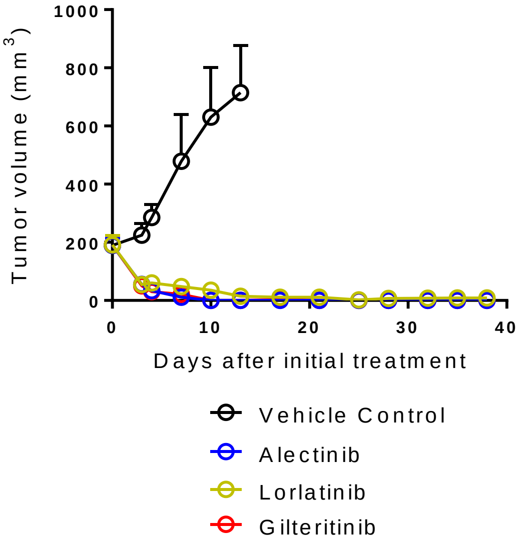

Supplementary Figure 9. Body weights were measured in TKIs treatment mice.

(A) EML4-ALK I1171N + F1174I mutant expressed JFCR-028-3 cells were subcutaneously transplanted into Balb/c *nu/nu* mice. When the average tumor volume reached ~200 mm^3, the mice were randomized into vehicle control, alectinib (30 mg/kg), lorlatinib (5 mg/kg), or gilteritinib (30 mg/kg) treatment group. The mice were continuously treated once a day using oral gavage for 5 days/week (n=6 per treatment group). Body weights were measured three times a week. At day 41, alectinib or lorlatinib treated mice were followed by administration of gilteritinib (30 mg/kg) for 5 days/week. Data are presented as mean values +/- SD (B) EML4-ALK WT expressed JFCR-028-3 cells were subcutaneously transplanted into Balb/c *nu/nu* mice. When the average tumor volume reached ~200 mm^3, the mice were randomized into vehicle control, alectinib (30 mg/kg), lorlatinib (5 mg/kg), or gilteritinib (30 mg/kg) treatment group. The mice were continuously treated once a day using oral gavage for 5 days/week (n=6 per treatment group). Data are presented as mean values + SD

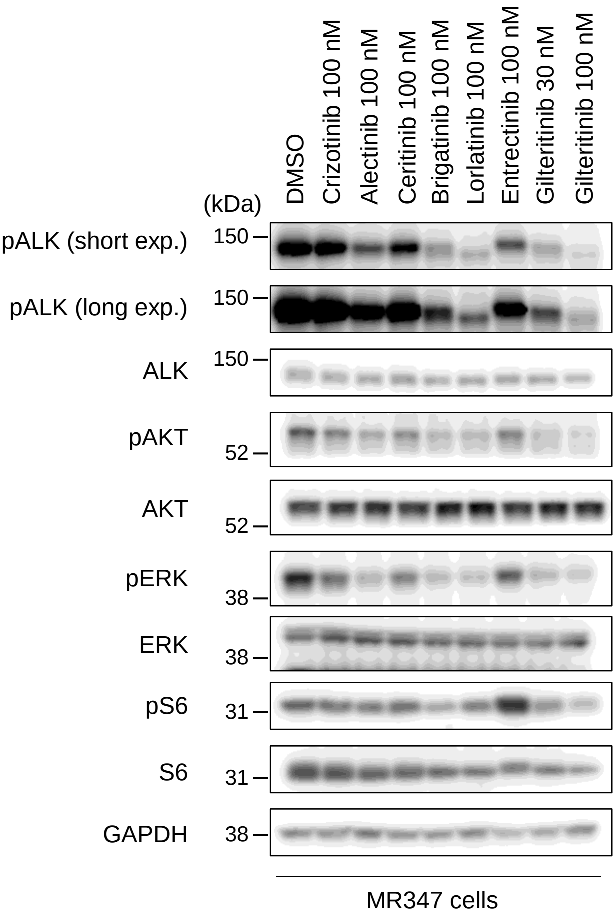

Supplementary Figure 10. Efficacy of gilteritinib in MR347 cells.

Phospho-ALK and its downstream signals in MR347 cells were evaluated by western blotting after the treatment of the indicated concentration of inhibitors for 6h (n=2).

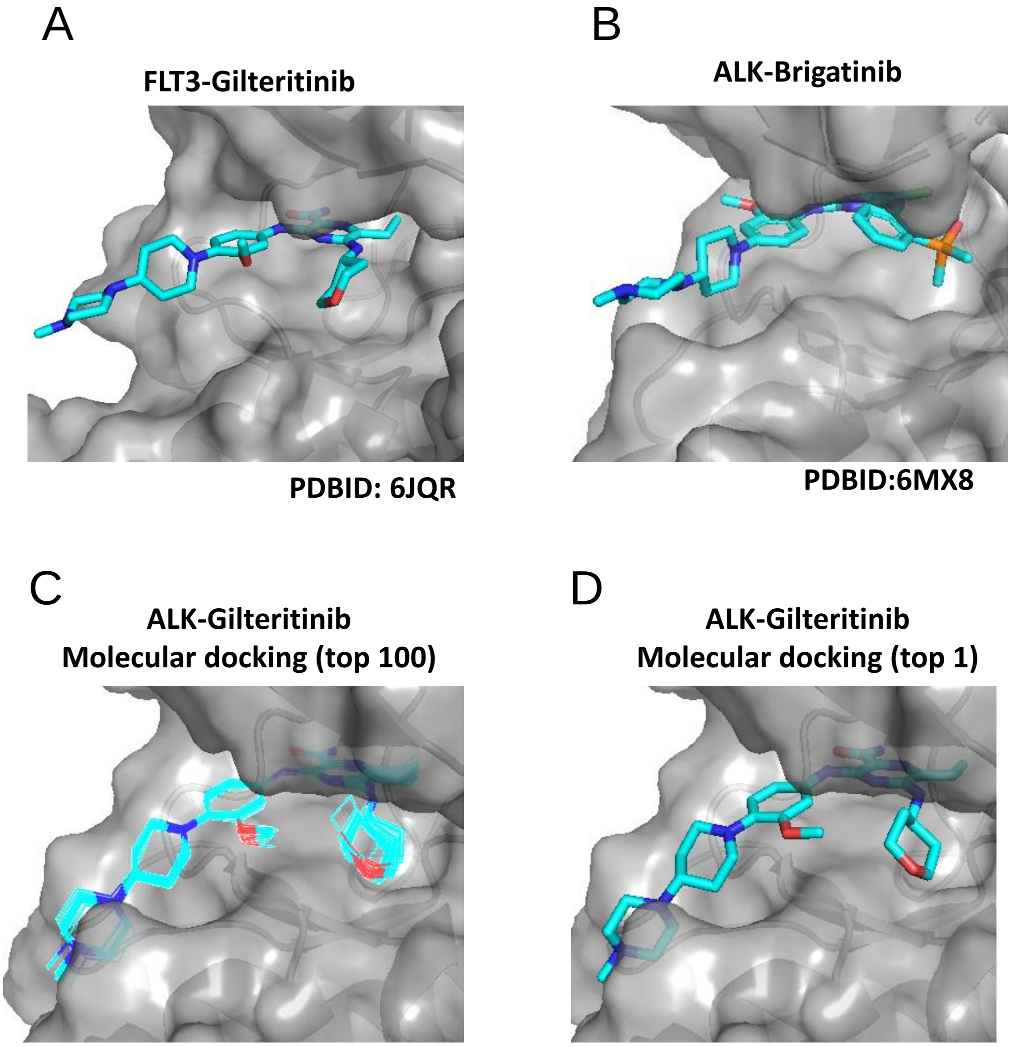

Supplementary Figure 11. Molecular docking of gilteritinib toward the ALK tyrosine kinase domain.

(A, B) Crystal structures of the (A) FLT3-gilteritinib (PDBID: 6JQR) and (B) ALK-brigatinib (PDBID: 6MX8) complexes. (C, D) Docking poses of gilteritinib. Molecular docking indicated that gilteritinib could fit into the ATP-binding site of ALK. A protocol of molecular docking was described in materials and methods. The top 100-ranked poses were superimposed in (C), and the highest scored pose was shown in (D), which was used as the initial structure of the molecular-dynamics simulation (shown in Fig 6A).

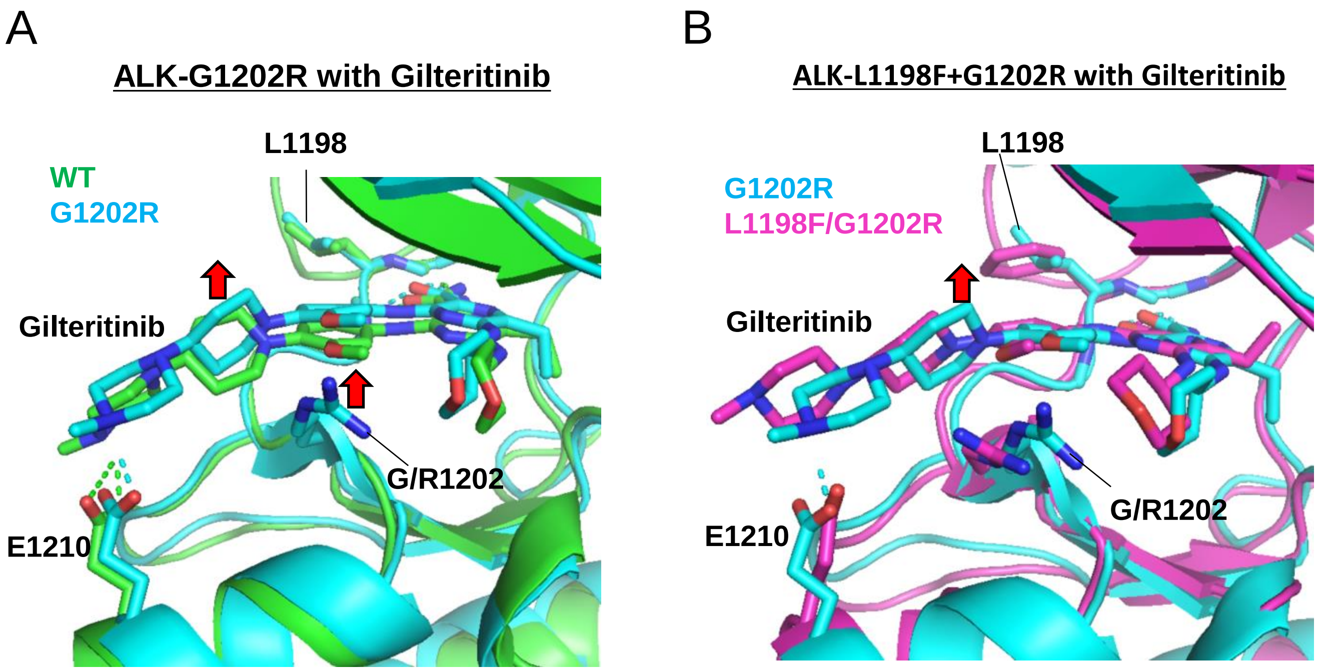

C

Estimated interaction energies of the complexes

	ΔG	coulomb	vdw
ALK (wild)-Gilteritinib (green)	- 21.03	- 4.57	- 16.46
G1202R-Gilteritinib (cyan)	- 18.32	- 1.96	- 16.36
L1198F+G1202R-Gilteritinib (magenta)	- 22.24	- 3.63	- 18.61

(kcal/mol)

Supplementary Figure 12. Computational prediction of the binding mode and binding affinity of gilteritinib toward ALK WT, G1202R, and L1198F+G1202R mutants.

(A, B) The mean stable structures of ALK-WT (green), G1202R (cyan), and L1198F + G1202R (magenta) in complex with gilteritinib were obtained from 50 ns $\times$5 MD simulations. The protein backbone is represented by a ribbon diagram, and gilteritinib and the side chains of L/F1198, G/R1202, and E1210 are depicted as sticks (C, green/cyan/magenta; N, blue; O, red). (C) The binding free energies (ΔG) of gilteritinib toward ALK WT, G1202R, and L1198F + G1202R mutants. Electrostatic (coulomb) and van der Waals (vdw) contributions in ΔG values are also indicated. The π- π interaction between gilteritinib and the side chain of F1198 might contribute to the higher affinity of gilteritinib for L1198F + G1202R than G1202R.

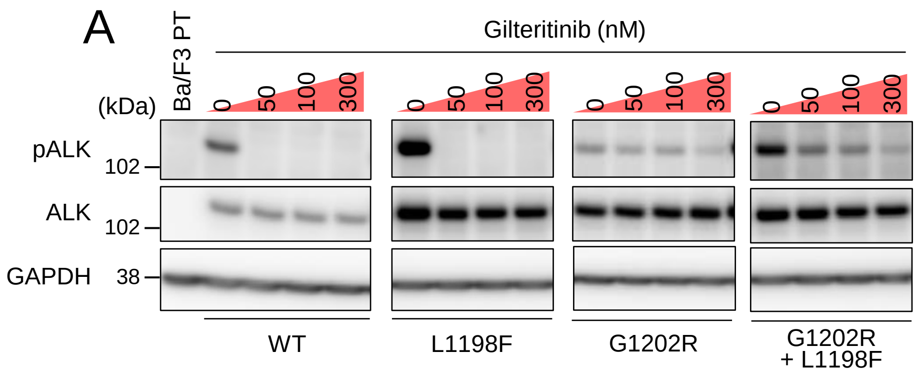

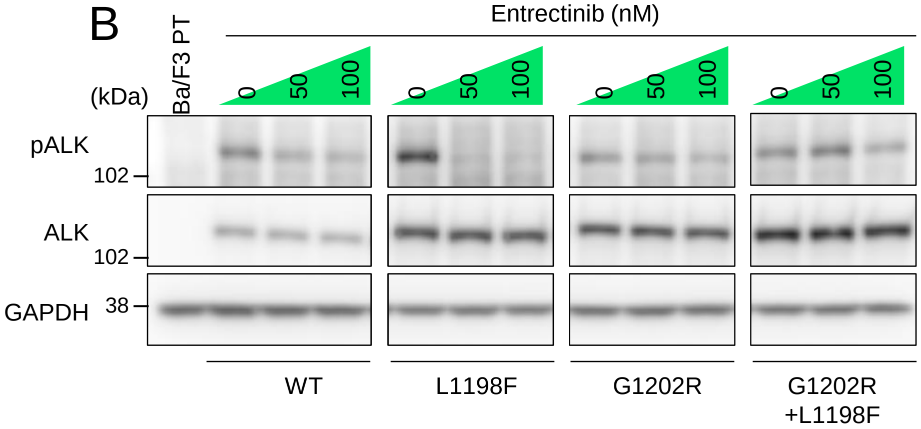

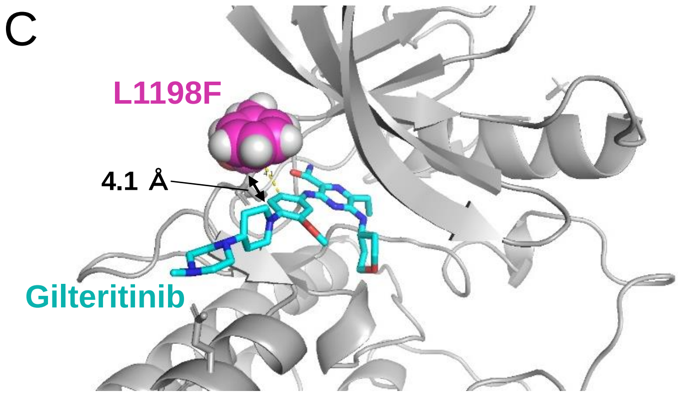

Supplementary Figure 13. ALK-L1198F mutation increase the sensitivity to gilteritinib.

(A) Suppression of phospho-ALK in each mutations expressing Ba/F3 cells were evaluated by western blotting. Cells were treated with the indicated concentrations of gilteritinib for 3 h. The data of G1202R mutant is same as the data in Fig 2E (n=2). (B) Phospho-ALK in each EML4-ALK mutations expressing Ba/F3 cells were evaluated by western blotting. Cells were treated with the indicated concentrations of entrectinib for 3 h (n=2). (C) The mean stable structure of the ALK (L1198F)-gilteritinib complex was obtained from 50 ns ×5 MD simulations. The protein backbone is represented by a grey ribbon diagram and gilteritinib is depicted as sticks (C, light blue; N, blue; O, red). The side chain of F1198 is depicted as magenta spheres.

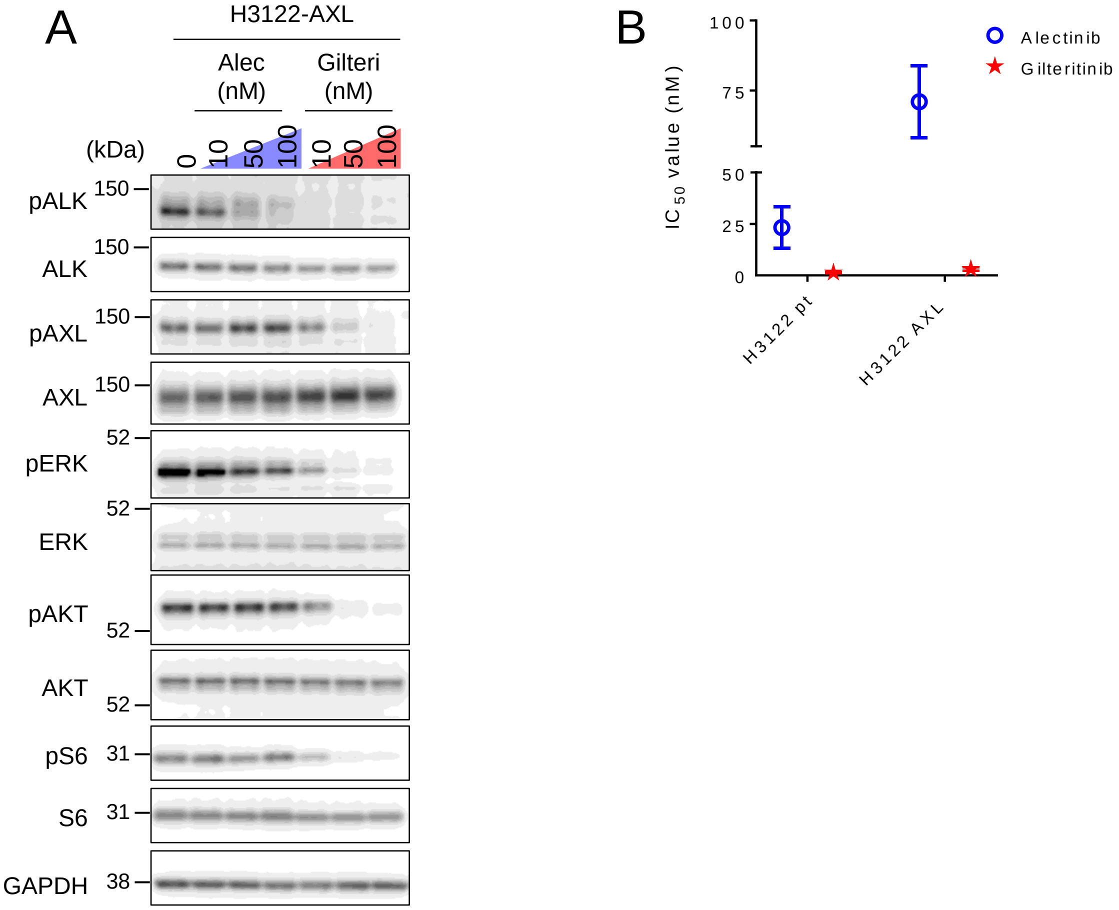

Supplementary Figure 14. Efficacy of gilteritinib against AXL overexpressed H3122 cells. **(**A) The suppression of phospho-ALK, phospho-AXL, and its downstream signals in AXL expressing H3122 cells was evaluated via western blotting. Cells were treated with the indicated concentrations of alectinib or gilteritinib for 6 h (n=2). (B) IC_{50} calculated from the viability analysis of parental or AXL expressing H3122 cells. Cells were treated with alectinib or gilteritinib for 72 h. N=3 independent samples examined over 3 independent experiments and data presented as mean values +/- SD.

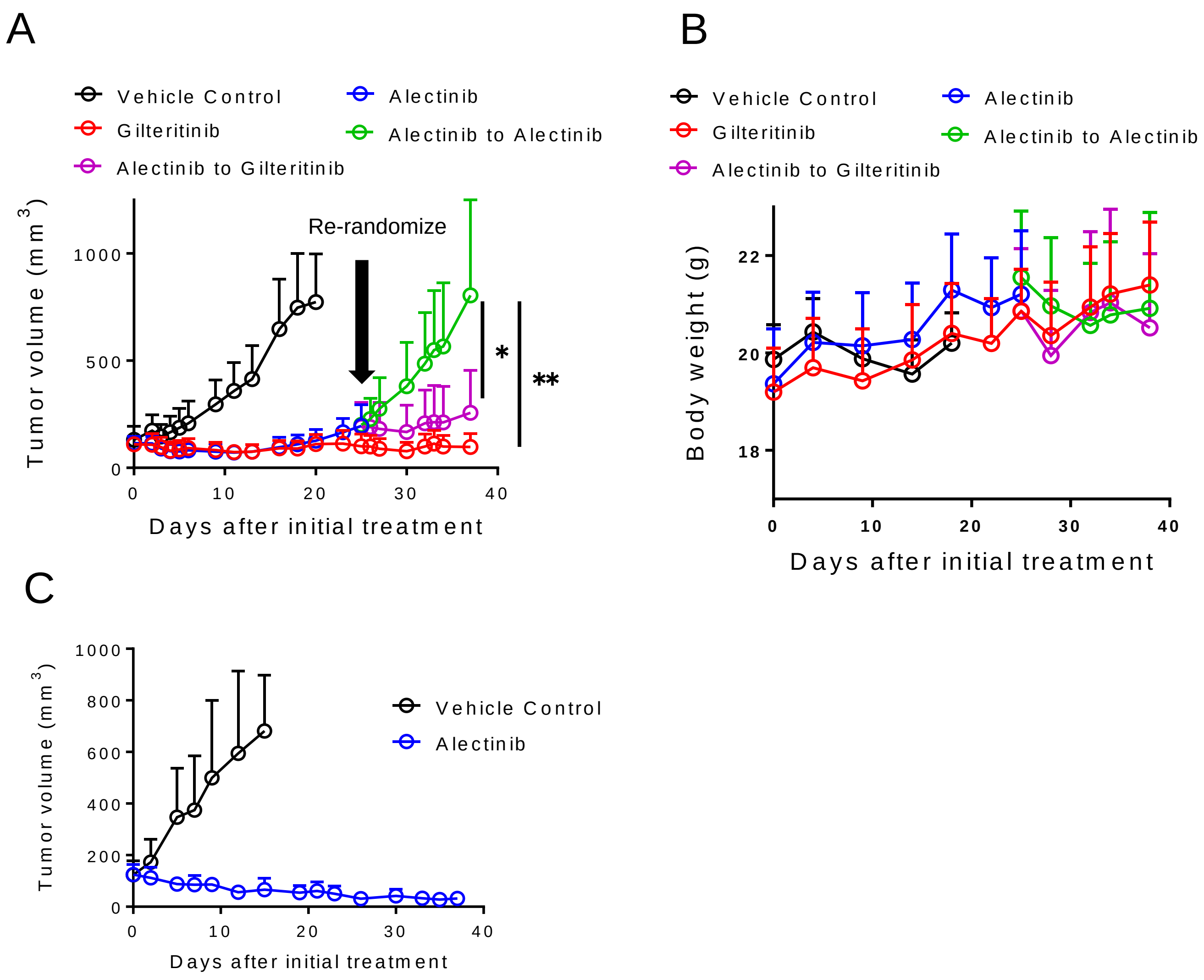

Supplementary Figure 15. In vivo study for AXL overexpressed H3122 cells.

(A,B) AXL overexpressed H3122 cells were subcutaneously transplanted into Balb/c *nu/nu* mice. When the average tumor volume reached ~150 mm^3, the mice were randomized into vehicle control, alectinib (30 mg/kg) or gilteritinib (30 mg/kg) treatment group. The mice were continuously treated once a day using oral gavage for 5 days/week (n=6 per treatment group). Tumor volumes (A) and body weights (B) were measured three times a week. At day 25, alectinib treated mice were re-randomized and followed by continuous administration of alectinib (30 mg/kg) or gilteritinib (30 mg/kg) for 6 days/week. The significance of differences on day 37 was calculated using the two-sided Mann–Whitney U test (*P* value: Alectinib to alectinib vs alectinib to gilteritinib treatment group, 0.026; Alectinib to alectinib vs gilteritinib treatment group, 0.0012). Data are presented as mean values + SD. (C) H3122 parental cells were subcutaneously transplanted into Balb/c *nu/nu* mice. When the average tumor volume reached ~150 mm^3, the mice were randomized into vehicle control or alectinib (30 mg/kg) treatment group. The mice were continuously treated once a day using oral gavage for 5 days/week (n=6 per treatment group). Data are presented as mean values + SD

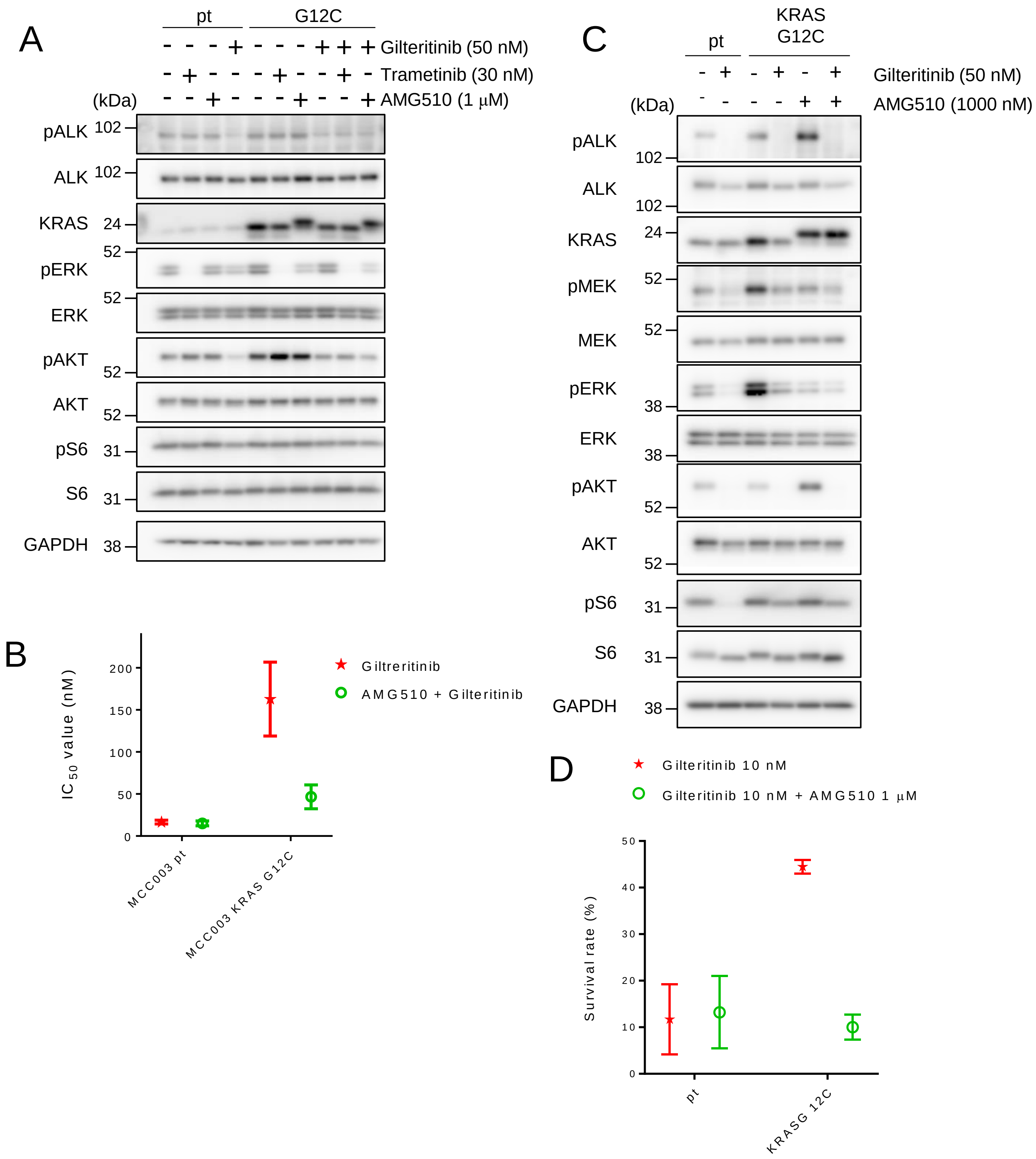

Supplementary Figure 16. The efficacy of gilteritinib or combination therapy with gilteritinib and AMG510 against KRAS G12C overexpressed ALK positive patient derived cells.

(A) The suppression of phospho-ALK and its downstream signals in KRAS G12C expressing MCC-003 cells were evaluated via western blotting. Cells were treated with the indicated concentrations of drugs for 6 h (n=2). (B) IC_{50} calculated from the viability analysis of parental or KRAS G12C expressing MCC-003 cells. Cells were treated with gilteritinib or combination with gilteritinib and AMG510 for 72 h. N=3 independent samples examined over 3 independent experiments and data presented as mean values +/- SD. (C) Suppression of phospho-ALK and its downstream signals in KRAS G12C overexpressed JFCR-028-3 cells were evaluated by western blotting. Cells were treated with the indicated concentrations of TKIs for 6 h (n=2). (D) The inhibitory activity of TKIs in KRAS G12C overexpressed JFCR-028-3 cells. Cells were treated with gilteritinib 10 nM and with or without AMG510 1 μM for 72 h. Cell viability was analyzed by using the CellTiter-Glo assay. N=3 independent samples examined over 3 independent experiments and data presented as mean values +/- SD.

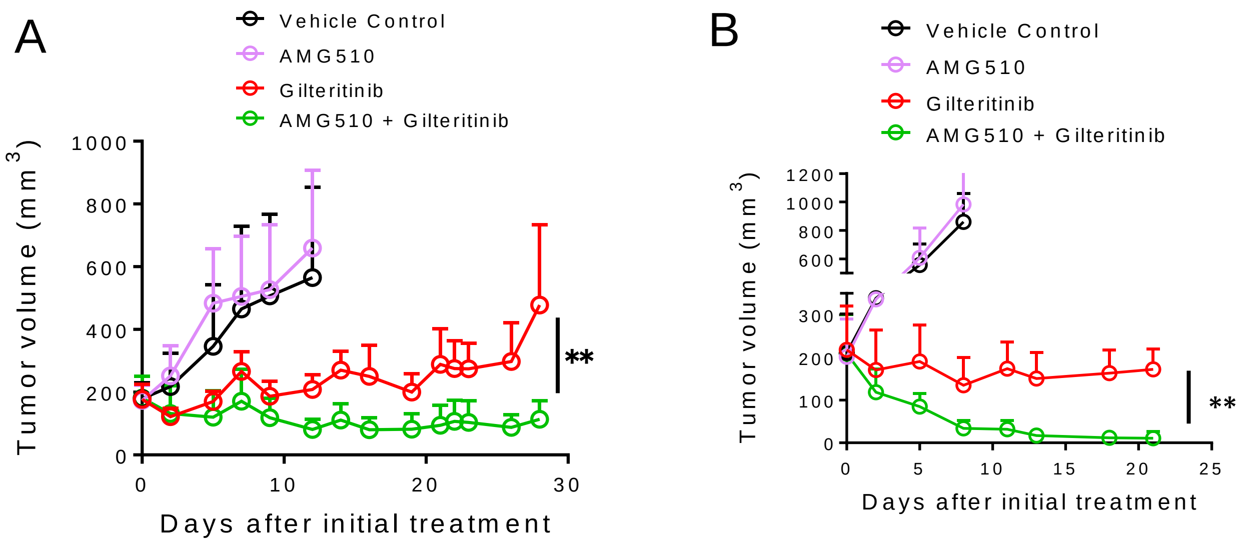

Supplementary Figure 17. The efficacy of gilteritinib or combination therapy with gilteritinib and AMG510 against KRAS G12C overexpressed ALK positive tumor.

(A) KRAS G12C expressing MCC-003 cells were subcutaneously transplanted into BALB/c *nu/nu* mice. When the average tumor volume reached approximately 175 mm^3, the mice were randomized to treatment with vehicle control, AMG510 (100 mg/kg), gilteritinib (30 mg/kg) or combined with AMG510 (100 mg/kg) and gilteritinib (30 mg/kg) treatment group once daily for 5 days/week via oral gavage (n=6 per treatment group). Tumor volumes were measured three times a week. The significance of differences on day 28 was calculated using the two-sided Mann–Whitney *U* test (*P* value: Gilteritinib vs AMG510 + gilteritinib treatment group, 0.0043). Data are presented as mean values + SD. (B) KRAS G12C expressed JFCR-028-3 cells were subcutaneously transplanted into BALB/c nu/nu mice. When the average tumor volume reached approximately 200 mm^3, the mice were randomized to treatment with vehicle control, AMG510 (100 mg/kg), gilteritinib (30 mg/kg) or combined with AMG510 (100 mg/kg) and gilteritinib (30 mg/kg) treatment group once daily for 5 days/week via oral gavage. Tumor volumes were measured three times a week (n=6 per treatment group). The significance of differences on day 21 was calculated using the two-sided Mann–Whitney U test (*P* value: Gilteritinib vs AMG510 + gilteritinib treatment group, 0.0022). Data are presented as mean values + SD.

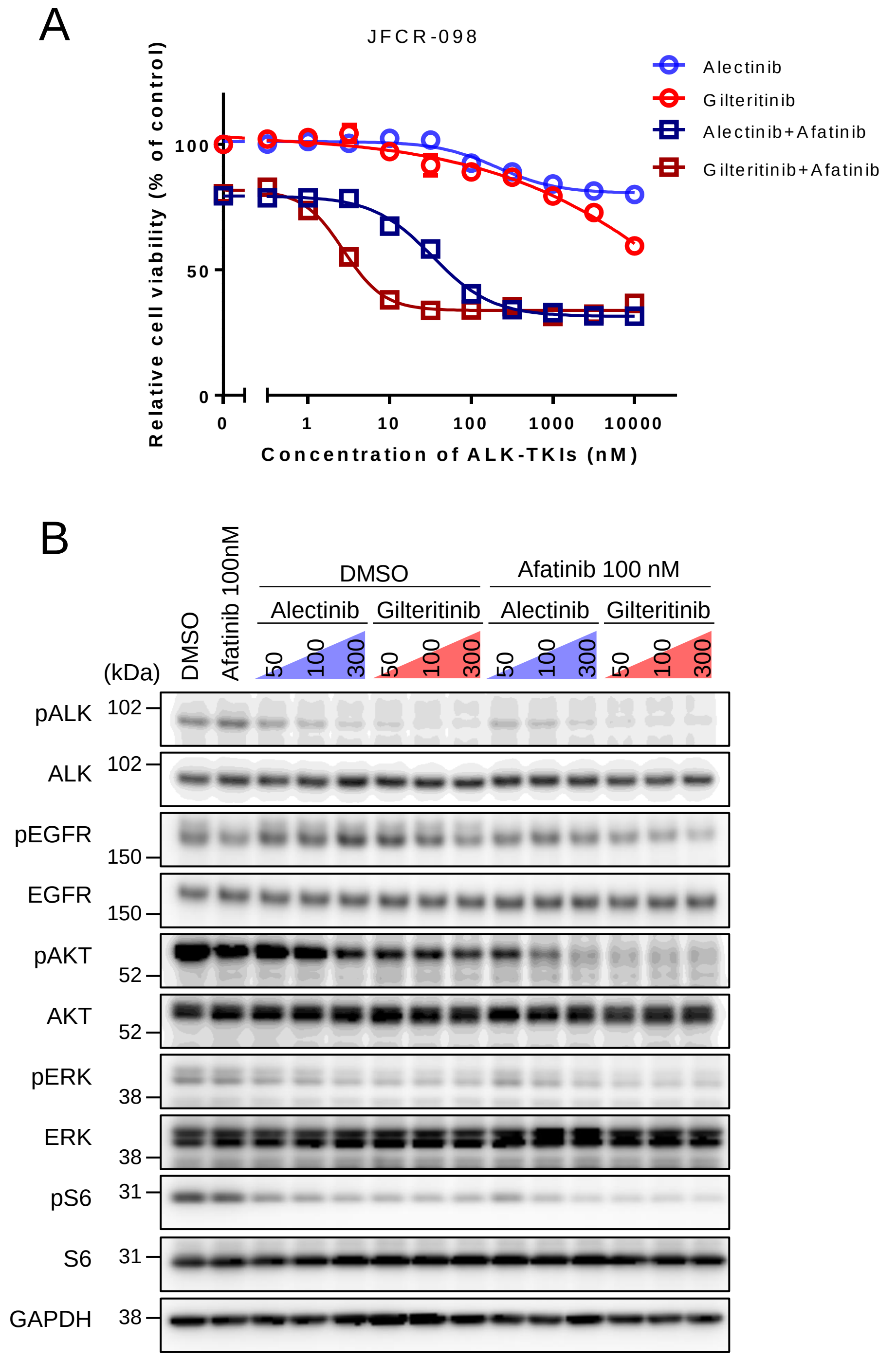

Supplementary Figure 18. The efficacy of gilteritinib or combination therapy with gilteritinib and afatinib.

(A) The inhibitory activity of TKIs in JFCR-098 cells. Cells were treated with inhibitors for 72 h and analyzed cell viability using the CellTiter-Glo assay. Afatinib was consistently treated at concentration of 100 nM for combination treatment. N=3 independent samples examined over 3 independent experiments and representative experiment data are presented as mean values +/- SD. (B) Suppression of phospho-ALK, phospho-EGFR and its downstream signals in JFCR-098 cells were evaluated by western blotting. Cells were treated with the indicated concentrations of TKIs for 6 h (n=2).

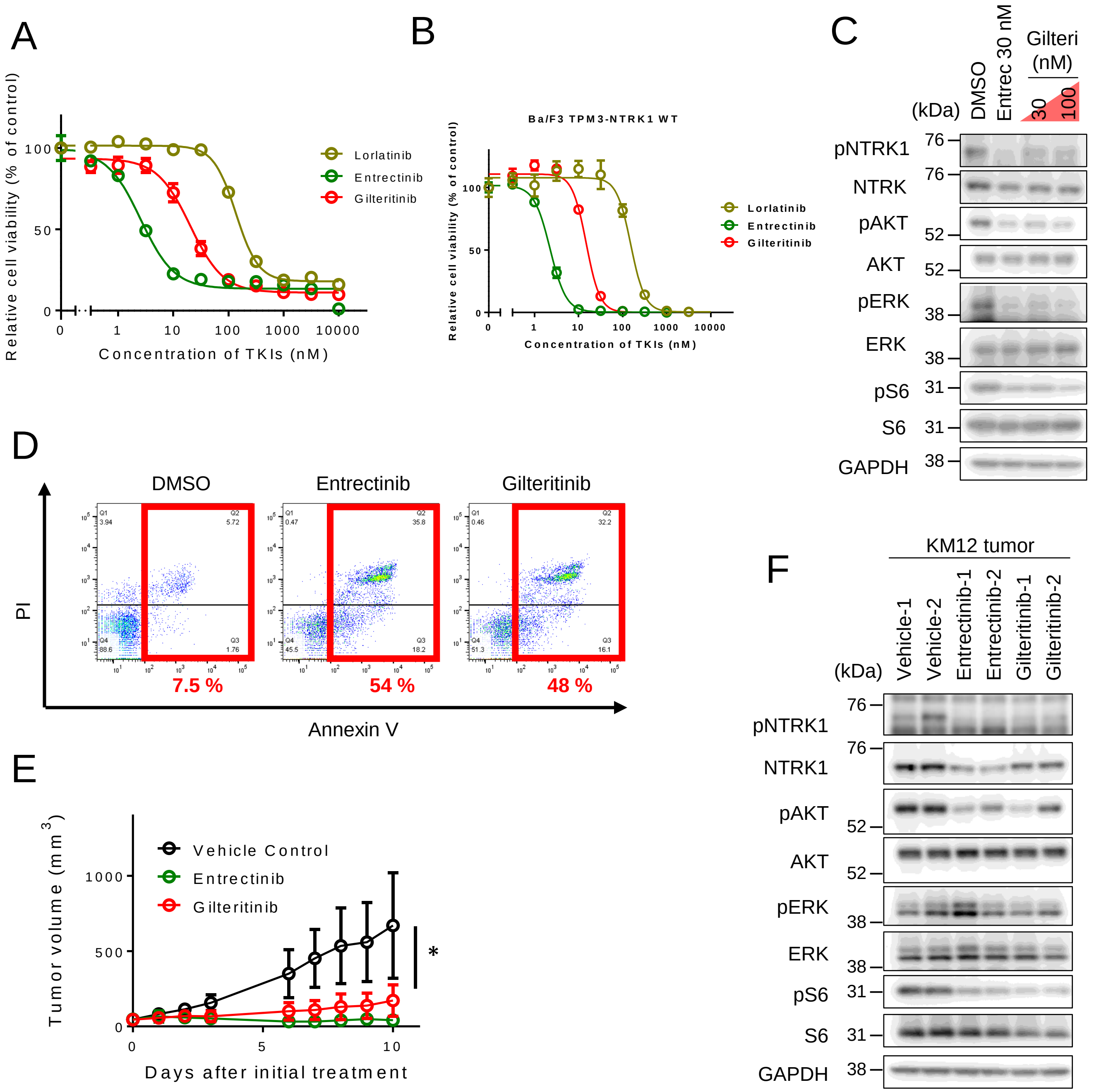

Supplementary Figure 19. Efficacy of gilteritinib in KM12 cells.

(A,B) The activity of the indicated inhibitors in KM12 cells (A) and Ba/F3 cells expressing TPM3-NTRK1 wild-type (B). Cells were treated with serially diluted inhibitors for 72 h, and viability was analyzed using the CellTiter-Glo assay. N=3 independent samples examined over 3 independent experiments and representative experiment data are presented as mean values +/- SD. (C) Suppression of phospho-NTRK1 and its downstream signals in KM12 cells were evaluated by western blotting. Cells were treated with the entrectinib or gilteritinib for 6 h (n=2). (D) Apoptosis assay by flow cytometry analysis with Annexin-V and PI staining after 72 h of 100 nM entrectinib or gilteritinib treatment to KM12 cells. The percentage of cells undergoing apoptosis is shown in red. (E) KM12 cells were subcutaneously transplanted into BALB/c *nu/nu* mice. When the average tumor volume reached approximately 100 mm^3, the mice were randomized to treatment with vehicle control, entrectinib (30 mg/kg), or gilteritinib (30 mg/kg) treatment group once daily for 10 days via oral gavage (n=6 per treatment group). Tumor volumes were measured three times a week. The significance of differences on day 10 was calculated using the two-sided Mann–Whitney U test (*P* value: Vehicle vs gilteritinib treatment group, 0.026). Data are presented as mean values +/- SD (F) Phospho-NTRK1 and its downstream signals in KM12 tumor samples obtained from vehicle-, entrectinib-, or gilteritinib-treated mice were evaluated via western blotting (n=2).

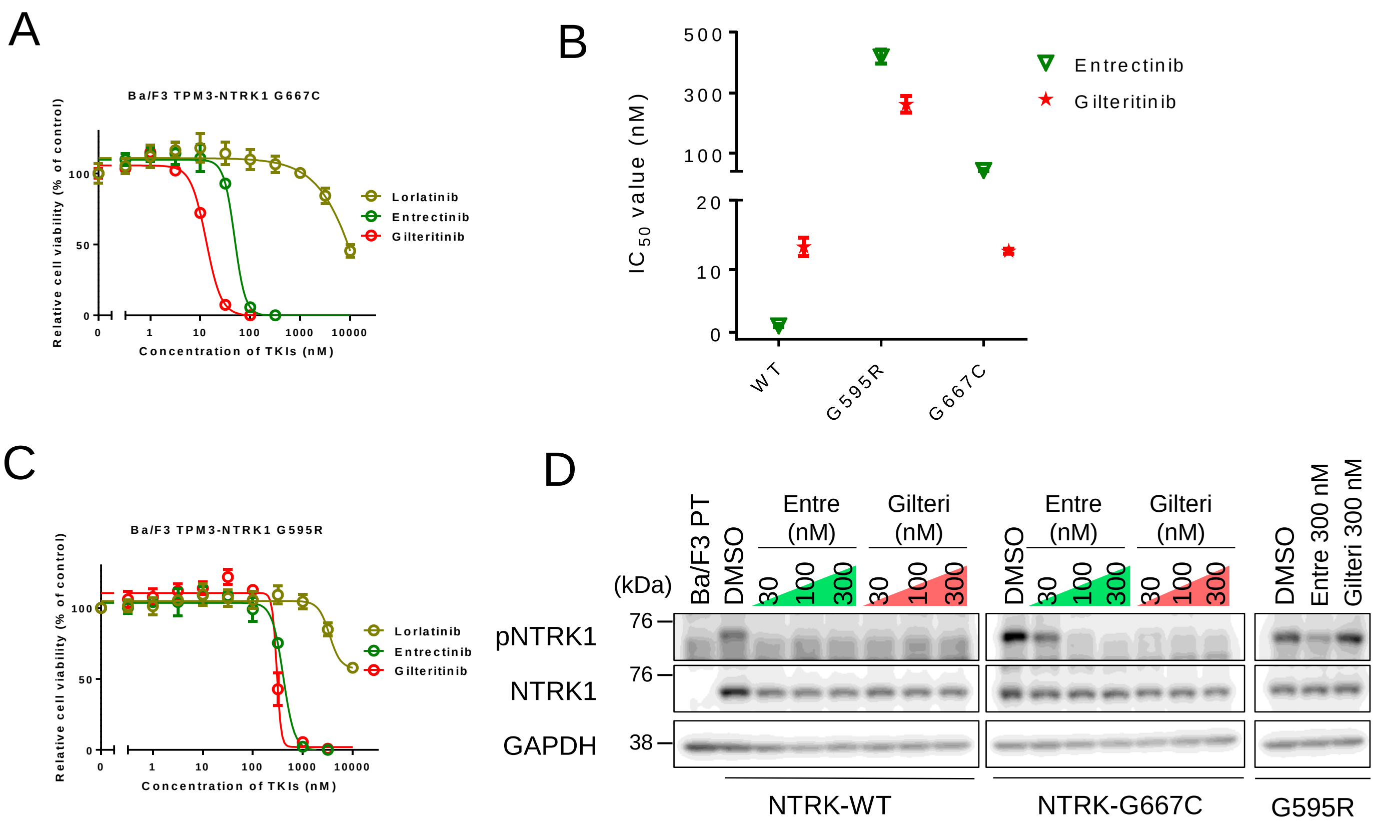

Supplementary Figure 20. NTRK1-G667C mutant is more sensitive to gilteritinib.

(A) The activity of the indicated inhibitors in Ba/F3 cells expressing TPM3-NTRK1 G667C. Cells were treated with the inhibitors for 72 h, and viability was analyzed using the CellTiter-Glo assay. N=3 independent samples examined over 3 independent experiments and representative experiment data are presented as mean values +/- SD. (B) IC_{50} calculated from the viability analysis of Ba/F3 cells carrying indicated TPM3-NTRK1. Cells were treated with entrectinib or gilteritinib for 72 h. N=3 independent samples examined over 3 independent experiments and data presented as mean values +/- SD. (C) The activity of the indicated inhibitors in Ba/F3 cells expressing TPM3-NTRK1 G595R. Cells were treated with the inhibitors for 72 h, and viability was analyzed using the CellTiter-Glo assay. N=3 independent samples examined over 3 independent experiments and representative experiment data are presented as mean values +/- SD. (D) Phospho-NTRK1 expression in the indicated TPM3-NTRK1 mutation-expressing Ba/F3 cells was evaluated via western blotting. Cells were treated with the indicated concentrations of entrectinib or gilteritinib for 3 h (n=2).

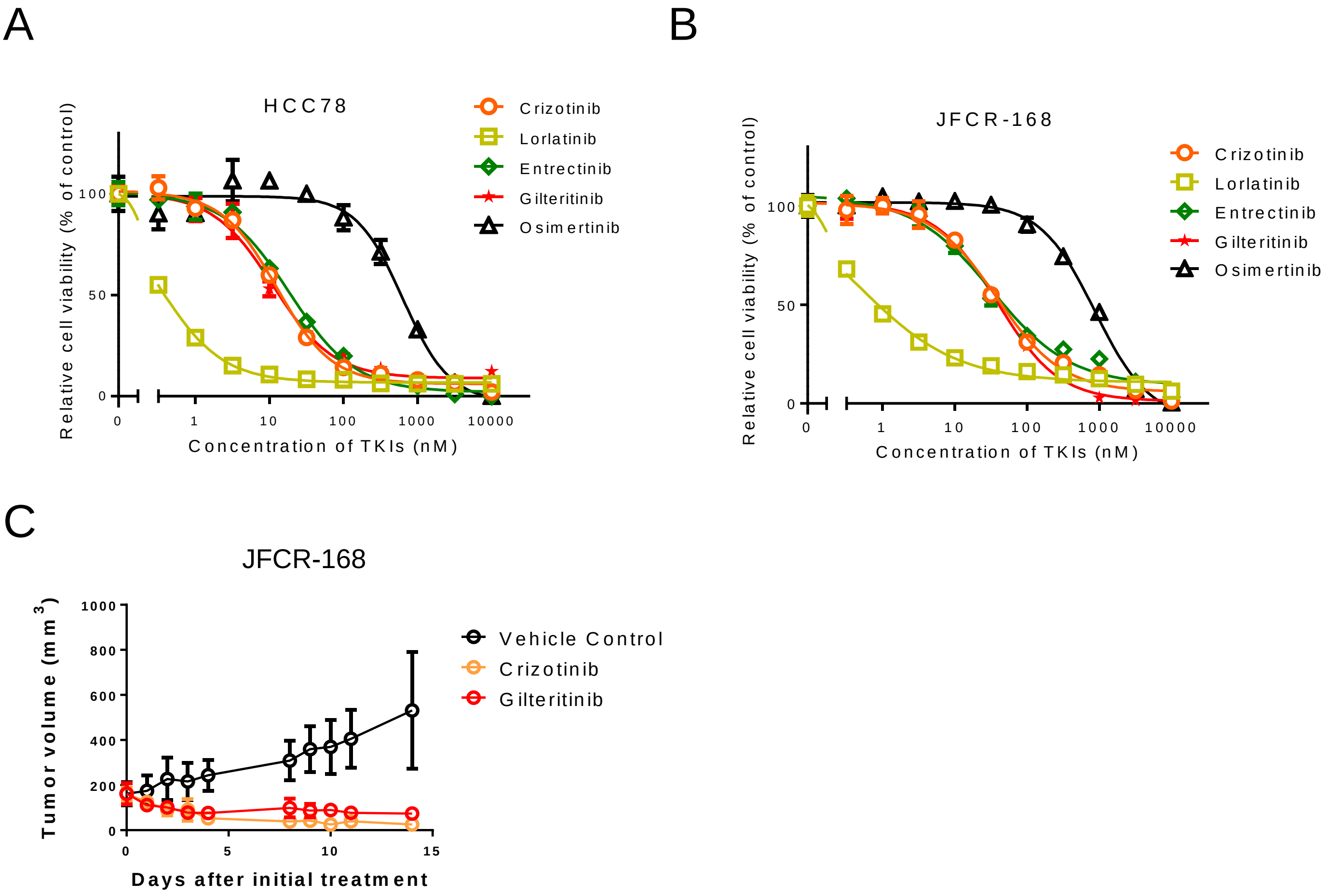

Supplementary Figure 21. Efficacy of gilteritinib in ROS1 positive NSCLC.

(A,B) The inhibitory activity of indicated inhibitors in HCC78 (A) and JFCR-168 cells (B). Cells were treated with inhibitors for 72 h and analyzed cell viability using the CellTiter-Glo assay. N=3 independent samples examined over 3 independent experiments and representative experiment data are presented as mean values +/- SD. (C) JFCR-168 cells were subcutaneously transplanted into SCID-Beige mice. When the average tumor volume reached approximately 150 mm^3, the mice were randomized to treatment with vehicle control or Crizotinib (100 mg/kg) or gilteritinib (30 mg/kg) treatment group once daily for 5 days/week via oral gavage (n=3 per treatment group). Tumor volumes were measured three times a week. Data are presented as mean values +/- SD

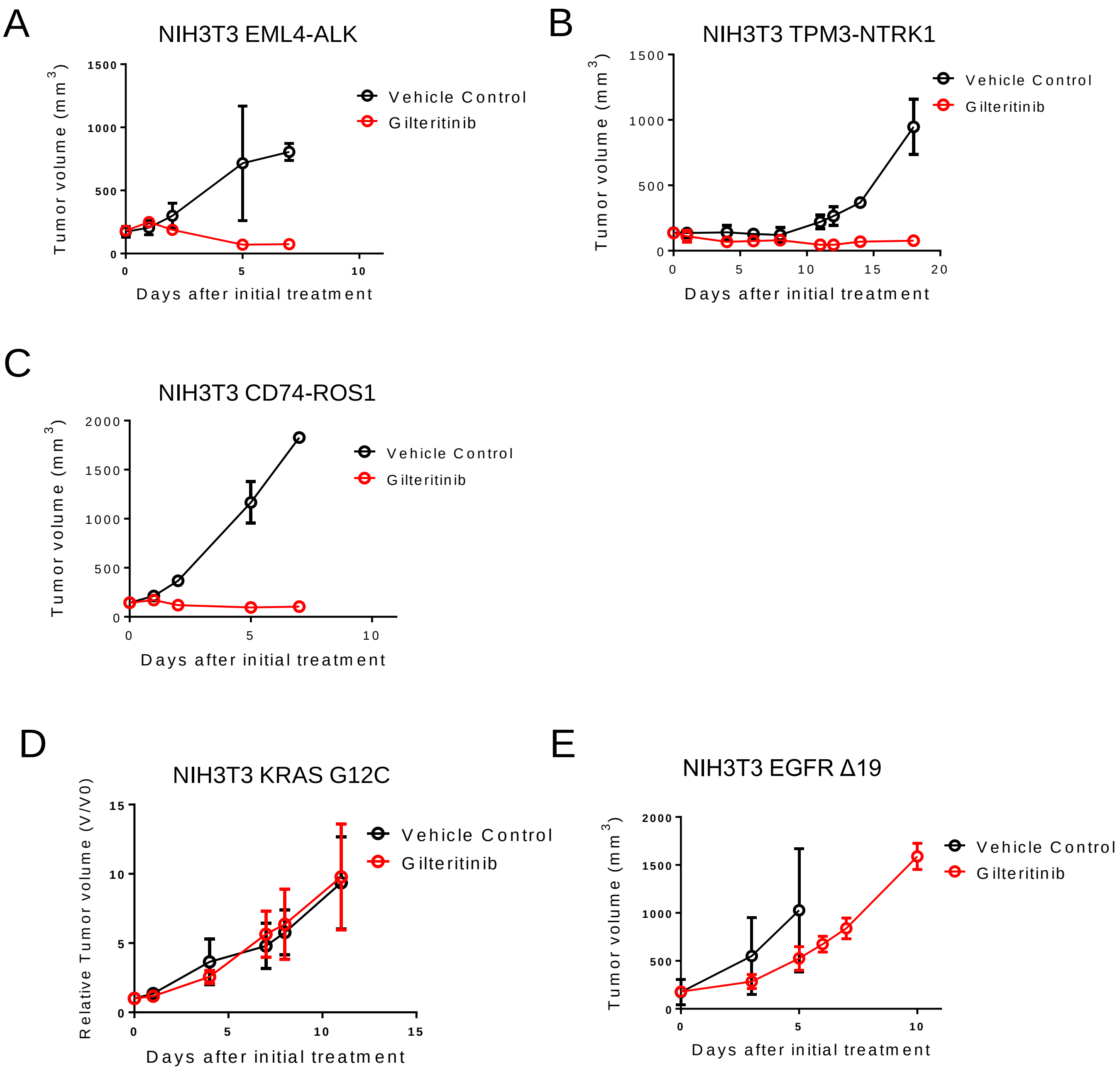

Supplementary Figure 22. Efficacy of gilteritinib in each oncogene.

(A-E) NIH3T3 cells carrying EML4-ALK (A), TPM3-NTRK1 (B), CD74-ROS1 (C), KRAS G12C (D), or EGFR Δ19 (E) were subcutaneously transplanted into BALB/c *nu/nu* mice. When the average tumor volume reached approximately 150 mm^3, the mice were randomized to treatment with vehicle control or gilteritinib (30 mg/kg) treatment group once daily for 5 days/week via oral gavage (n=3 per treatment group). Tumor volumes were measured three times a week. Data are presented as mean values +/- SD

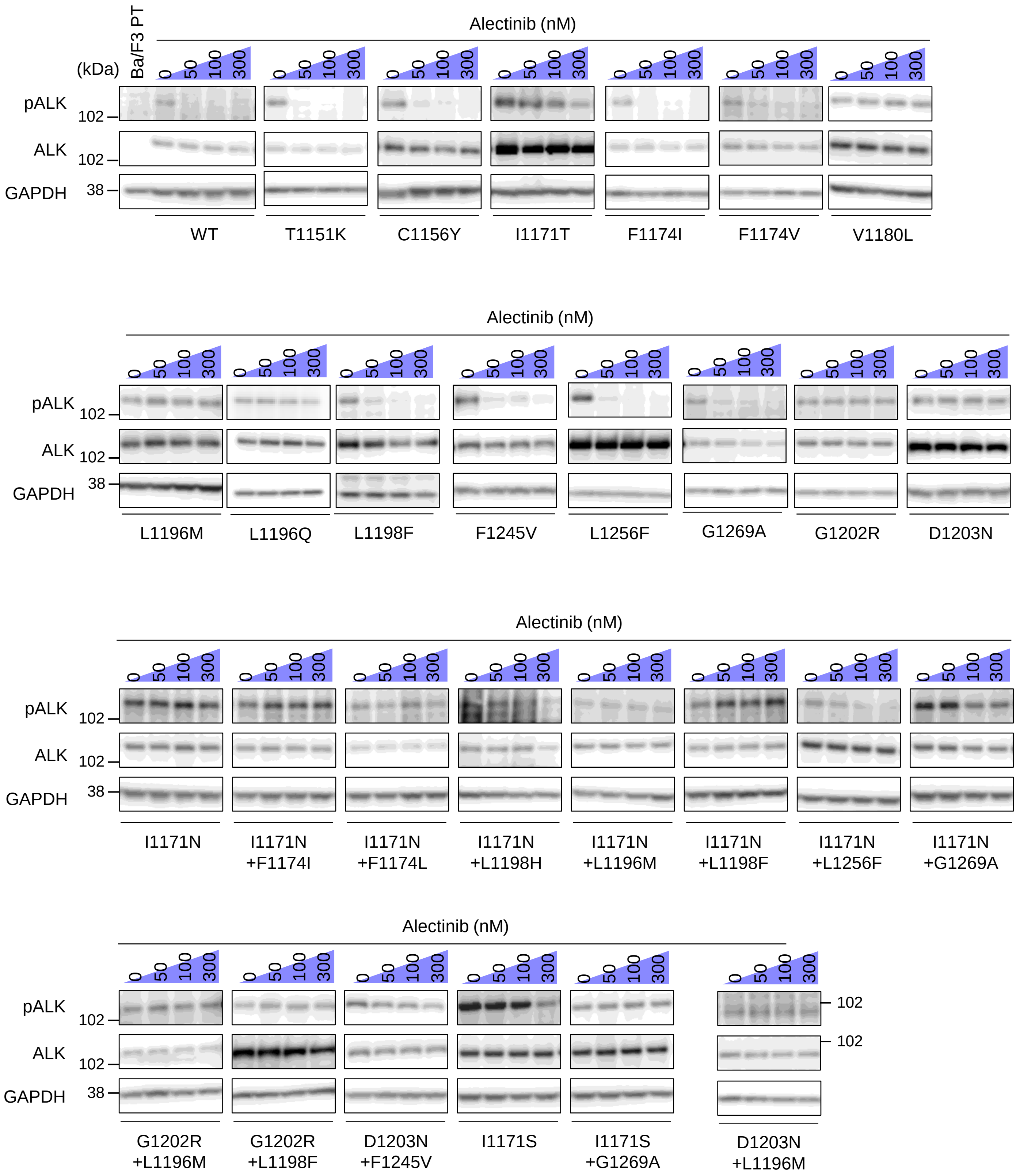

Supplementary Figure 23. Efficacy of alectinib in EML4-ALK mutations expressing Ba/F3 cells.

Phospho-ALK in each EML4-ALK mutations expressing Ba/F3 cells were evaluated by western blotting. Cells were treated with the indicated concentrations of alectinib for 3 h (n=2).

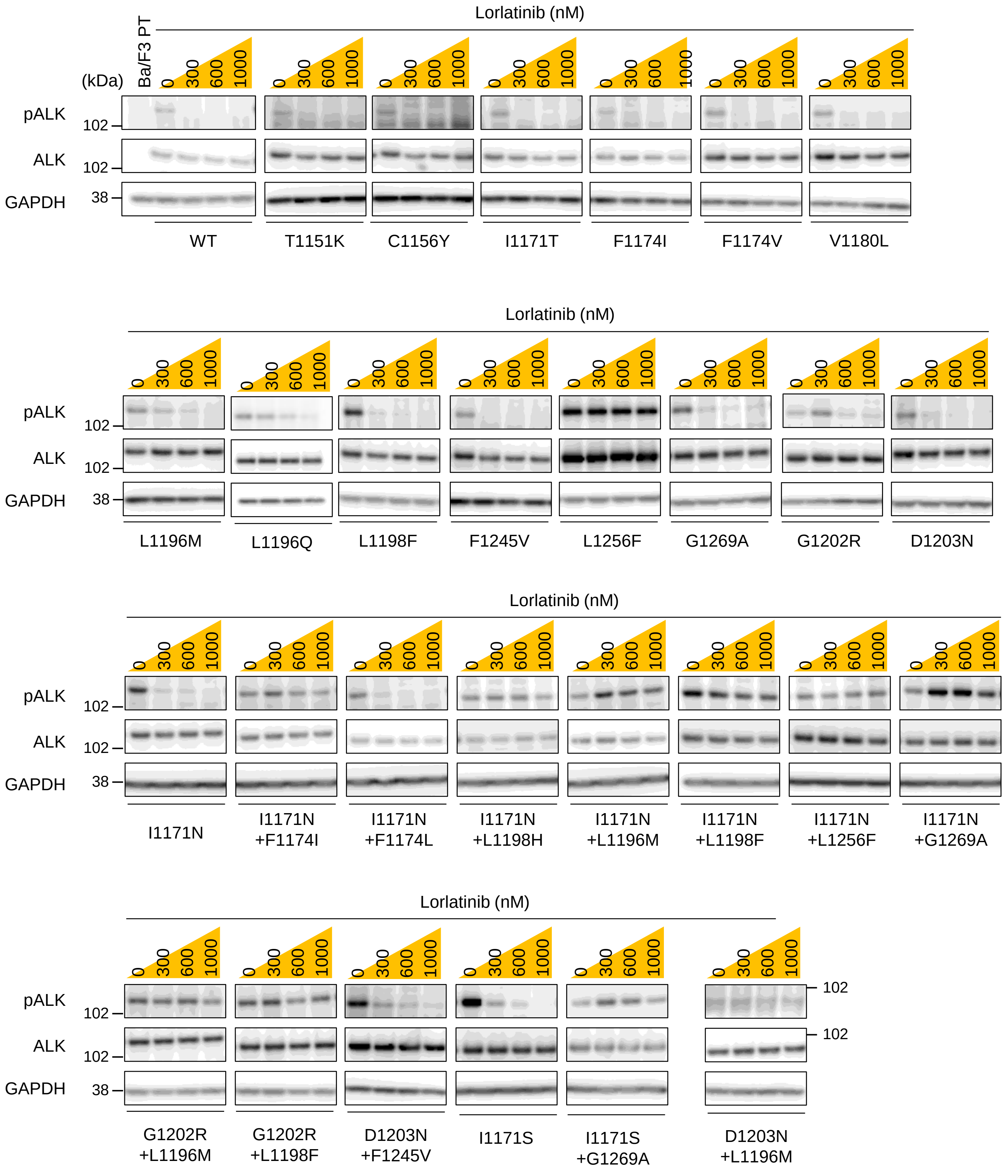

Supplementary Figure 24. Efficacy of lorlatinib in EML4-ALK mutations expressing Ba/F3 cells.

Phospho-ALK in each EML4-ALK mutations expressing Ba/F3 cells were evaluated by western blotting. Cells were treated with the indicated concentrations of lorlatinib for 3 h (n=2).

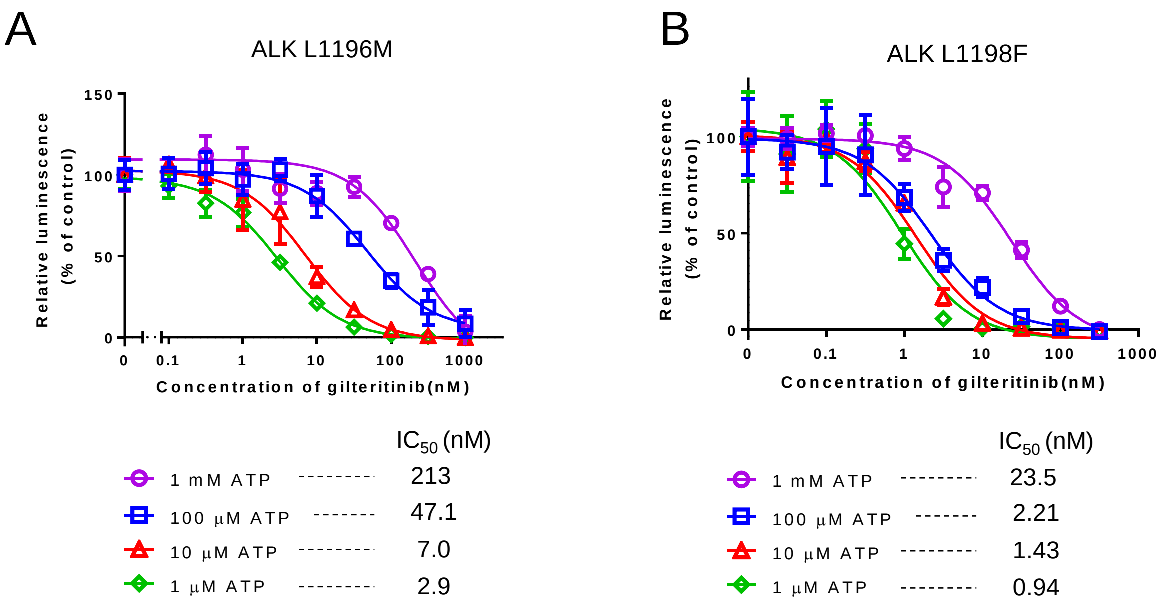

Supplementary Figure 25. The evaluation of the inhibitory activity of gilteritinib in the in vitro kinase assay .

(A,B) The evaluation of the inhibitory activity of gilteritinib to L1196M (A), L1198F (B) mutated ALK in the in vitro kinase assay using the ADP-Glo assay kit. It showed a dose-dependent inhibition in ALK activity with gilteritinib according to the increase of ATP concentration. N=3 independent samples examined over 3 independent experiments and representative experiment data are presented as mean values +/- SD.

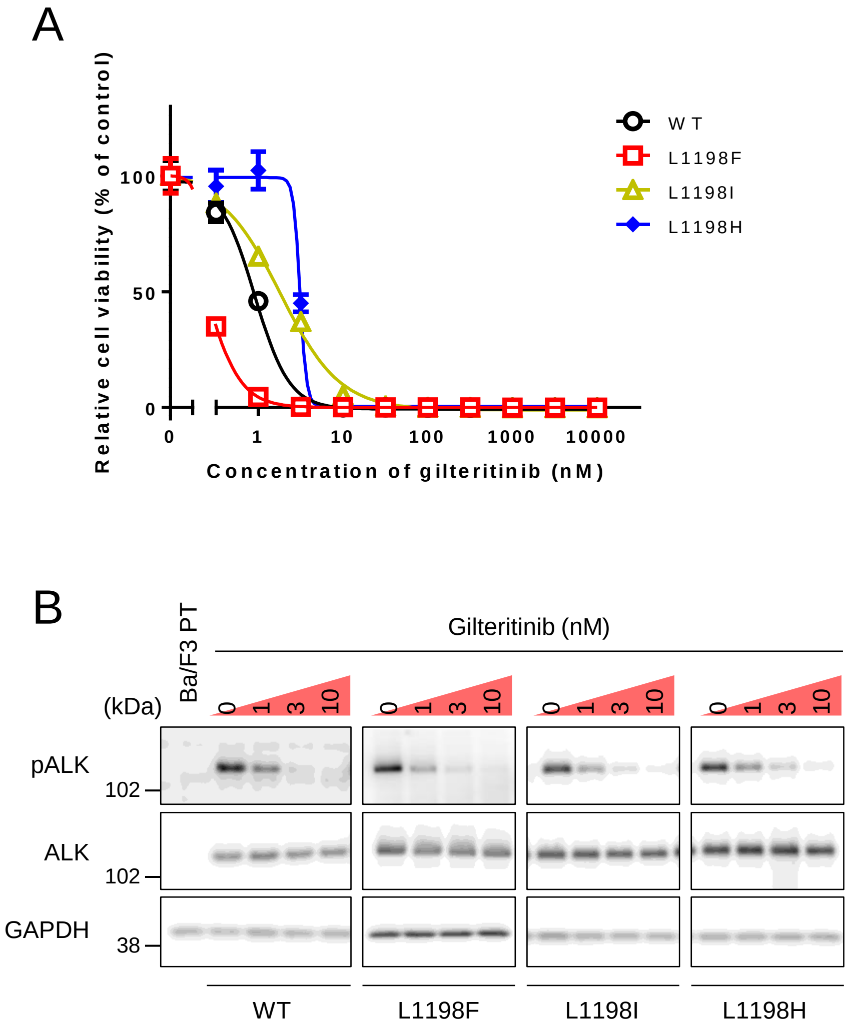

Supplementary Figure 26. ALK L1198F mutant is more sensitive to gilteritinib.

(A) The activity of the indicated inhibitors in Ba/F3 cells expressing EML4-ALK wild-type and indicated L1198X mutants. Cells were treated with the gilteritinib for 72 h, and viability was analyzed using the CellTiter-Glo assay. N=3 independent samples were examined and data are presented as mean values +/- SD. (B) Phospho-ALK in each EML4-ALK mutations expressing Ba/F3 cells were evaluated by western blotting. Cells were treated with the indicated concentrations of gilteritinib for 3 h (n=2).

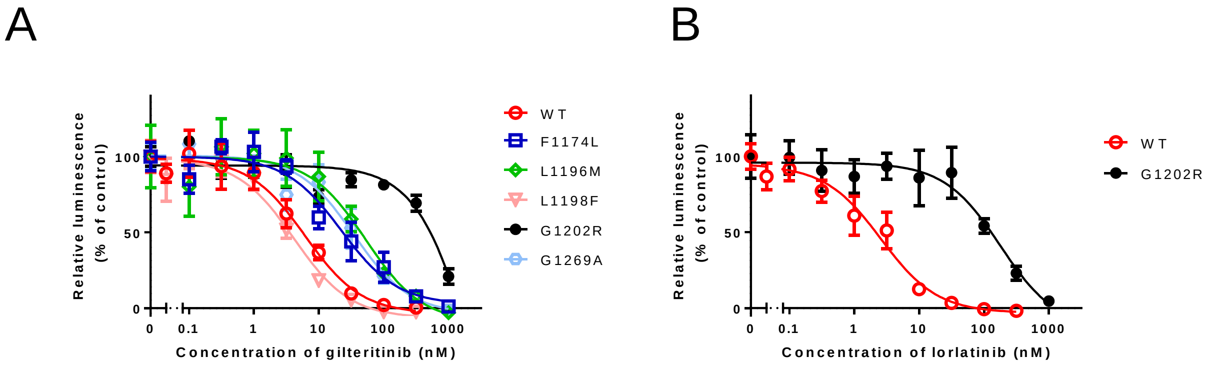

Supplementary Figure 27. The evaluation of the inhibitory activity in the in vitro kinase assay .

(A,B) The evaluation of the inhibitory activity of gilteritinib (A) or lorlatinib (B) to indicated mutated ALK in the in vitro kinase assay using the ADP-Glo assay kit. At an ATP concentration of 100 μM, ALK G1202R mutant showed resistant against gilteritinib compared with other mutants and G1202R against lorlatinib. N=3 independent samples examined over 2 independent experiments and representative experiment data are presented as mean values +/- SD.

Supplementary Figure 28: Original immunoblots for indicated figures

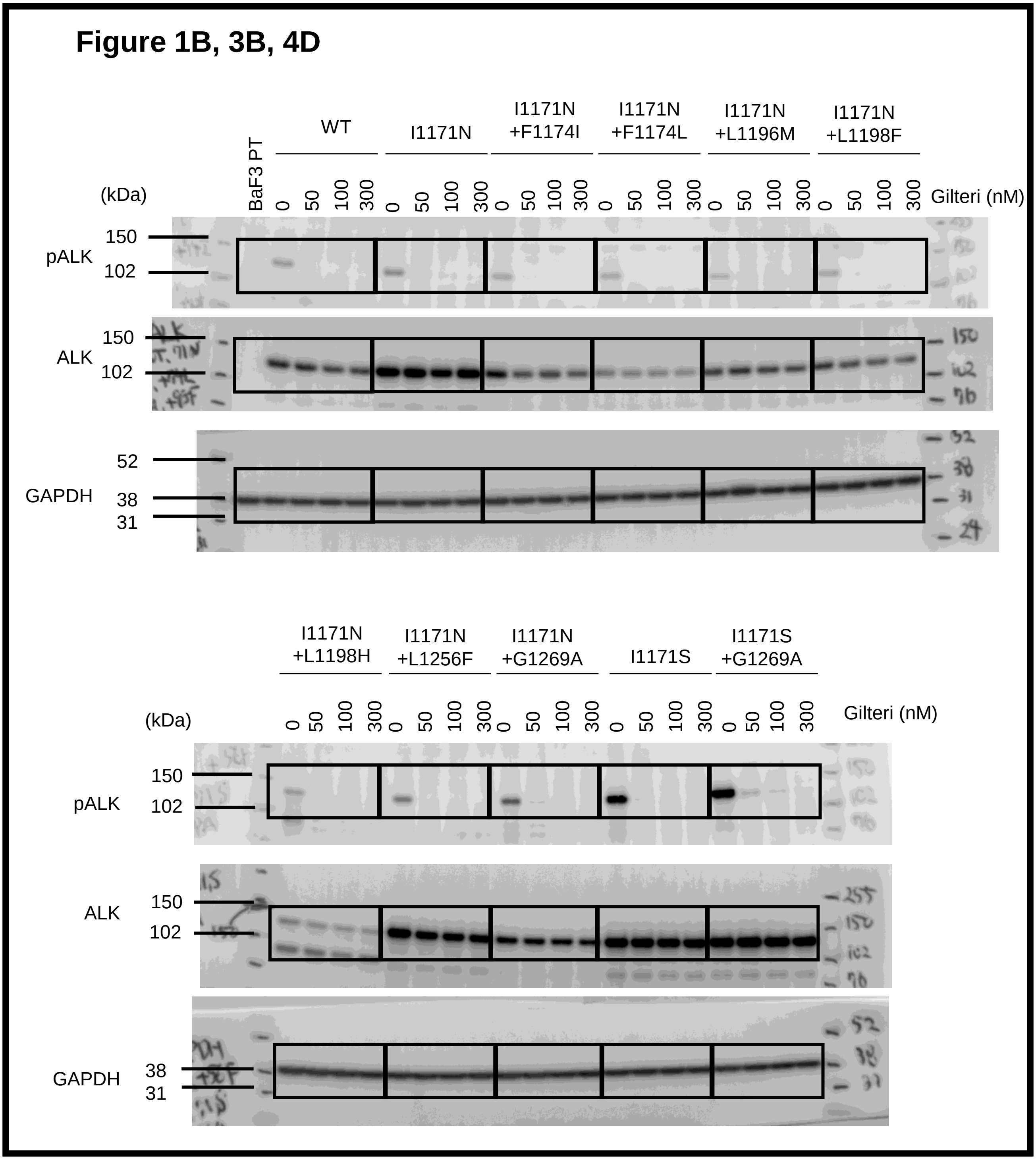

Supplementary Figure 28: Original immunoblots for indicated figures

Figure 1C, S2

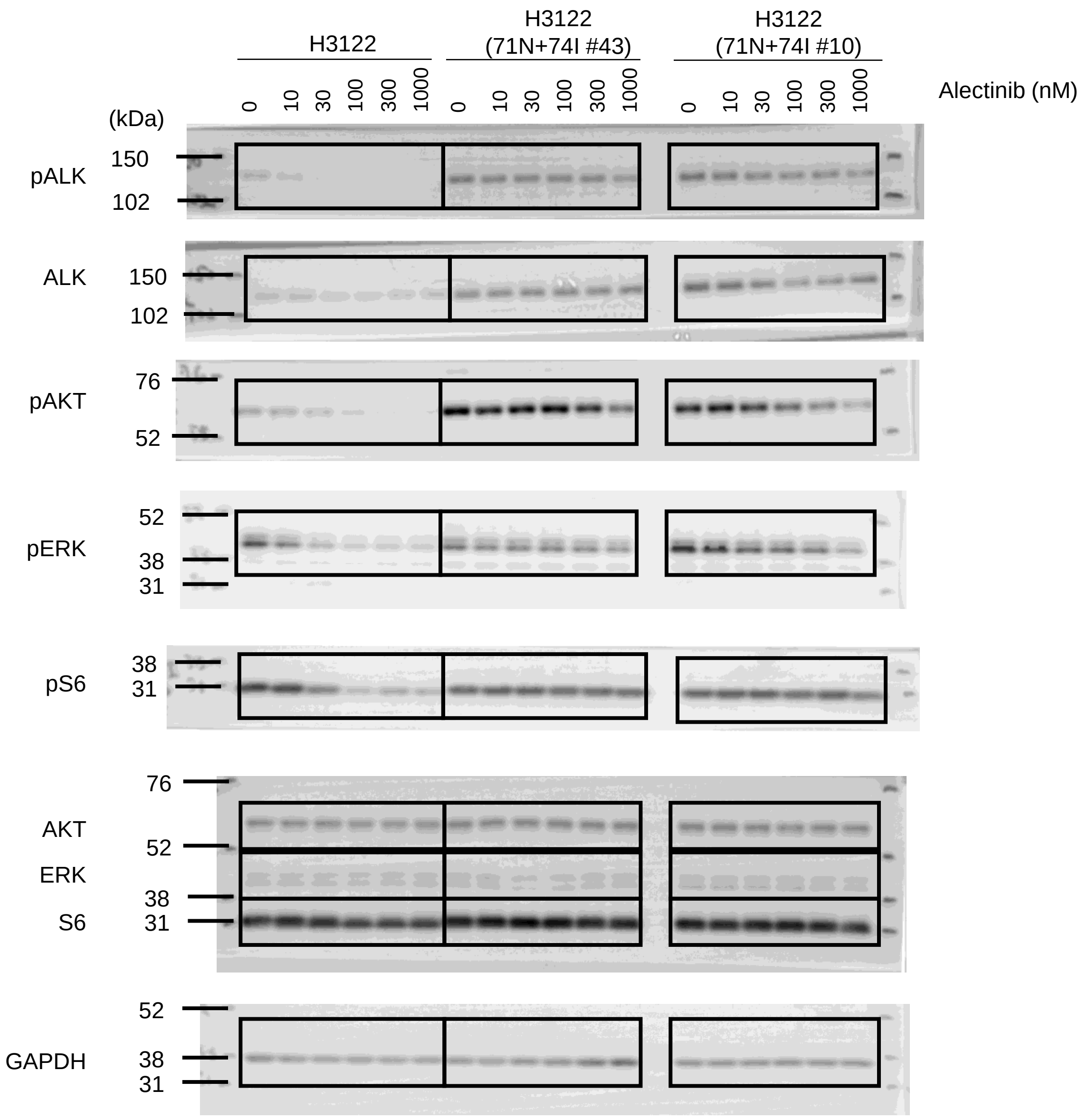

Supplementary Figure 28: Original immunoblots for indicated figures

Figure 1C, S2

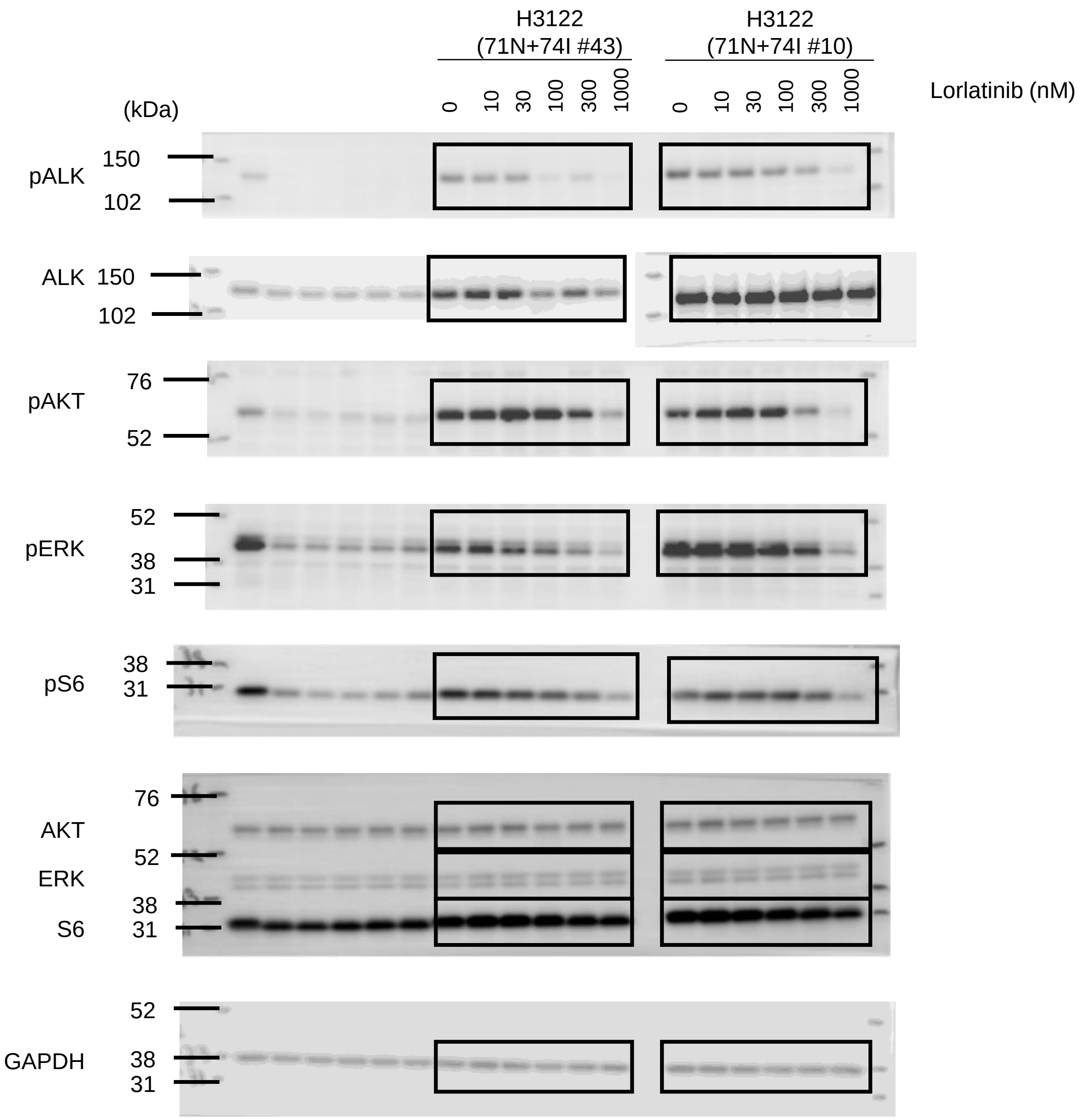

Supplementary Figure 28: Original immunoblots for indicated figures

Figure 1C, S2

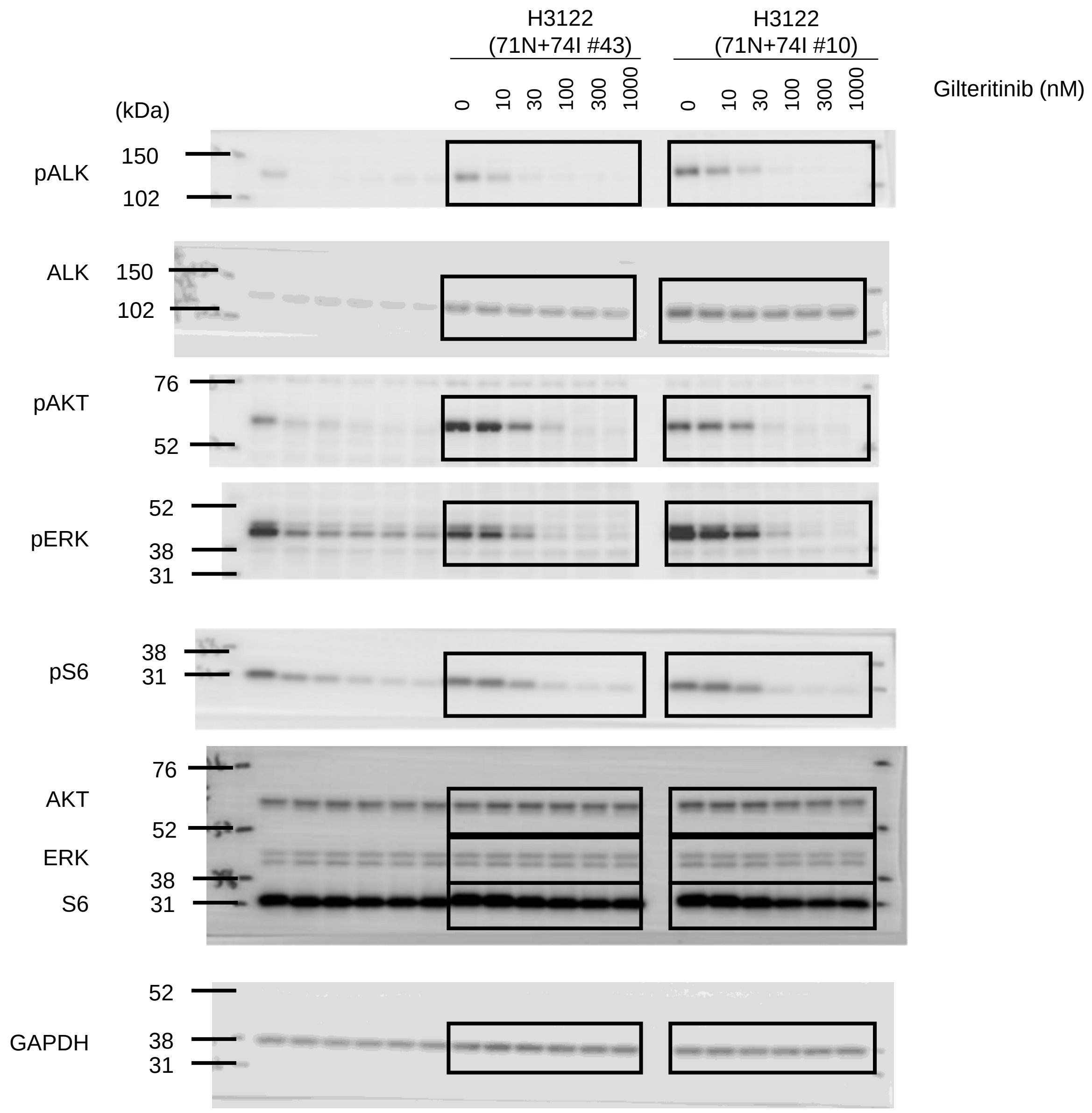

Supplementary Figure 28: Original immunoblots for indicated figures

Figure 1C, S2

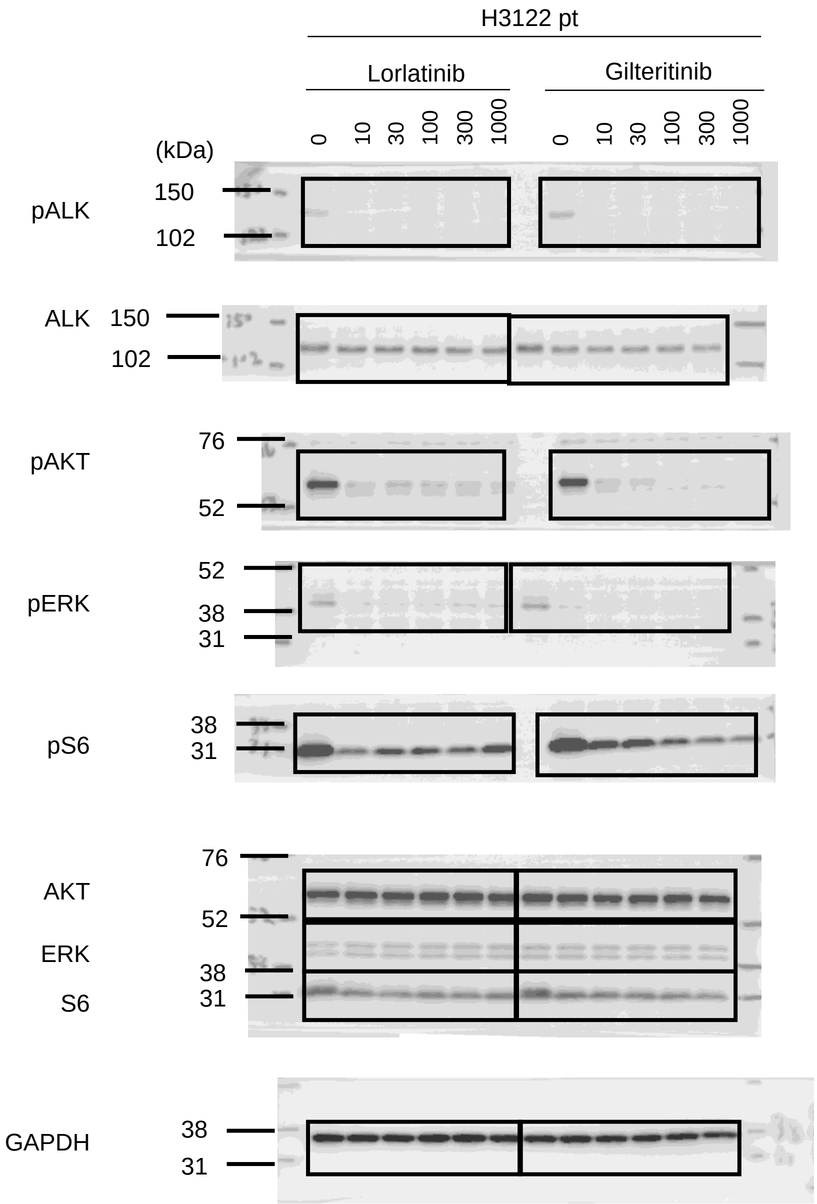

Supplementary Figure 28: Original immunoblots for indicated figures

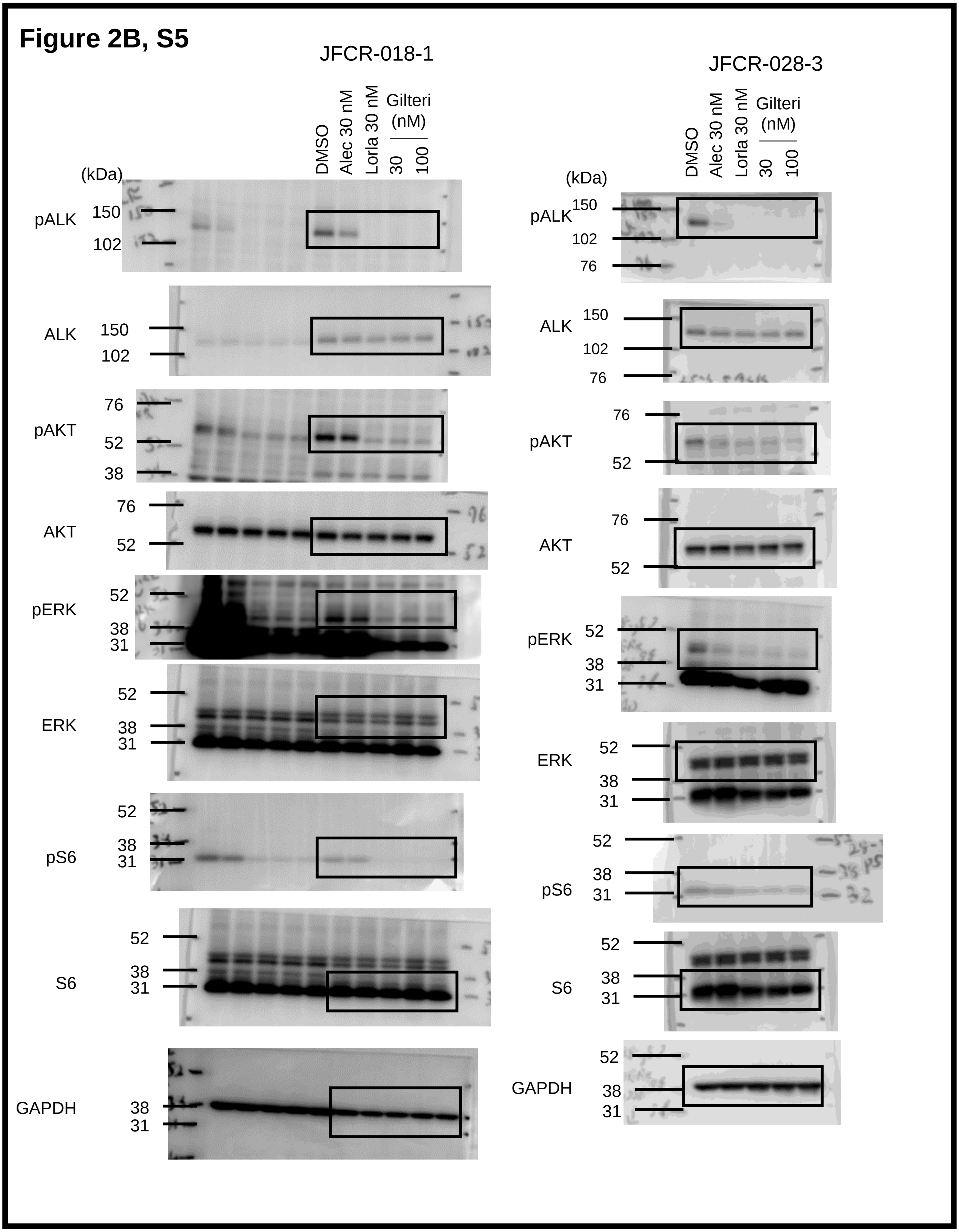

Supplementary Figure 28: Original immunoblots for indicated figures

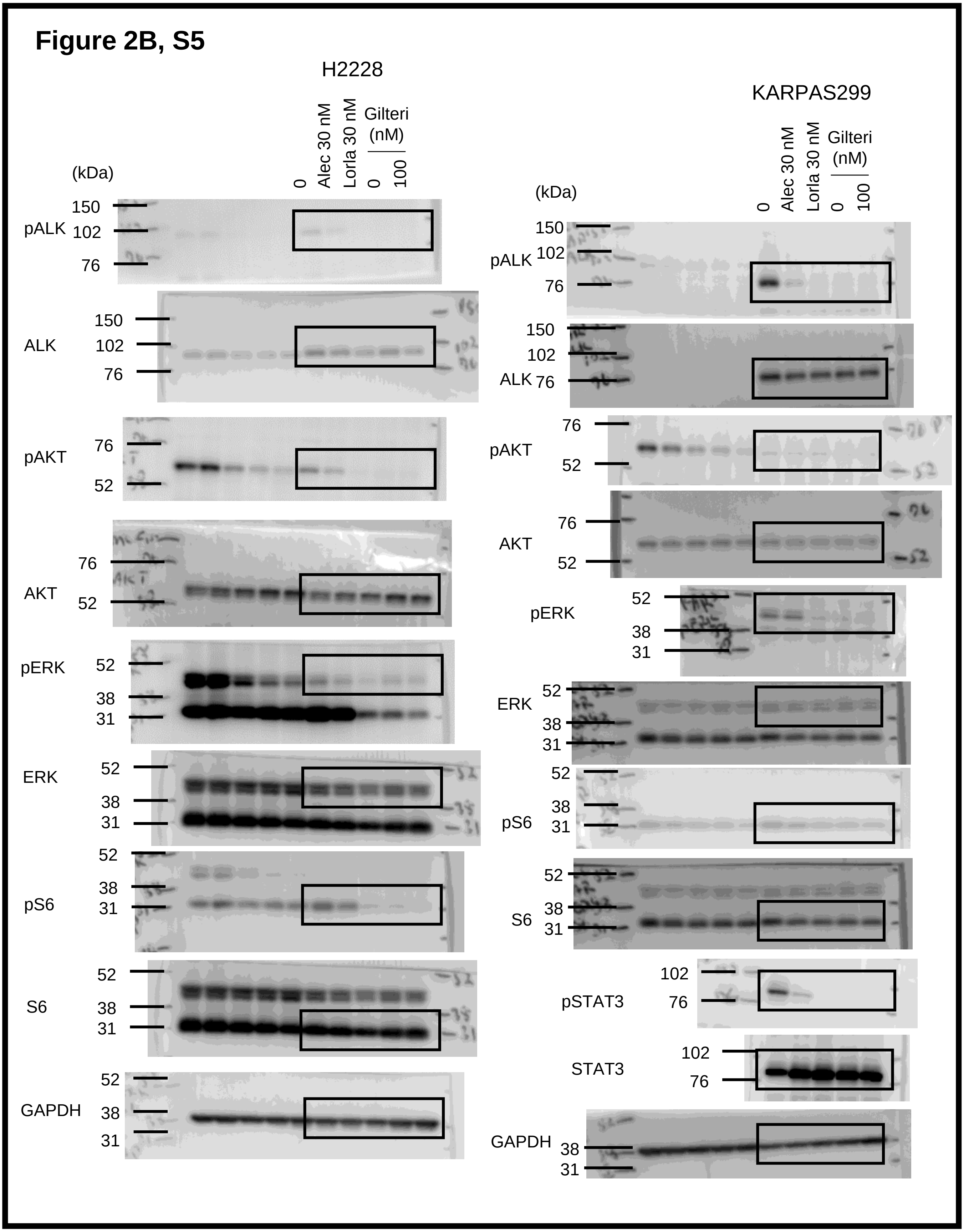

Supplementary Figure 28: Original immunoblots for indicated figures

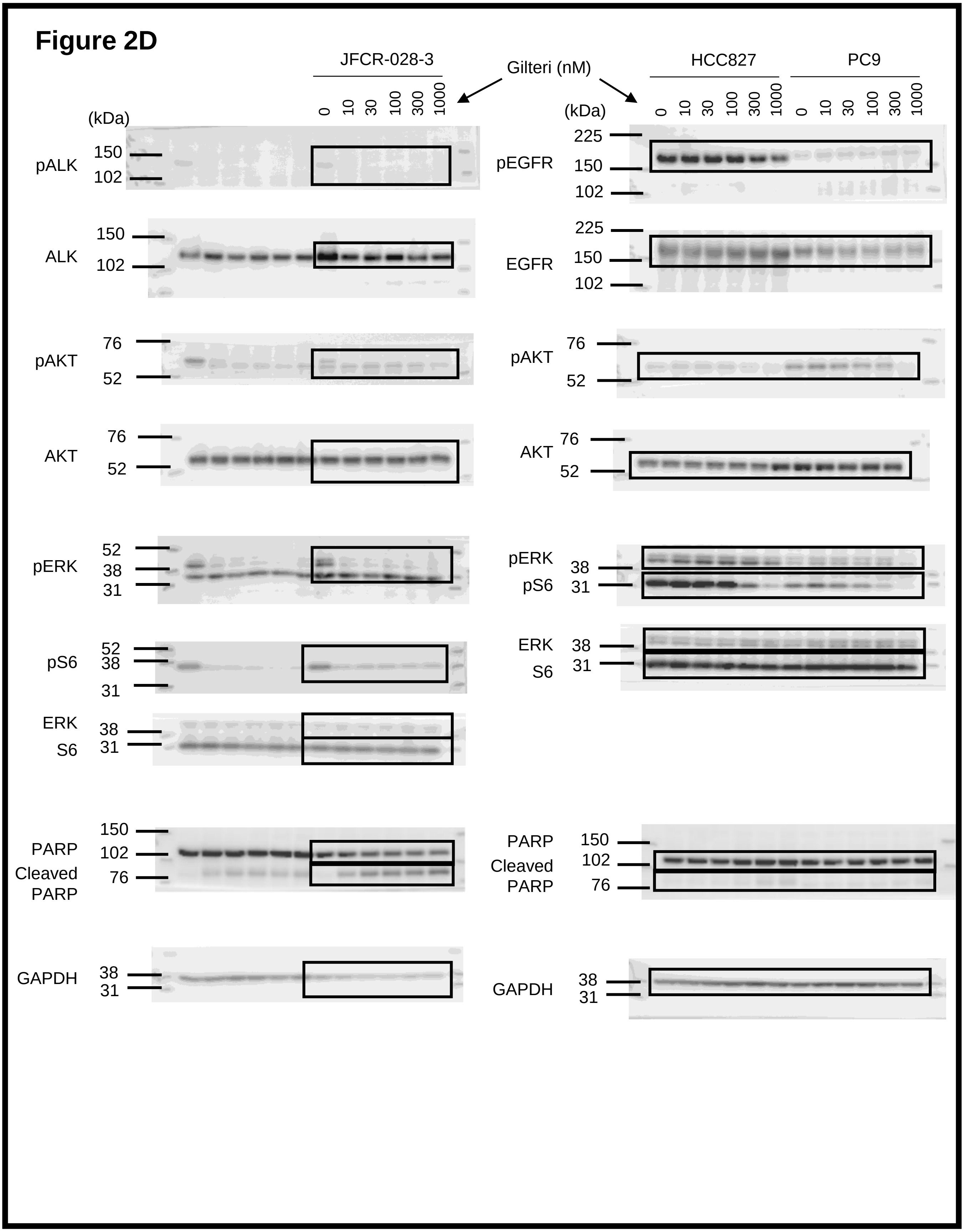

Supplementary Figure 28: Original immunoblots for indicated figures

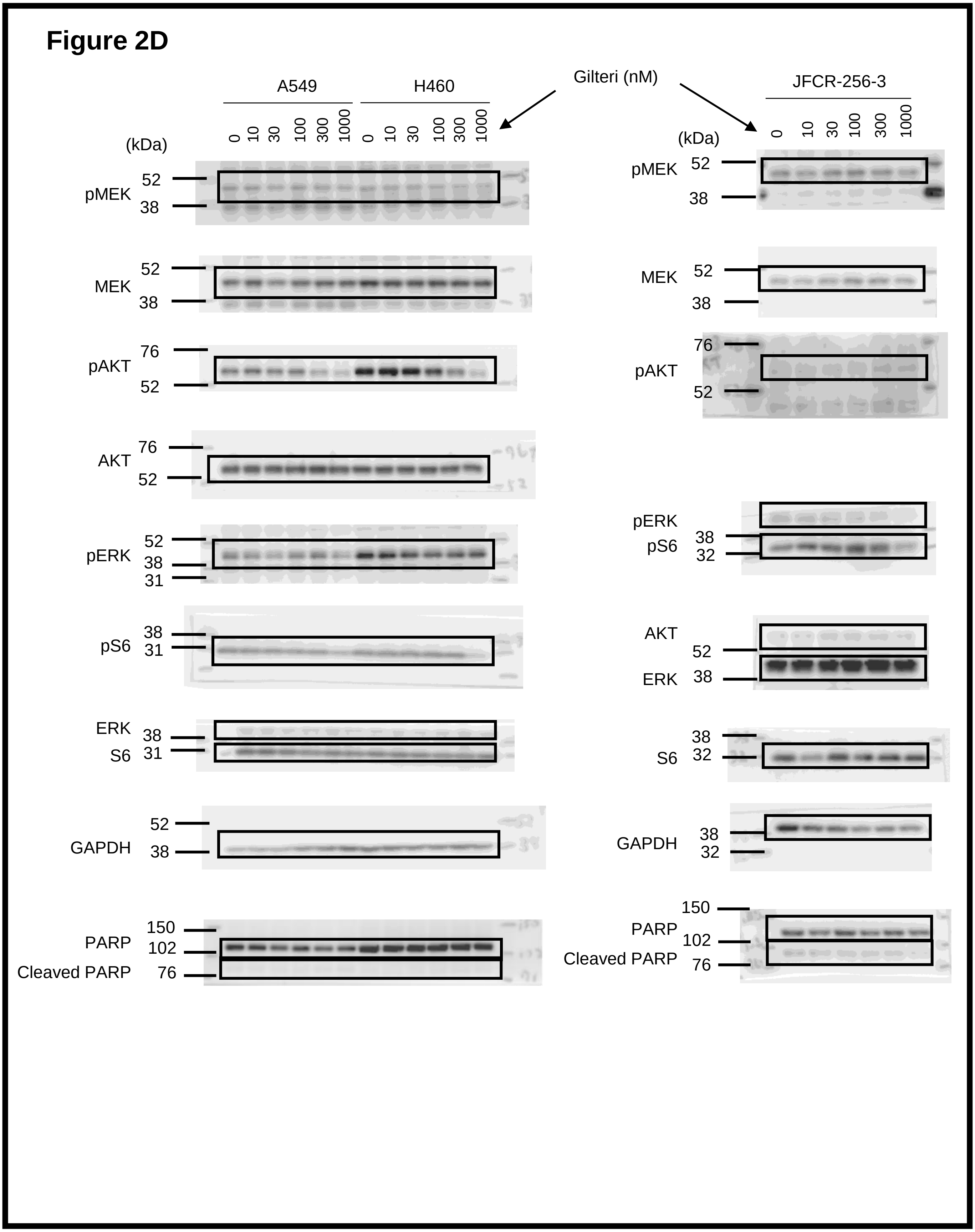

Supplementary Figure 28: Original immunoblots for indicated figures

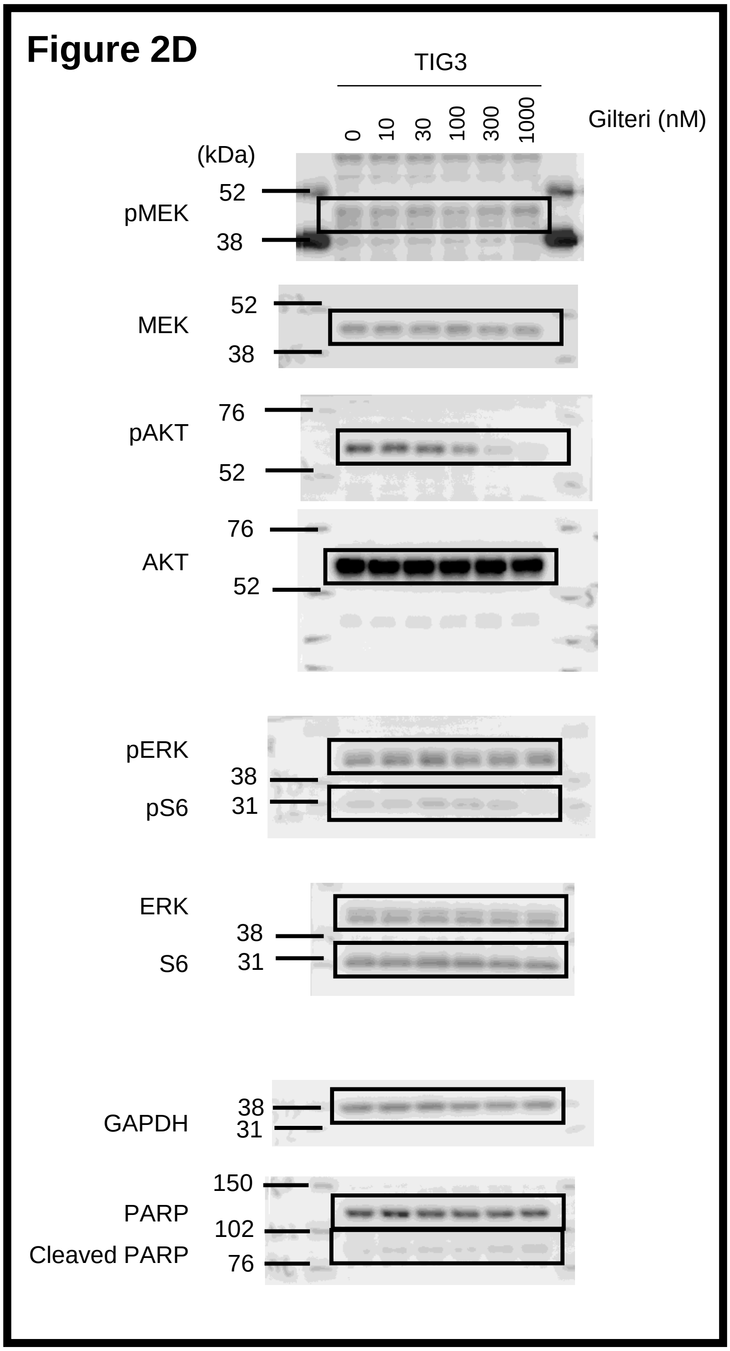

Supplementary Figure 28: Original immunoblots for indicated figures

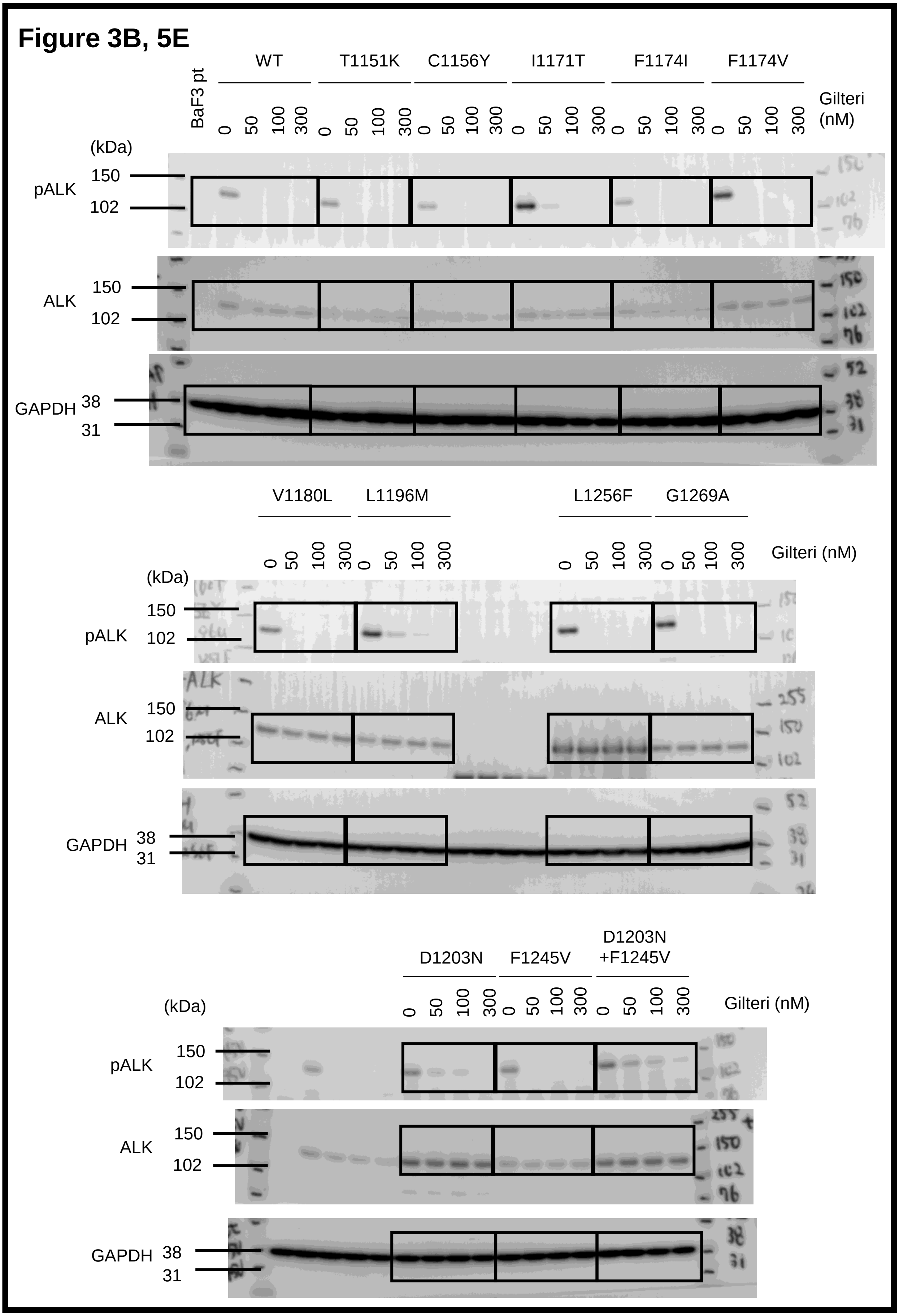

Supplementary Figure 28: Original immunoblots for indicated figures

Figure 3B, 5E, S13A

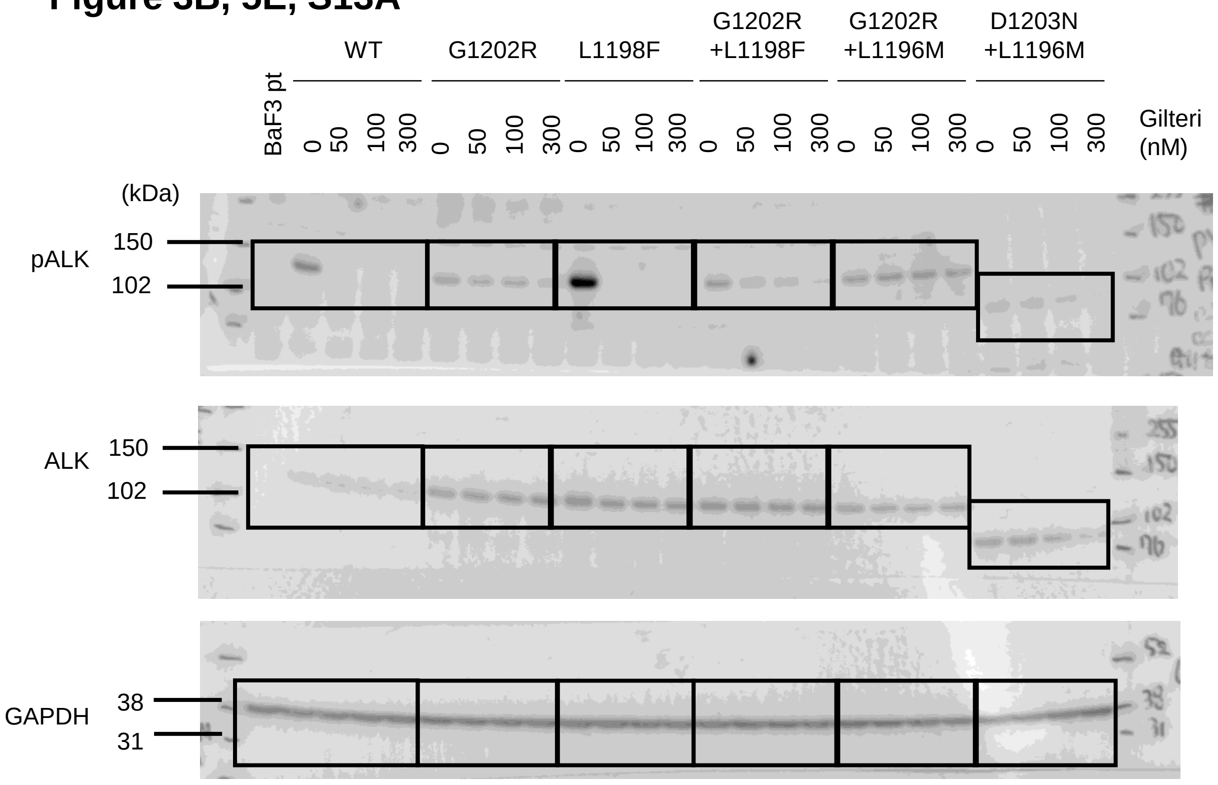

Supplementary Figure 28: Original immunoblots for indicated figures

Figure 3B, S23, S24

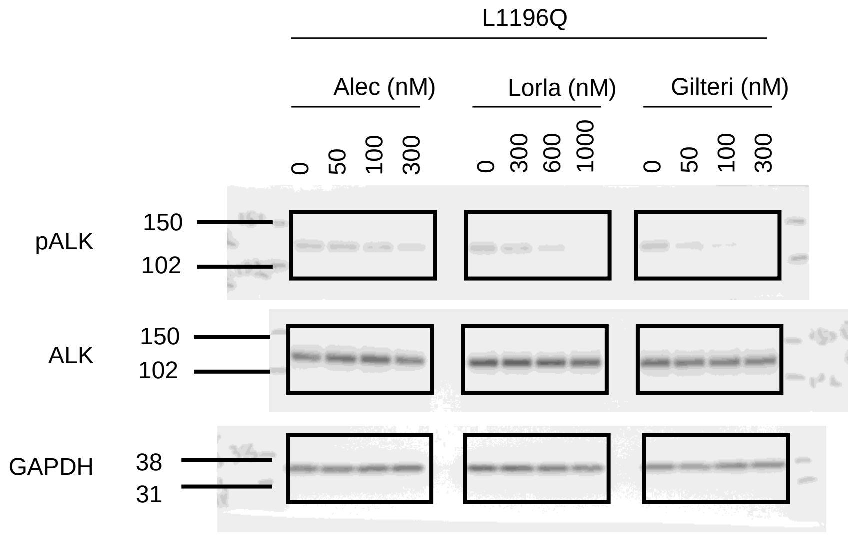

Supplementary Figure 28: Original immunoblots for indicated figures

Figure 3C

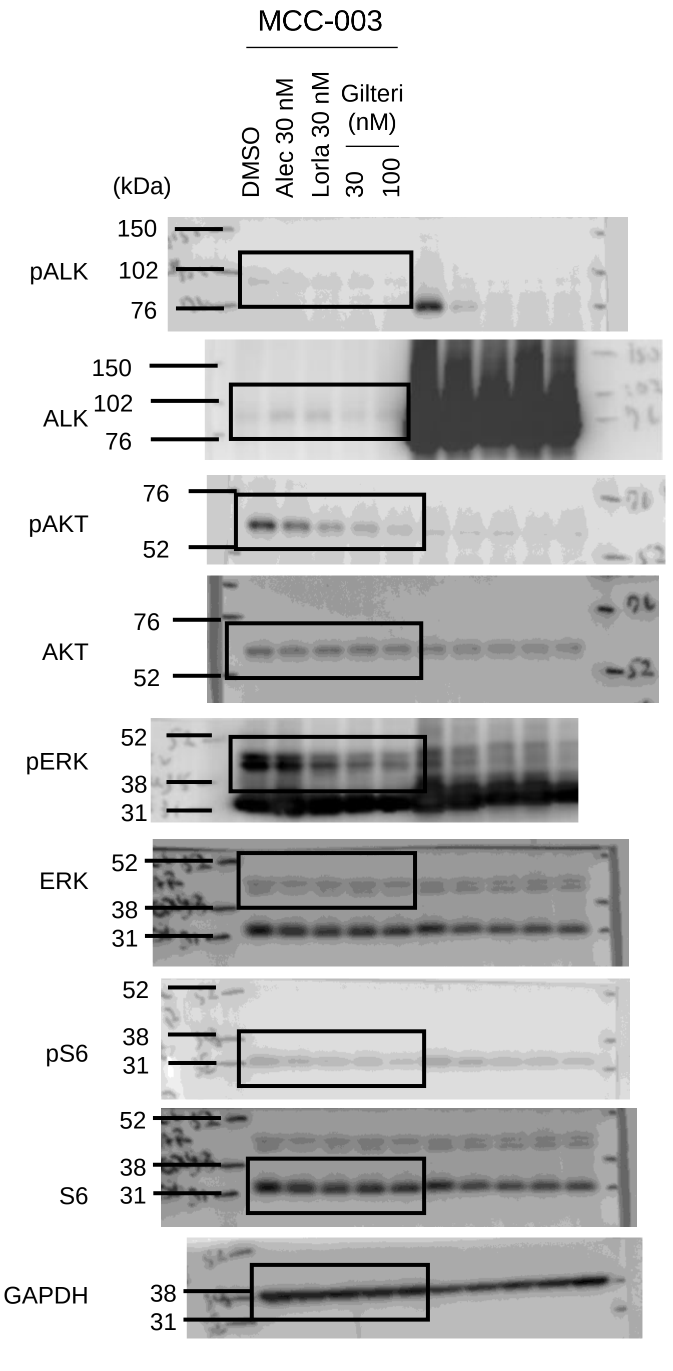

Figure 3F

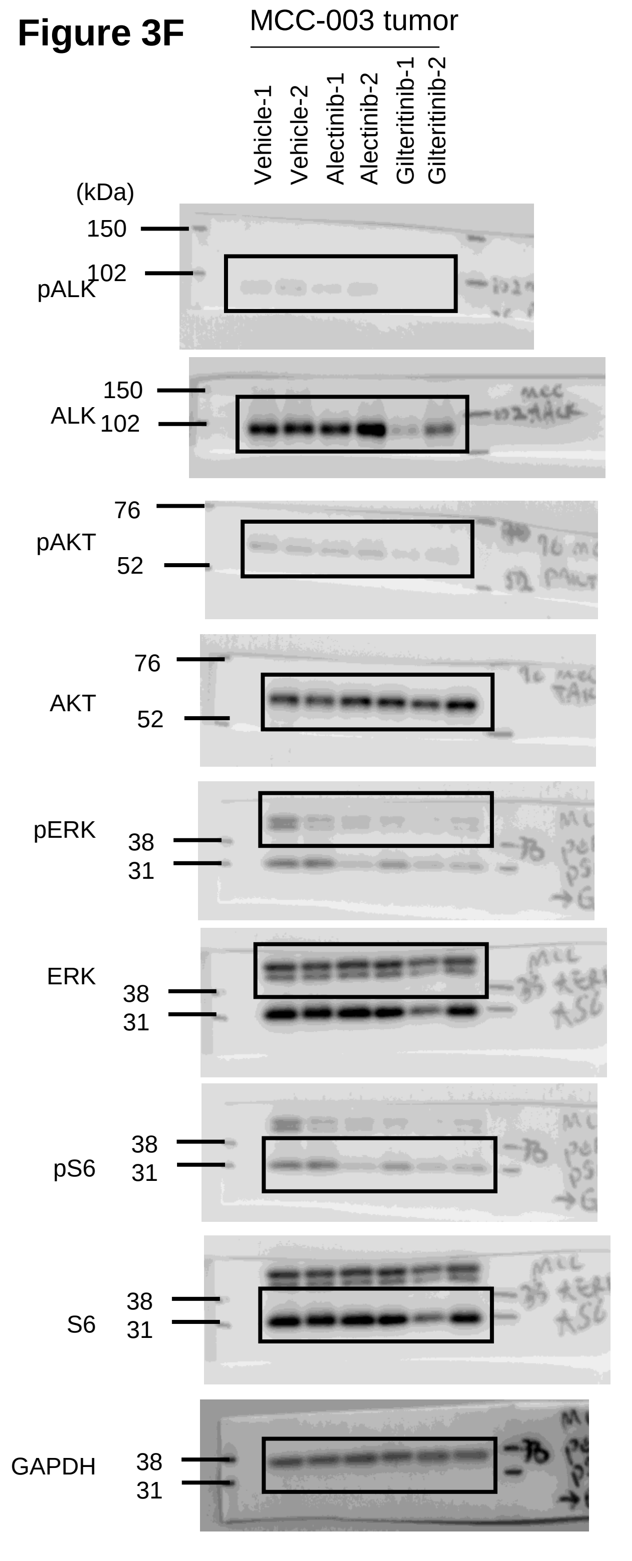

Supplementary Figure 28: Original immunoblots for indicated figures

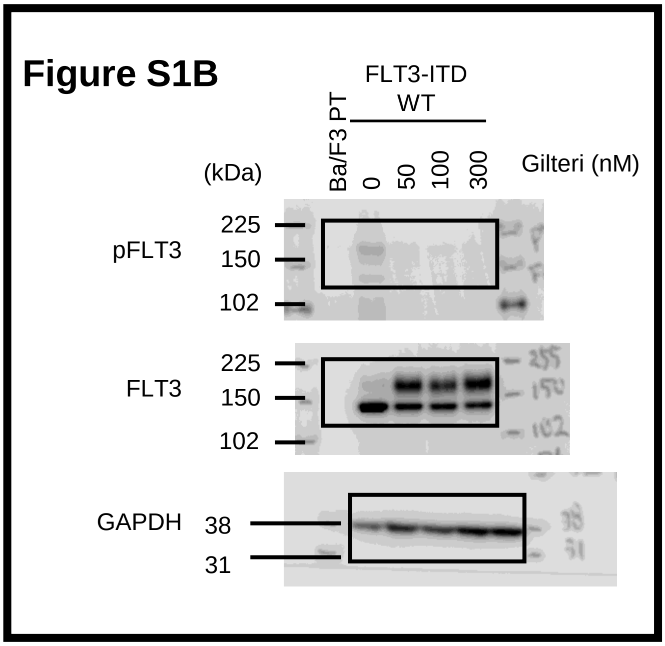

Supplementary Figure 28: Original immunoblots for indicated figures

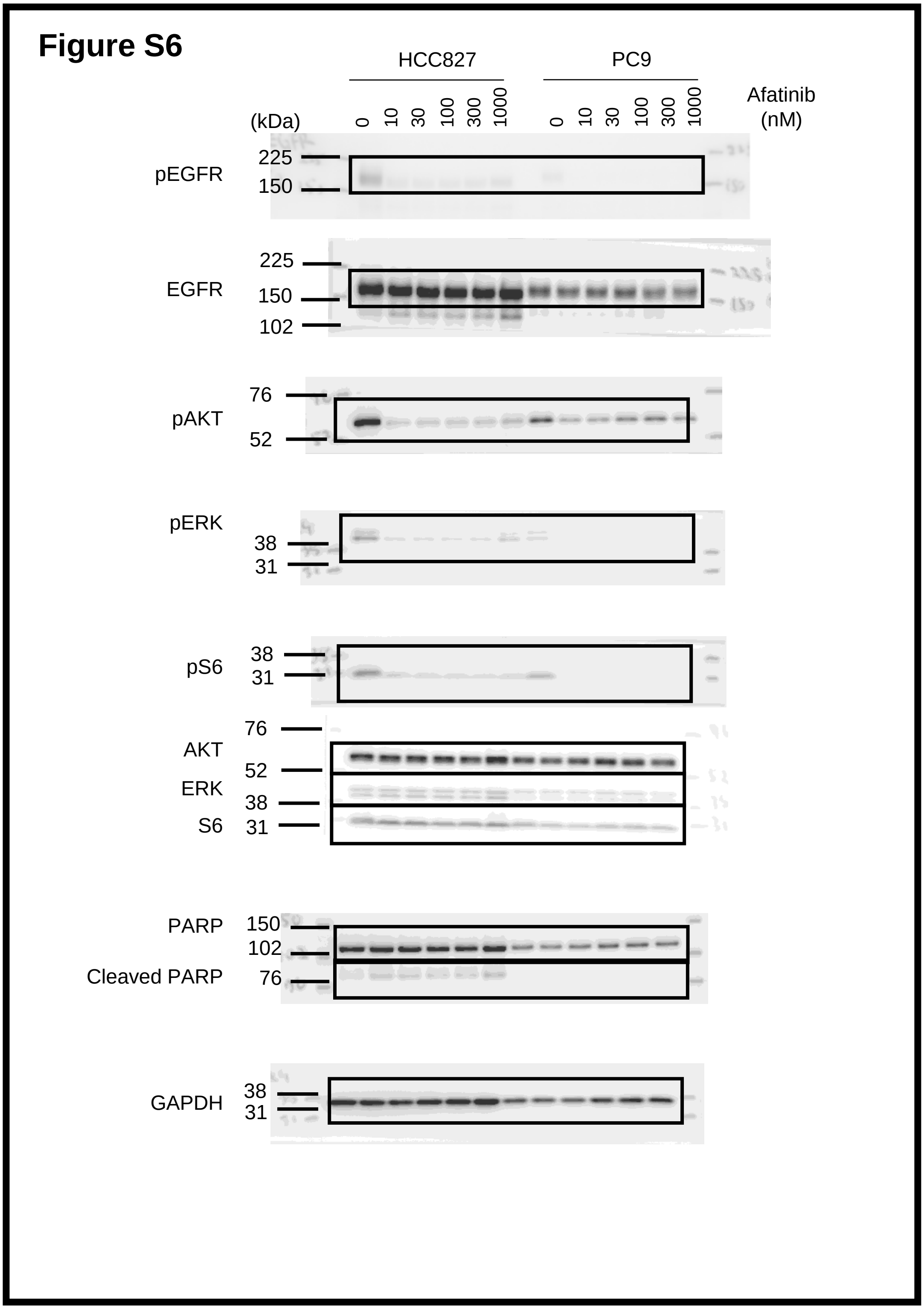

Supplementary Figure 28: Original immunoblots for indicated figures

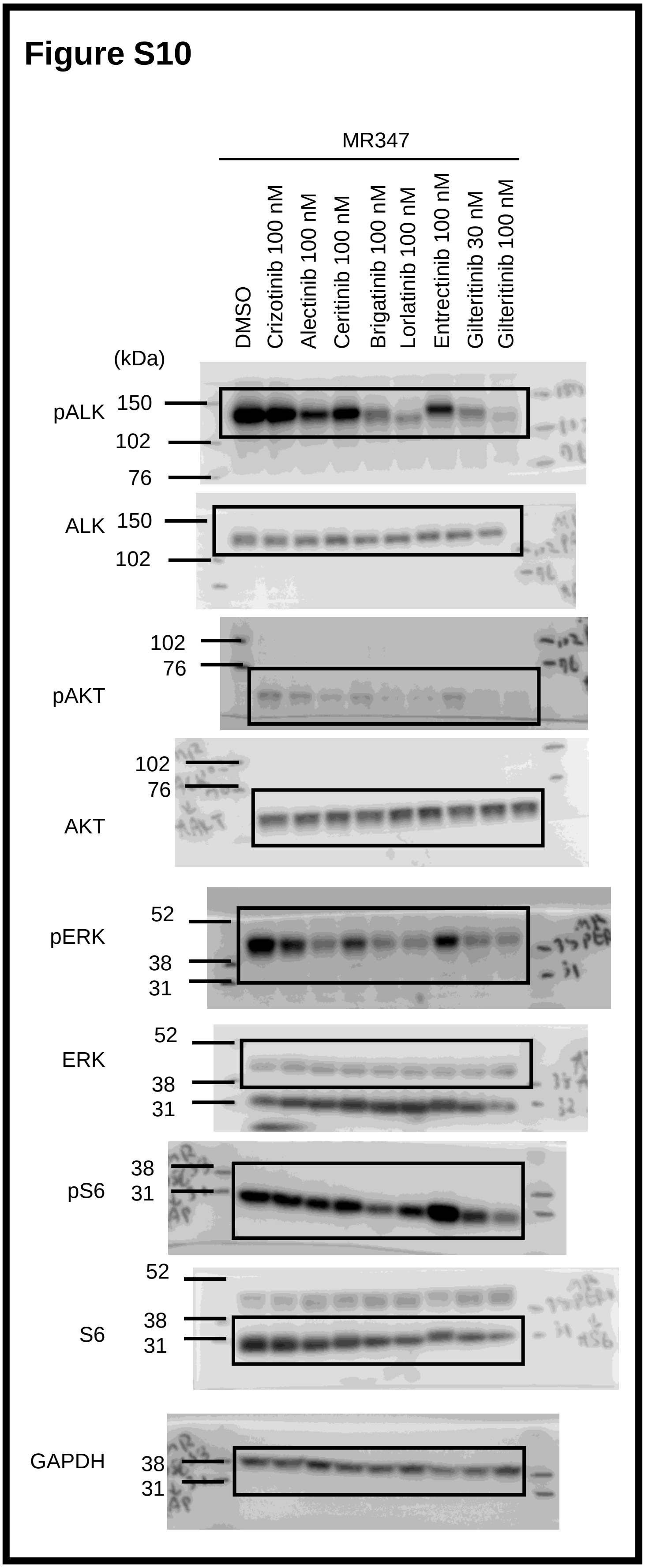

Supplementary Figure 28: Original immunoblots for indicated figures

Figure S13B

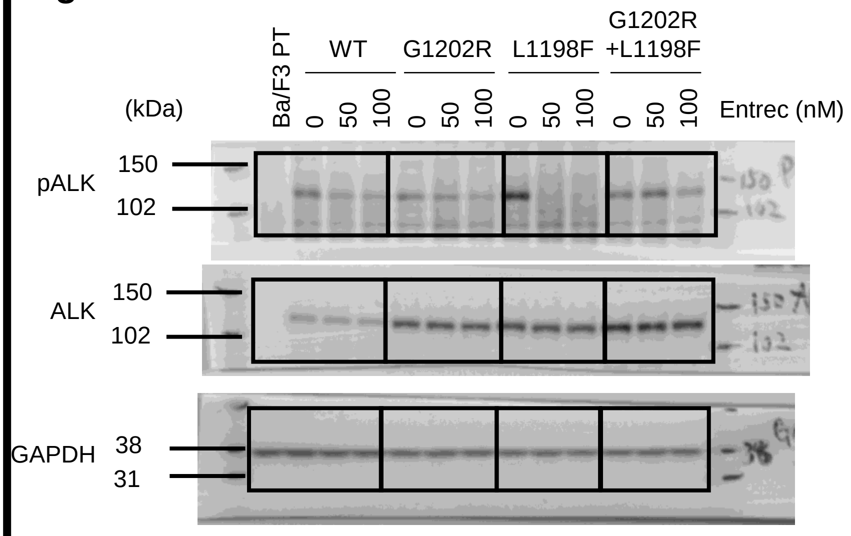

Supplementary Figure 28: Original immunoblots for indicated figures

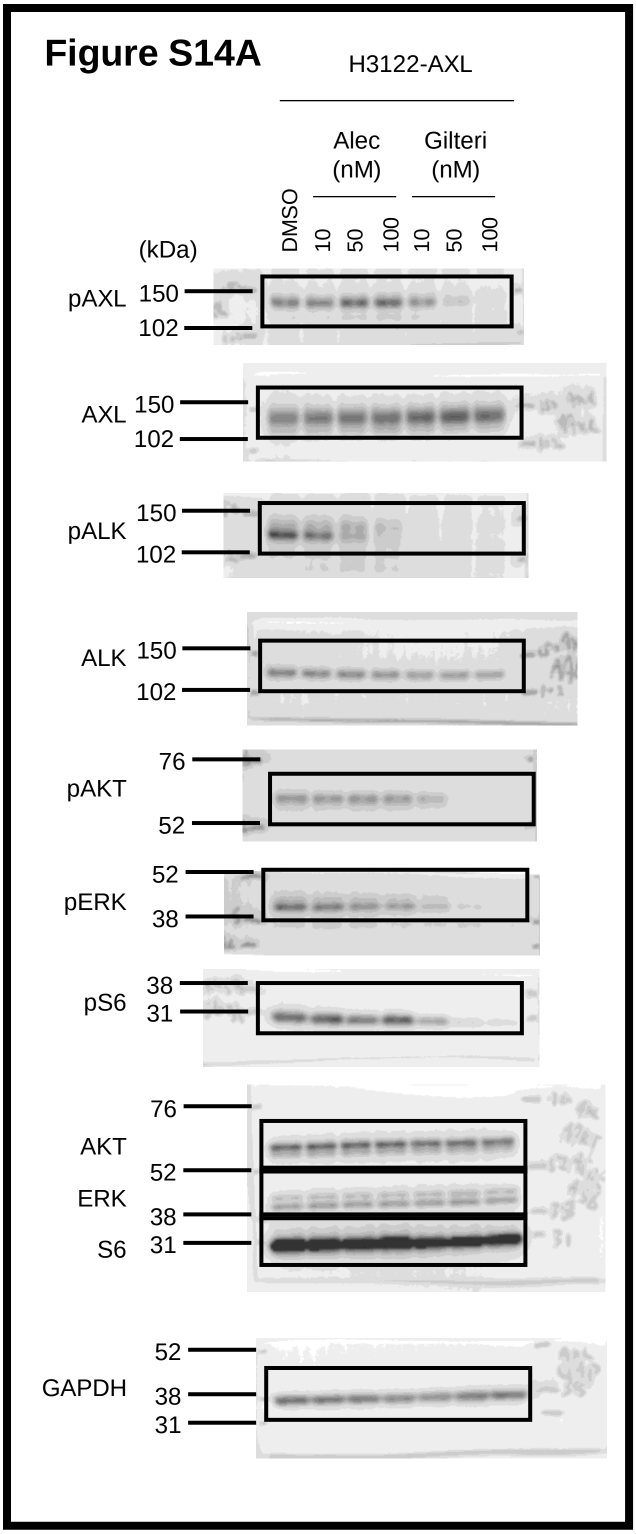

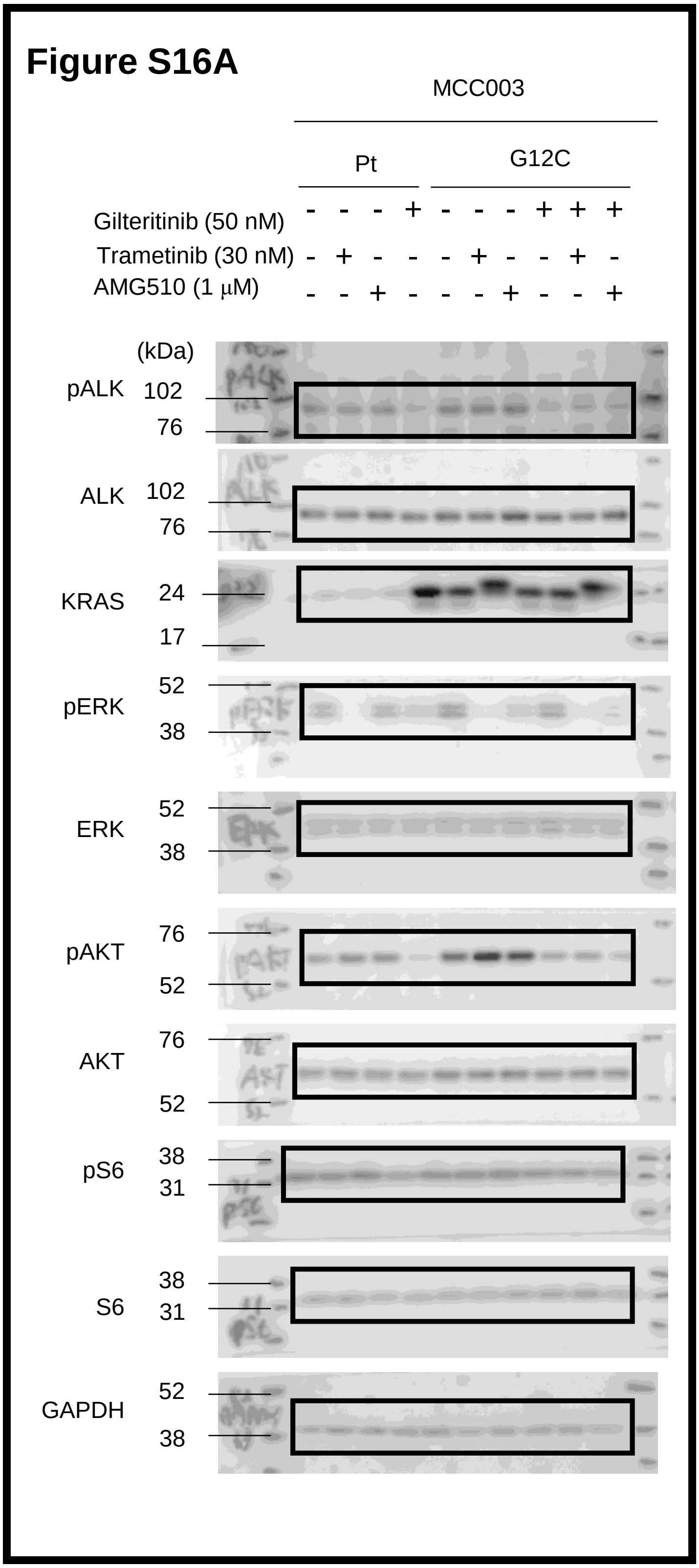

Supplementary Figure 28: Original immunoblots for indicated figures

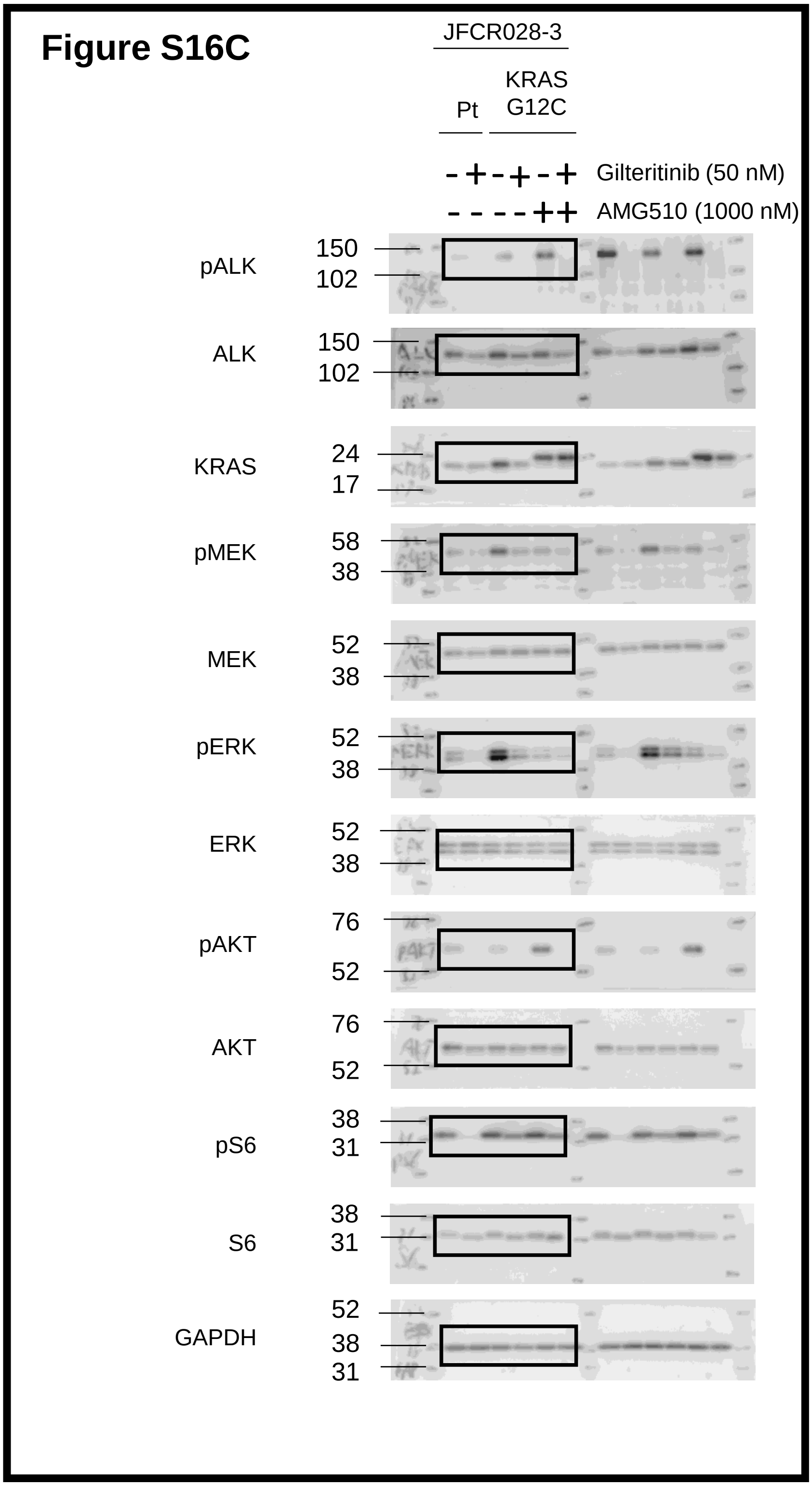

Supplementary Figure 28: Original immunoblots for indicated figures

Figure S18B

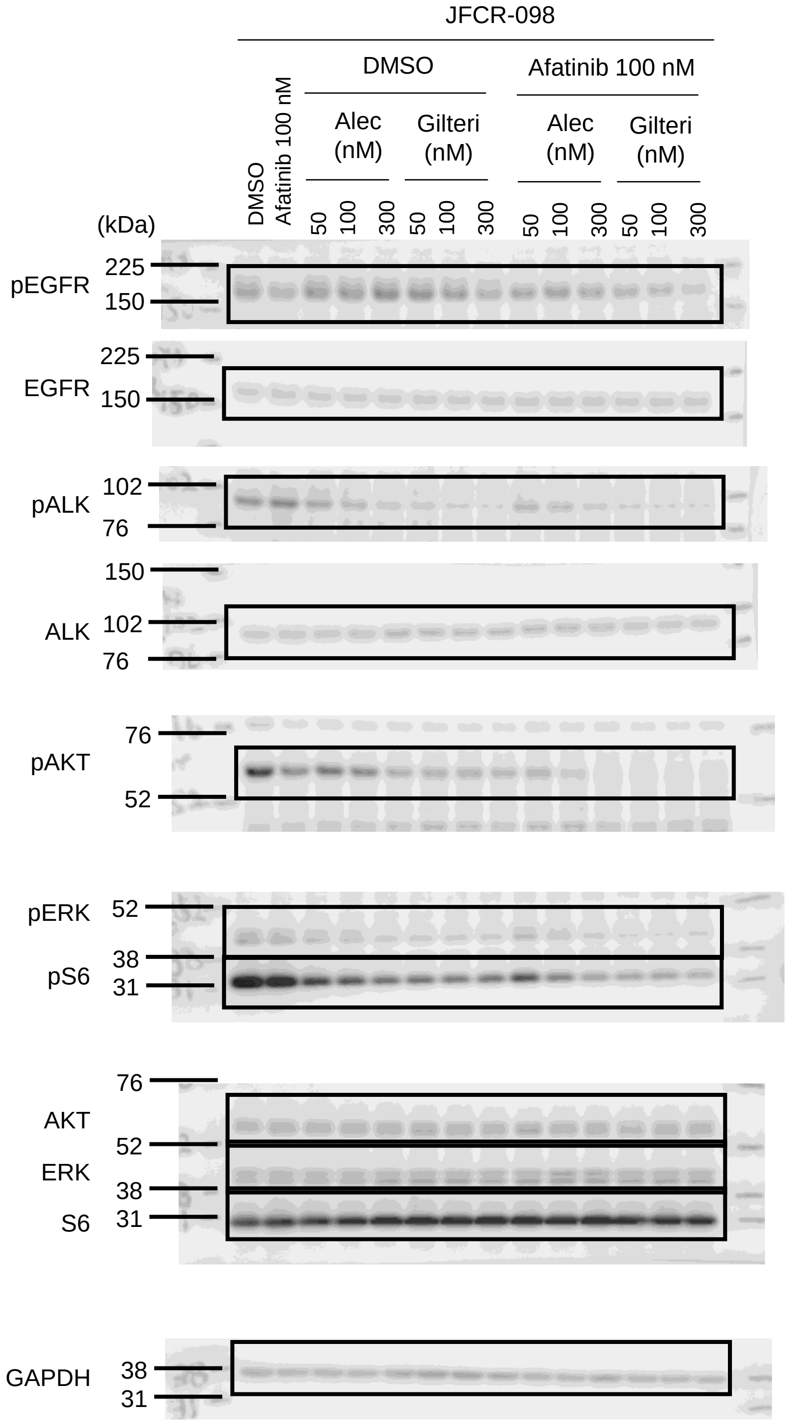

Figure S19C

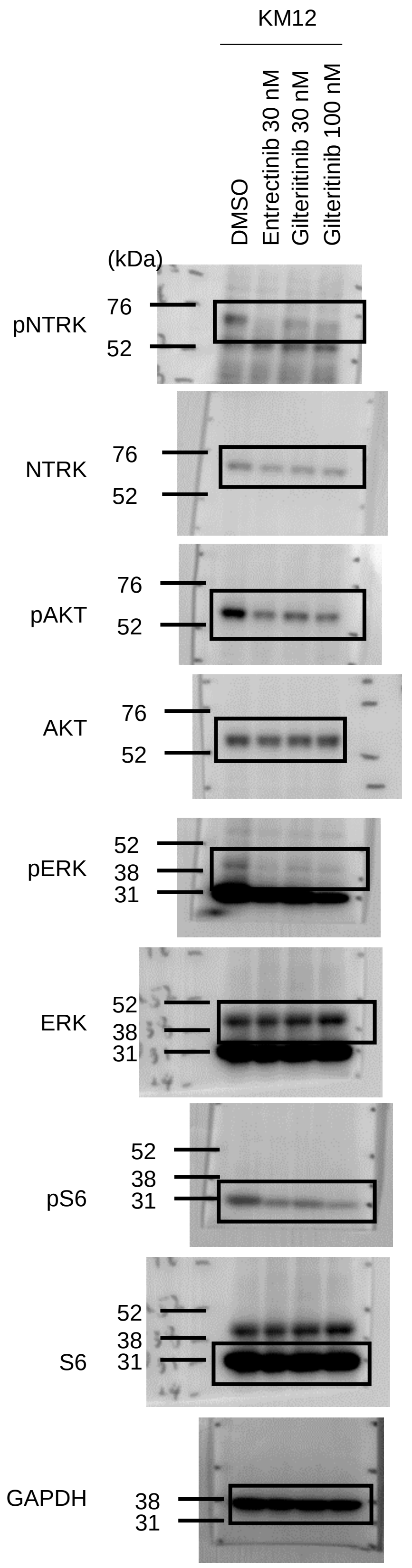

Supplementary Figure 28: Original immunoblots for indicated figures

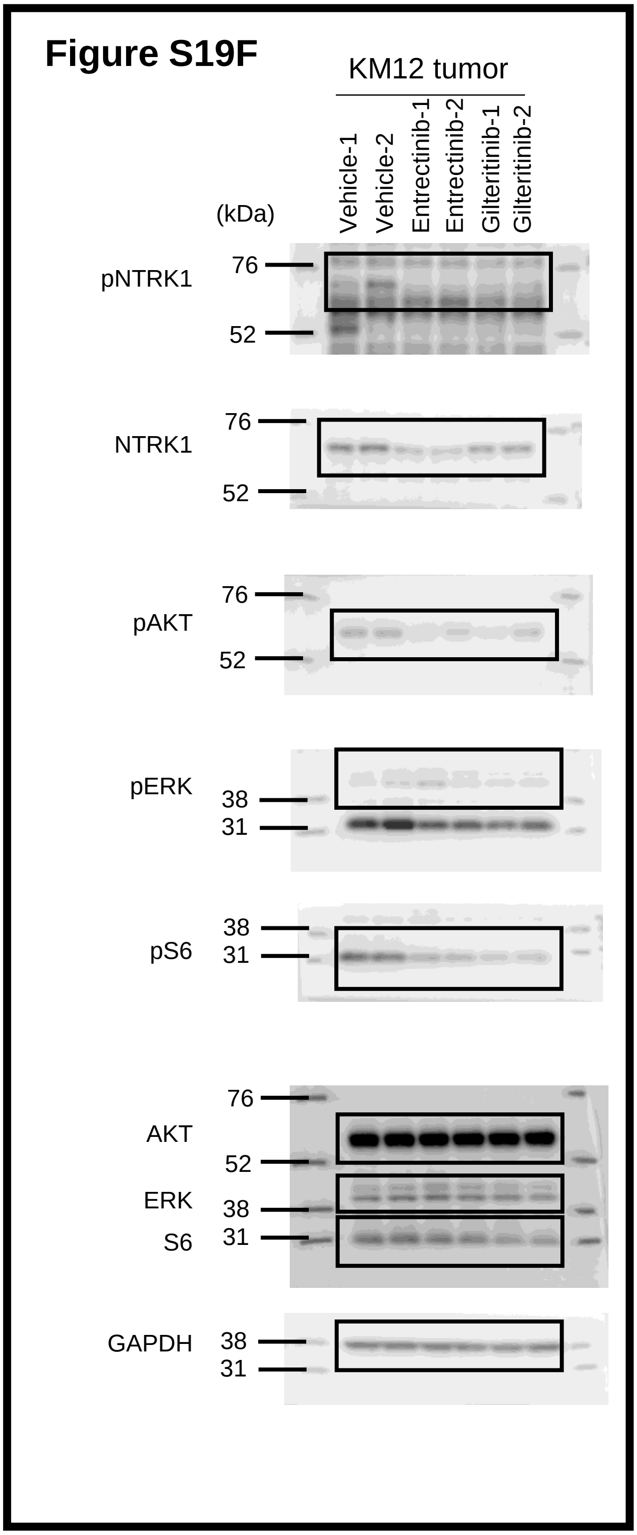

Supplementary Figure 28: Original immunoblots for indicated figures

Figure S20D

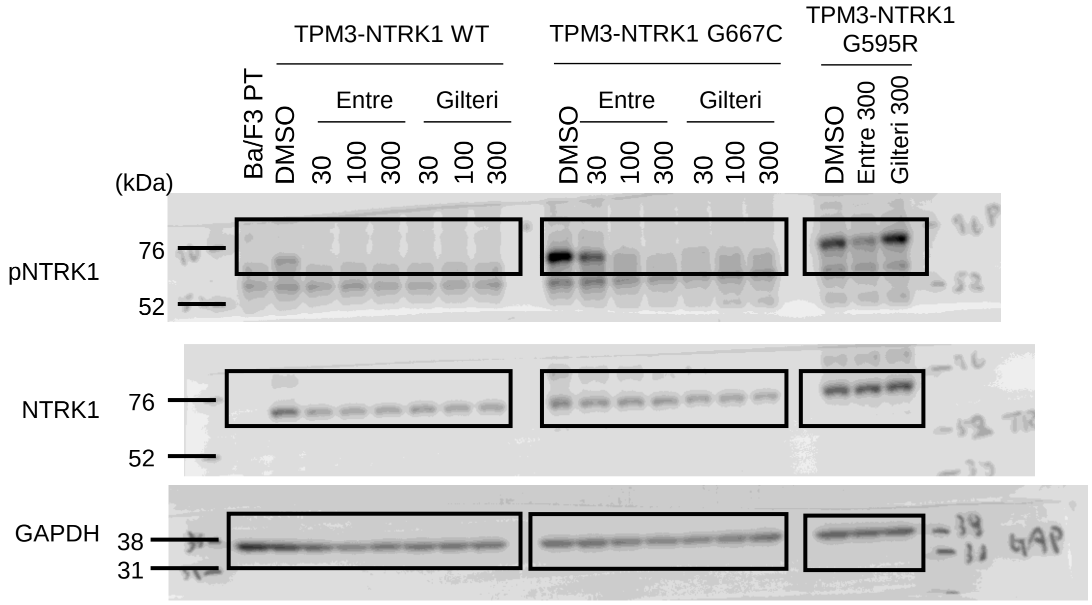

Supplementary Figure 28: Original immunoblots for indicated figures

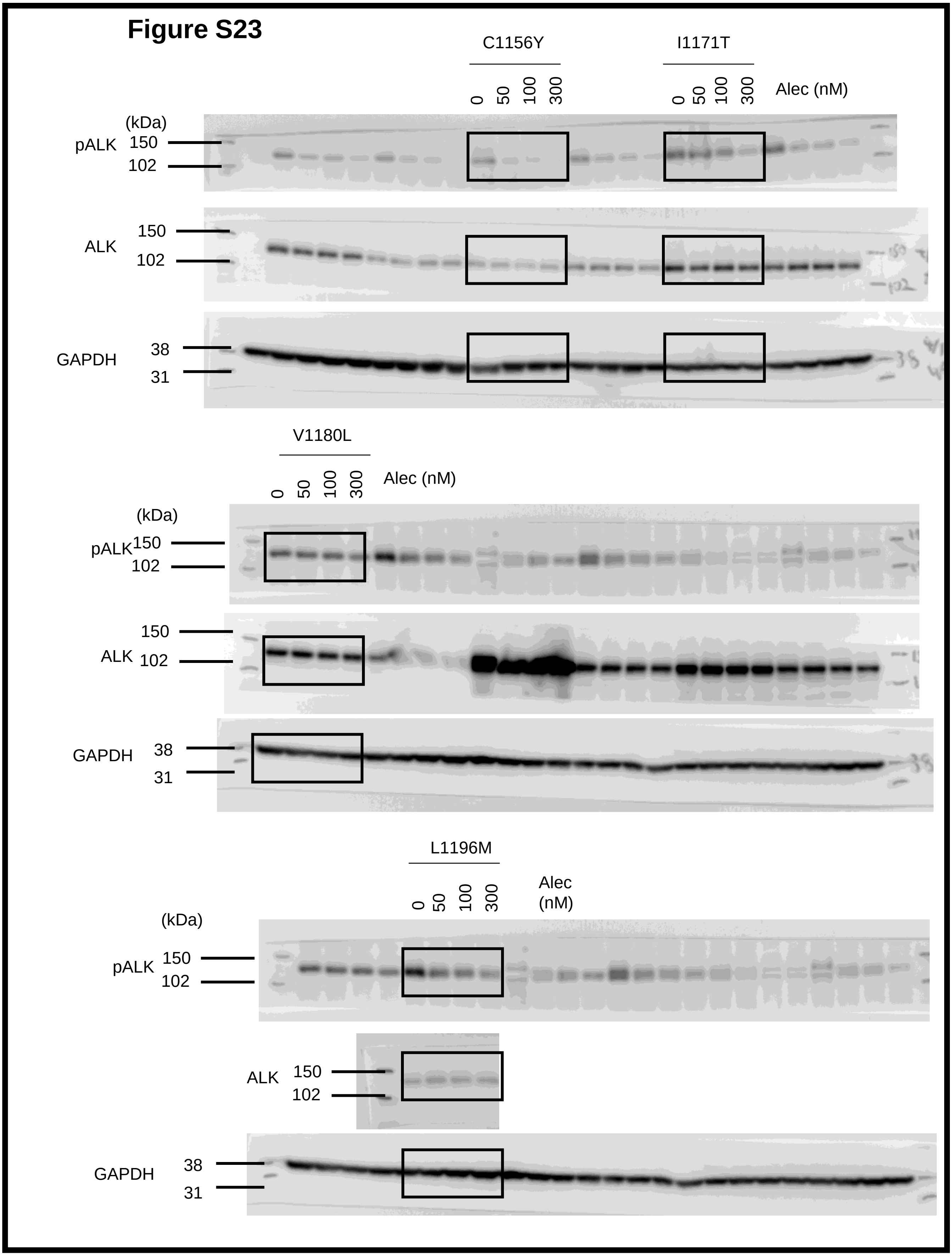

Supplementary Figure 28: Original immunoblots for indicated figures

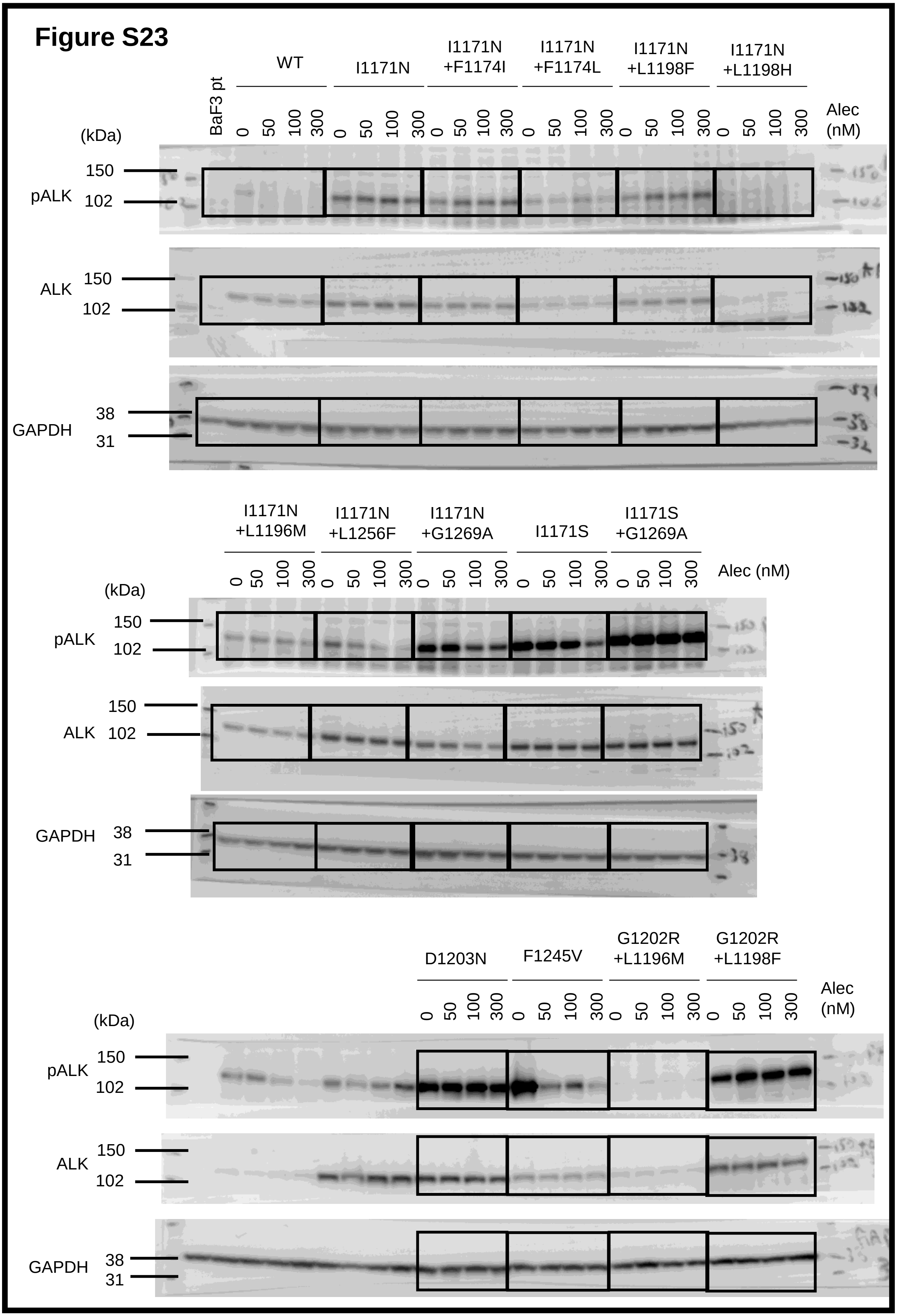

Supplementary Figure 28: Original immunoblots for indicated figures

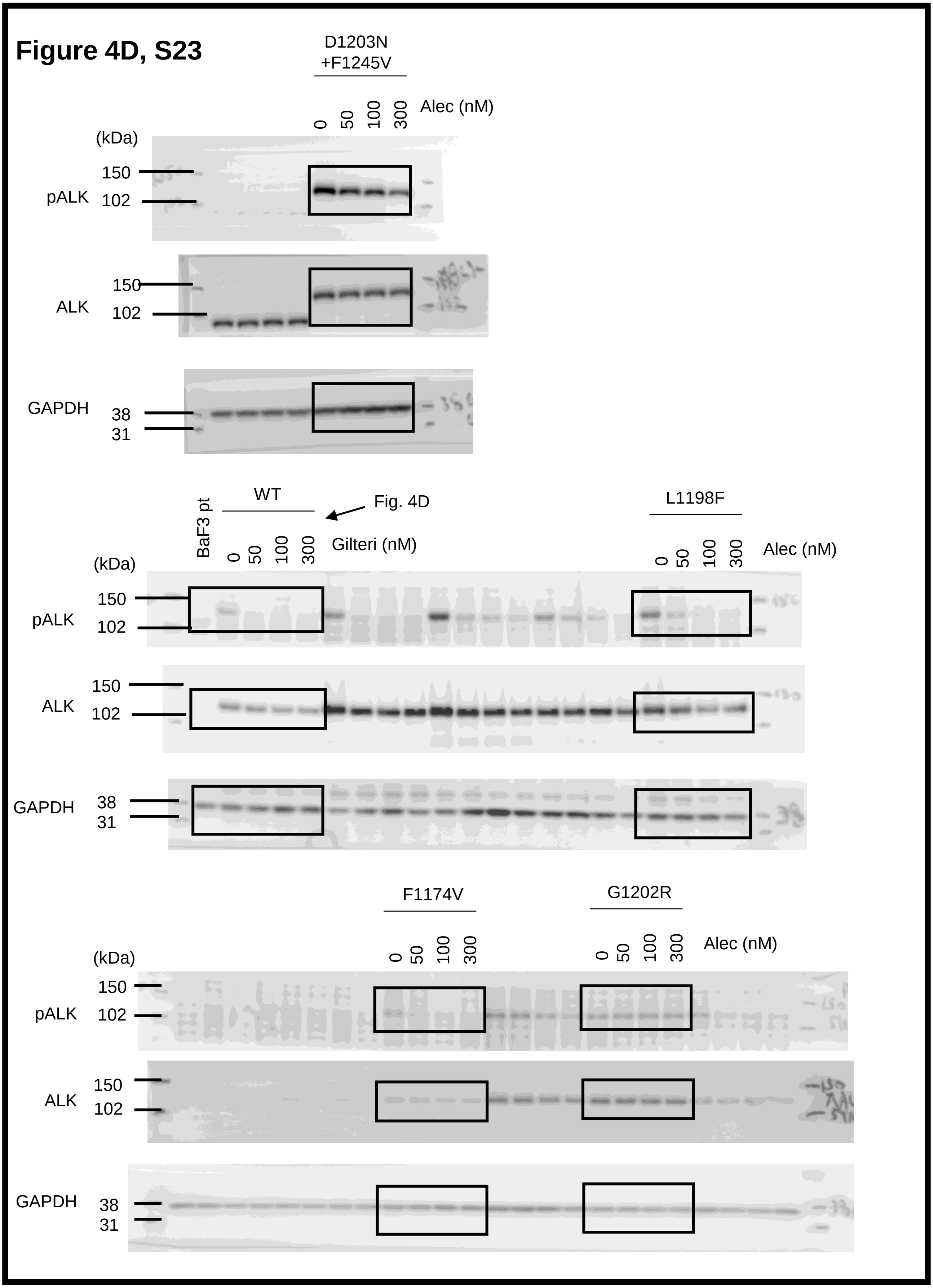

Supplementary Figure 28: Original immunoblots for indicated figures

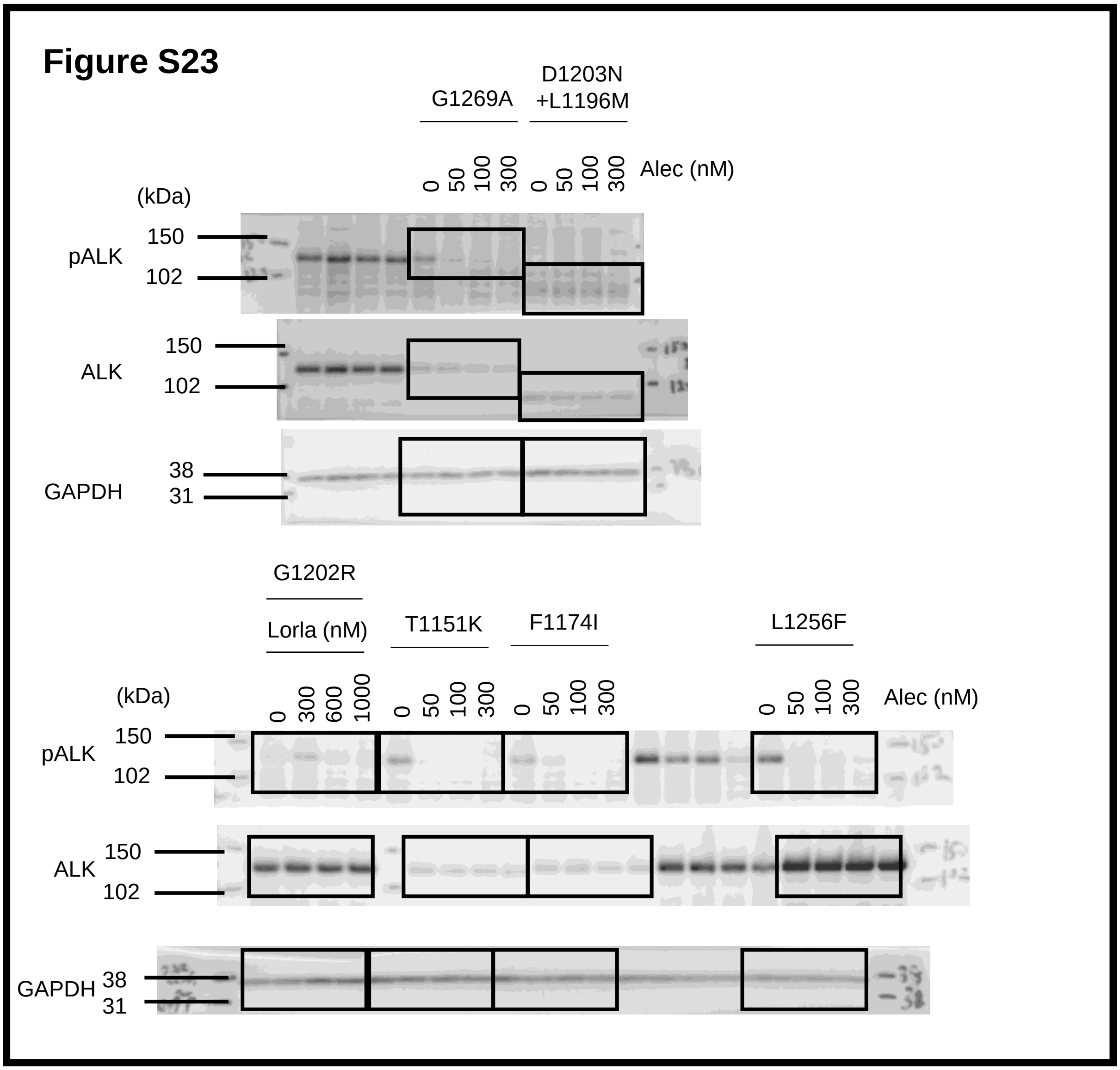

Supplementary Figure 28: Original immunoblots for indicated figures

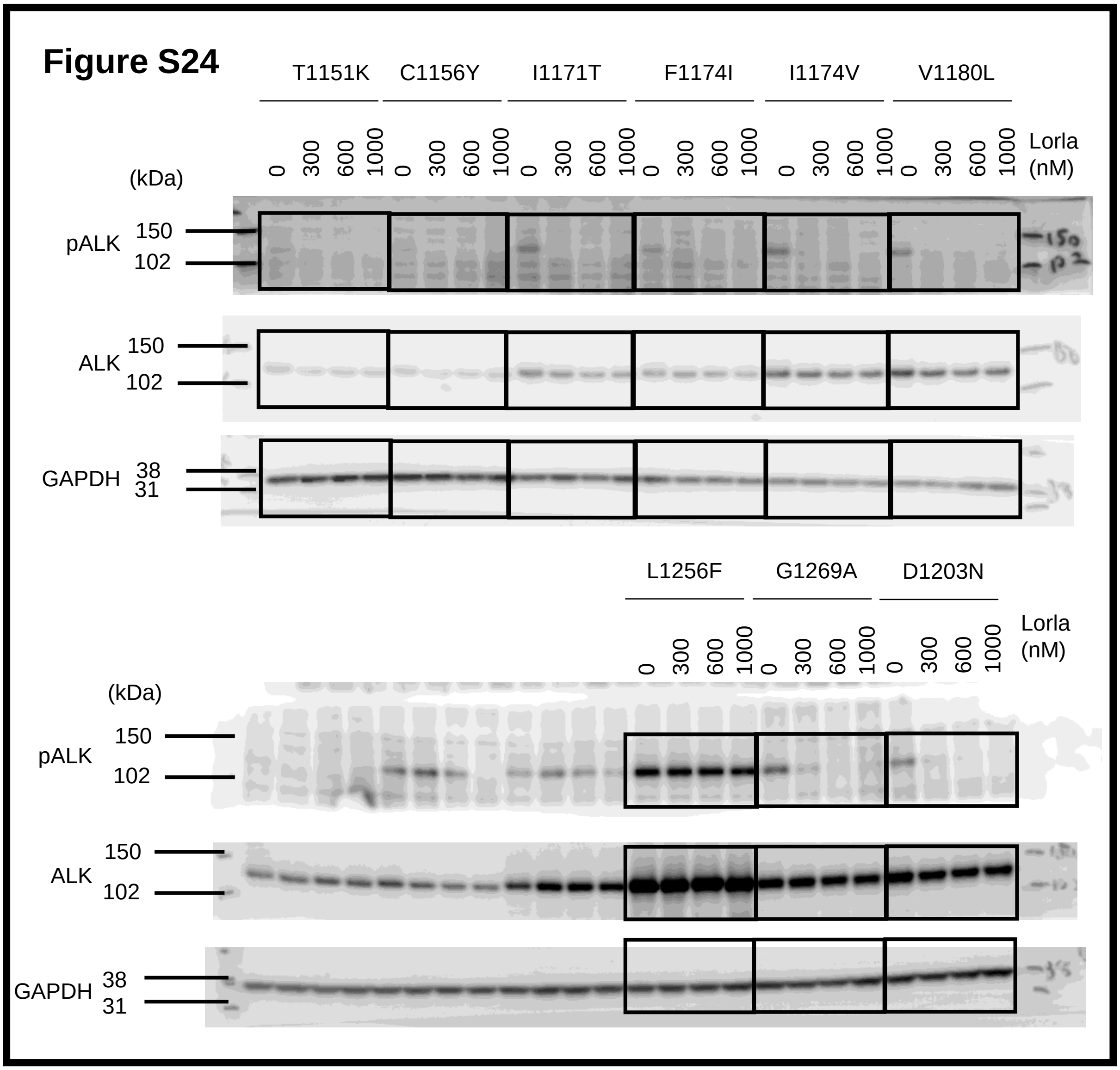

Supplementary Figure 28: Original immunoblots for indicated figures

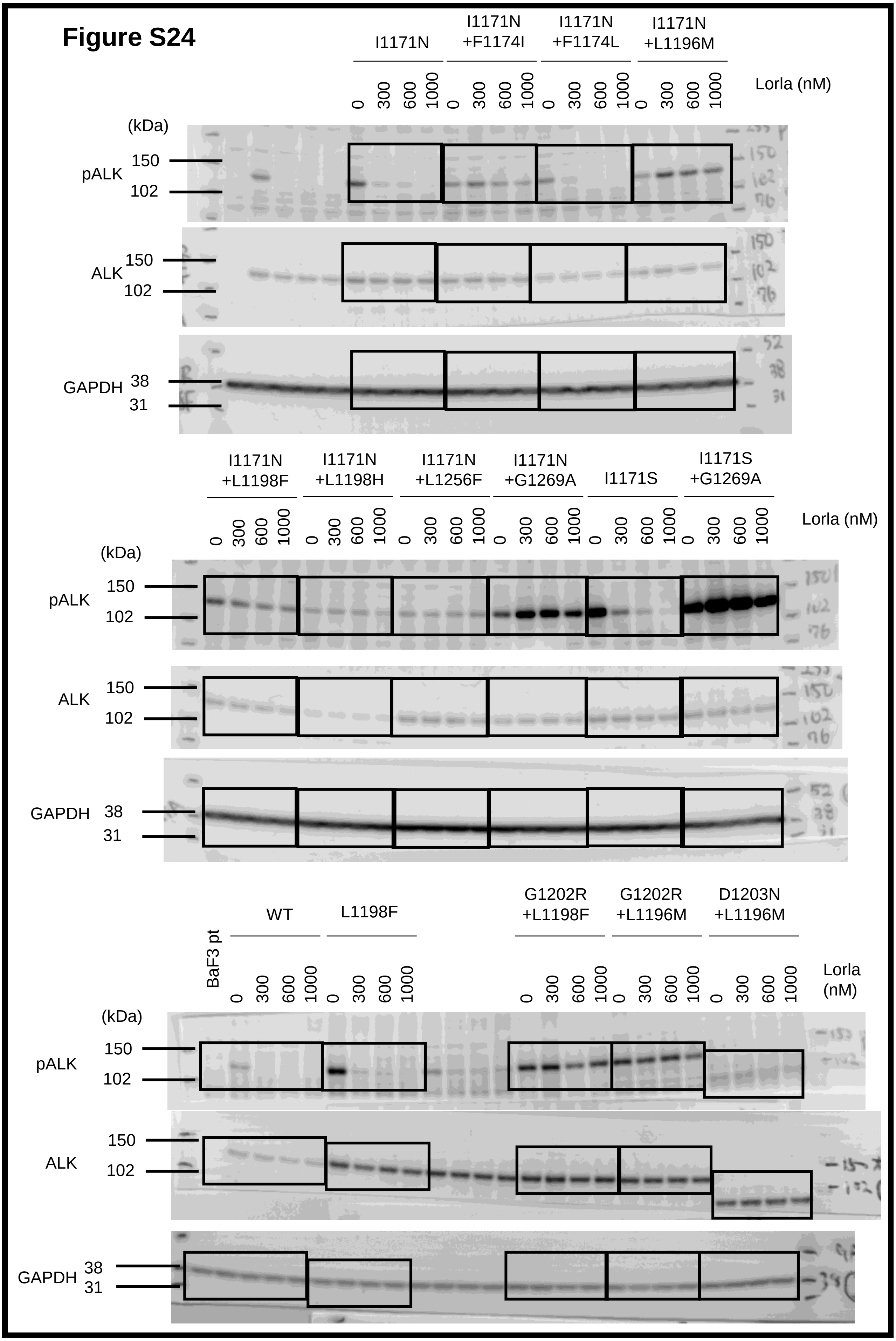

Supplementary Figure 28: Original immunoblots for indicated figures

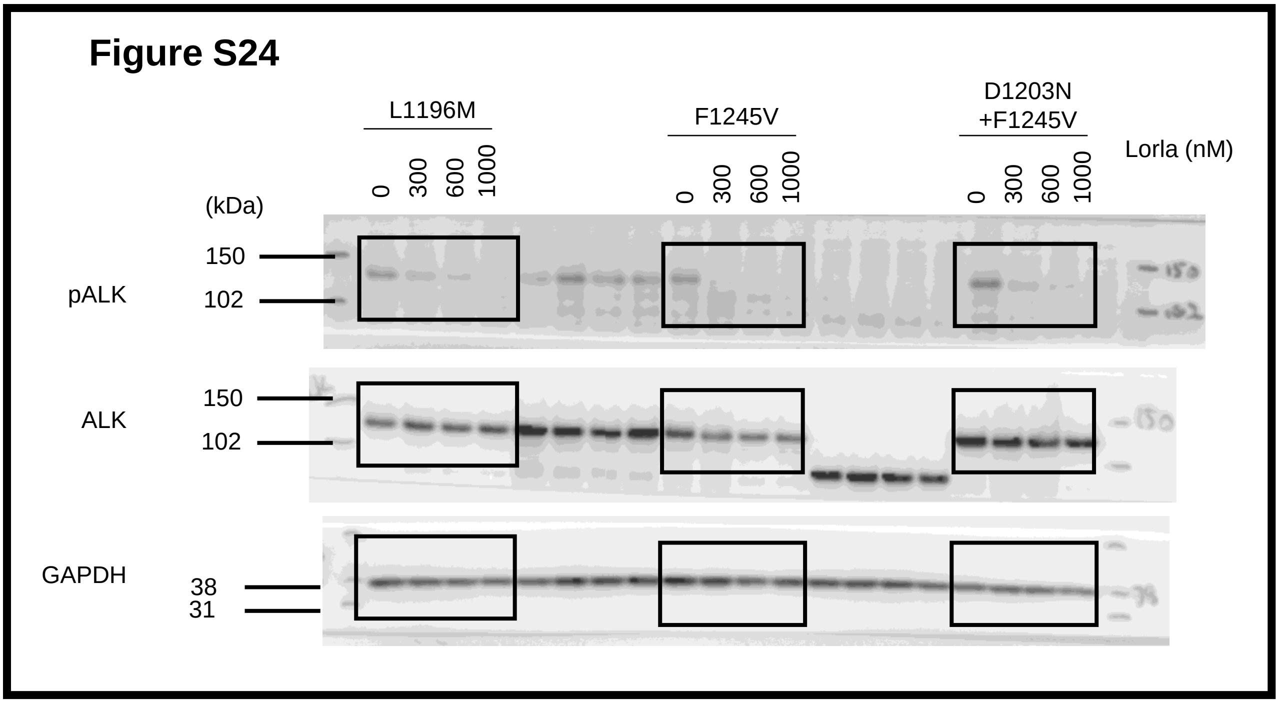

Supplementary Figure 28: Original immunoblots for indicated figures

Figure S26B

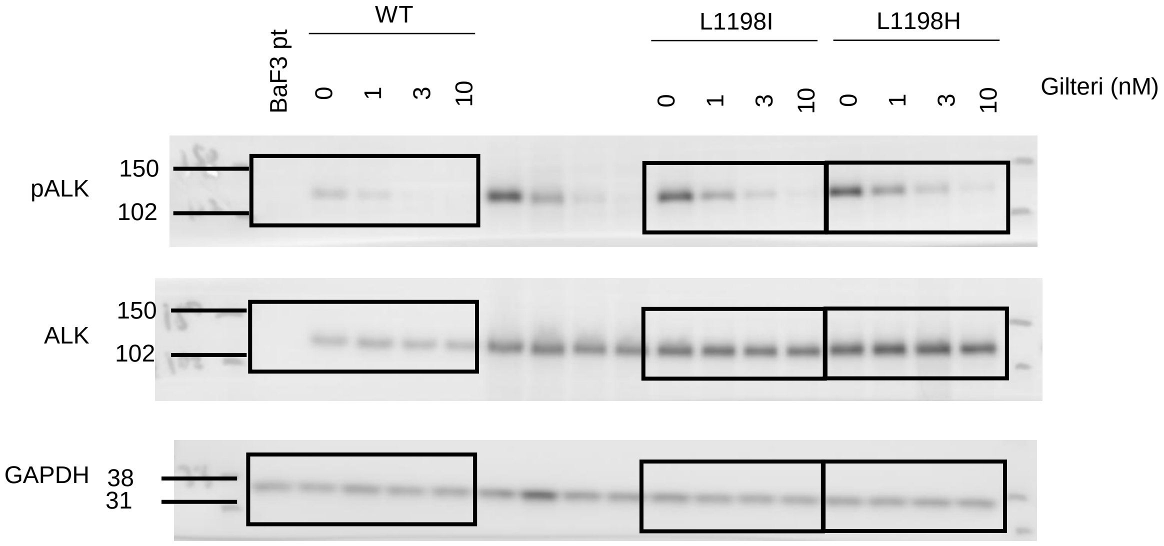

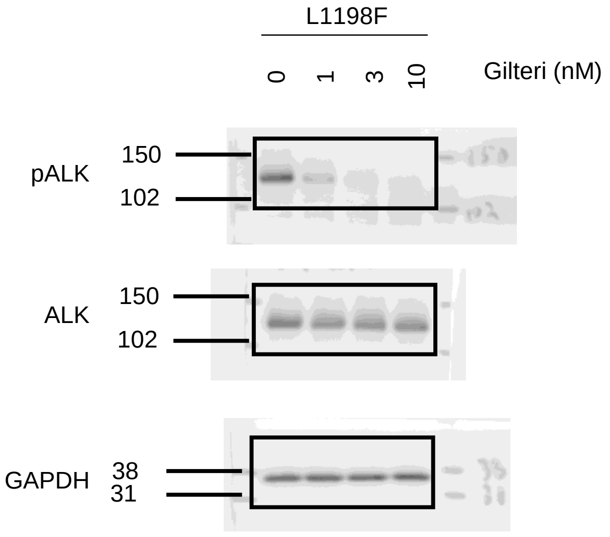

Supplementary Figure 29: Gating strategy for indicated figures

Figure 2C

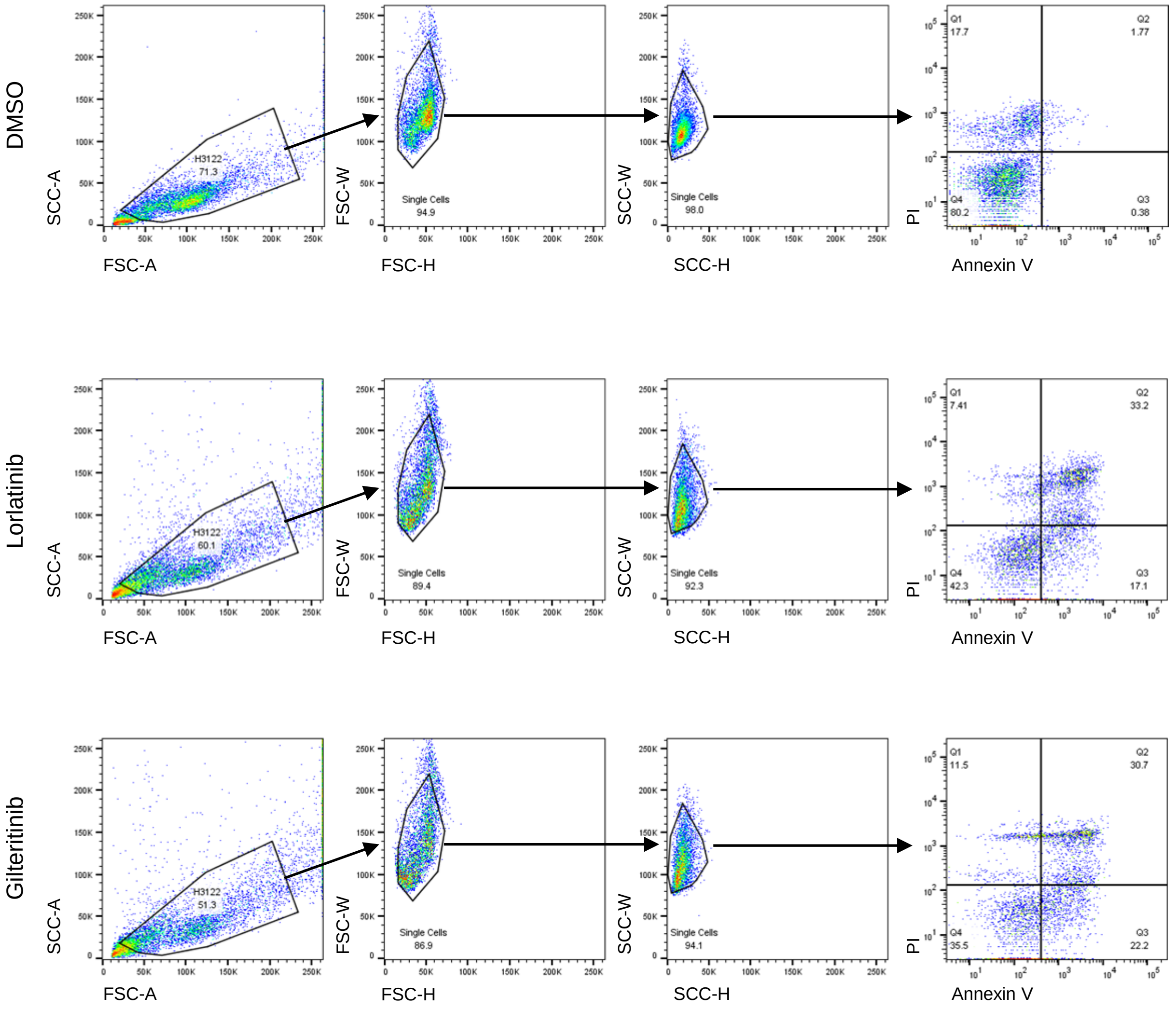

Supplementary Figure 29: Gating strategy for indicated figures

Figure 3C

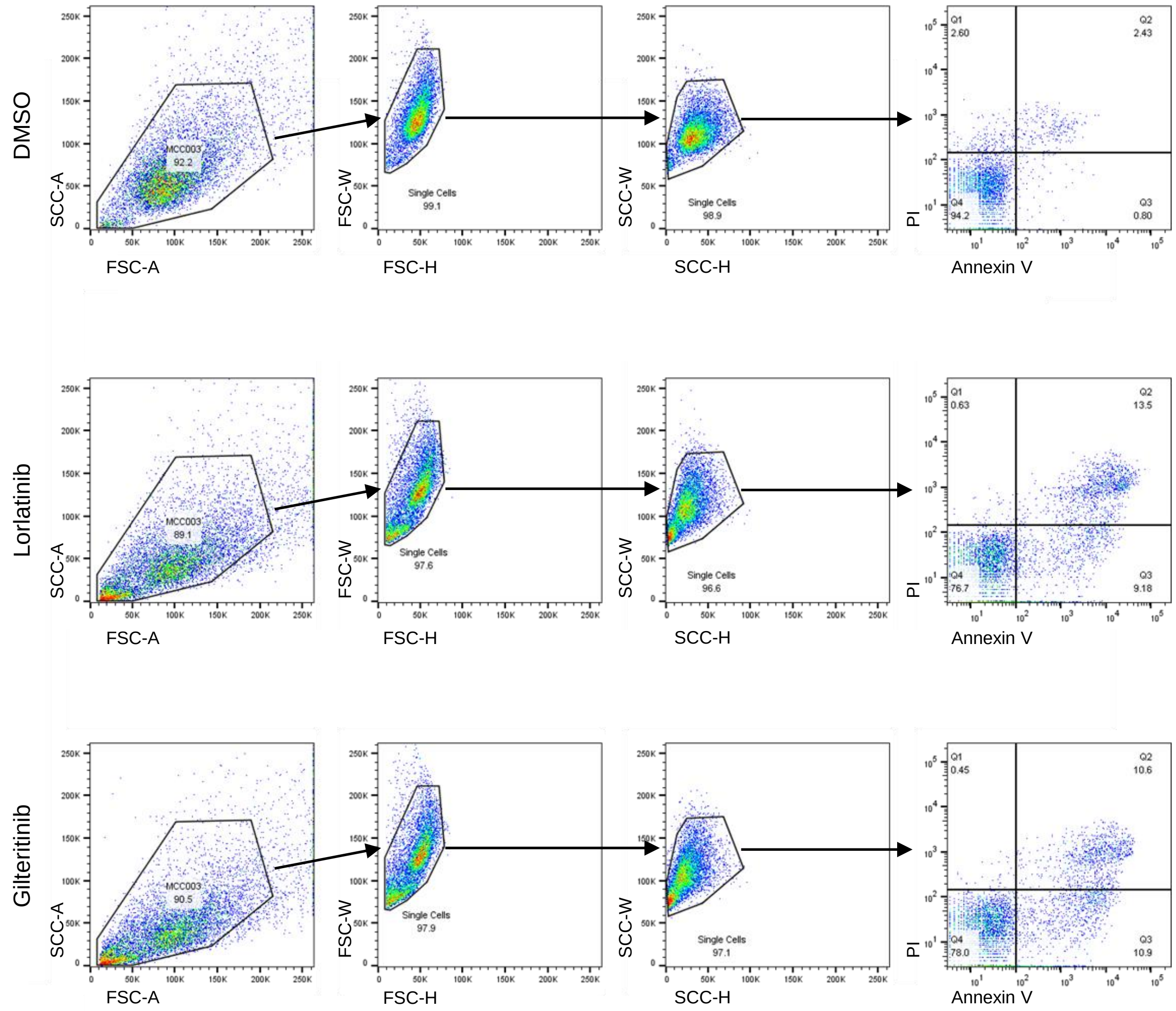

Supplementary Figure 29: Gating strategy for indicated figures

Figure S19D

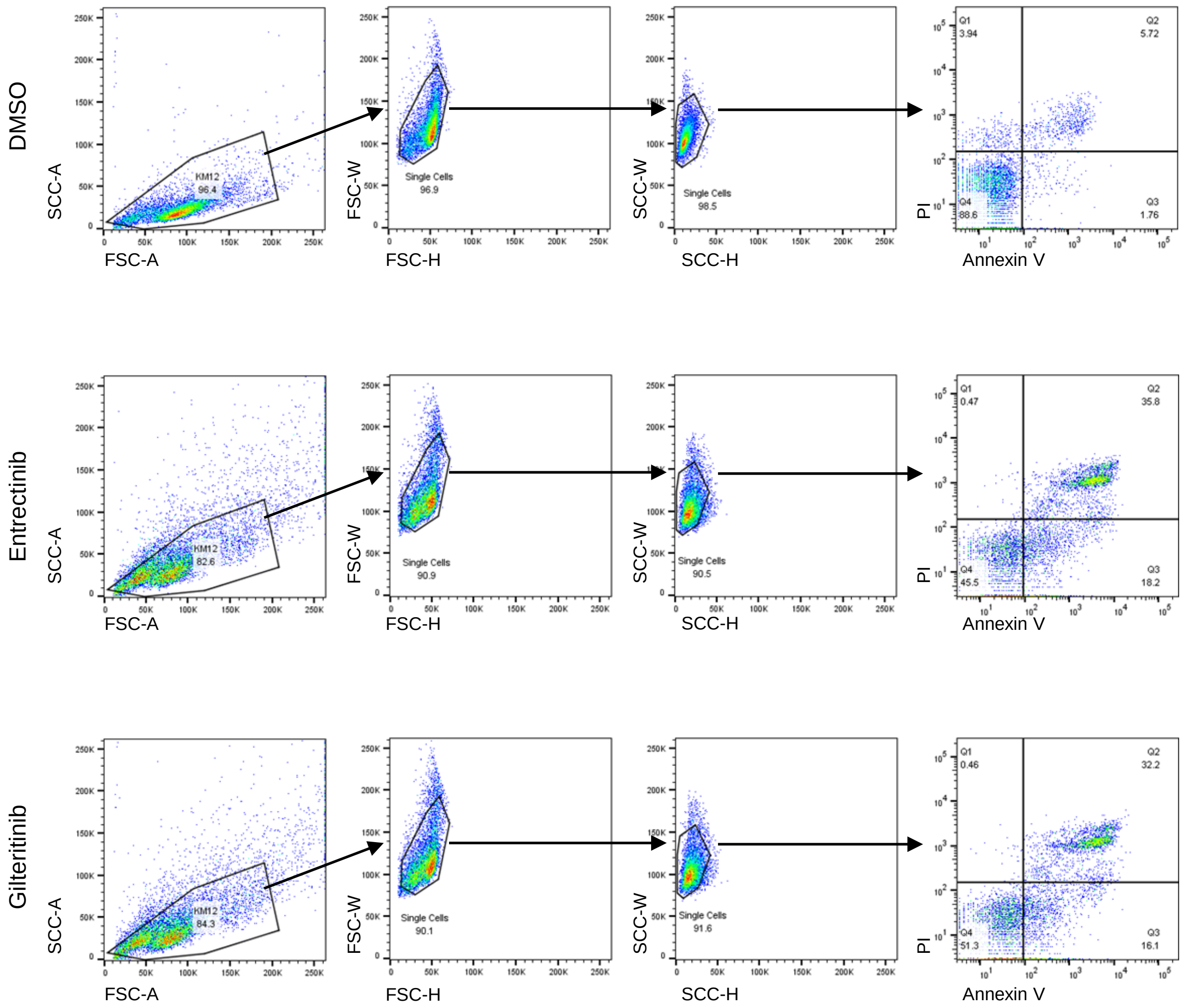

IC50 ≦ 50 nM	50 nM < IC50 ≦ 120 nM	120 nM < IC50 ≦ 300 nM	300 nM < IC50

IC50 ± SD ; nM	Crizotinib	Alectinib	Ceritinib	Brigatinib	Lorlatinib	Entrectinib	Gilteritinib
pt (+IL-3)	371 ± 50	899 ± 12	623 ± 33	578 ± 113	8238 ± 2411	481 ± 89	177 ± 4.9
WT	21 ± 7.5	7.3 ± 1.8	4.5 ± 1.6	2.8 ± 1.6	1.2 ± 0.38	19 ± 10	0.78 ± 0.27
T1151K	85 ± 80	16 ± 13	43 ± 50	4.6 ± 3.4	4.8 ± 3.1	19 ± 17	1.2 ± 0.93
C1156Y	71 ± 29	10 ± 3.6	20 ± 10	3.2 ± 1.2	4.4 ± 1.4	25 ± 10	0.66 ± 0.33
I1171N	189 ± 34	474 ± 96	27 ± 3.9	21 ± 5.7	87 ± 9.8	729 ± 86	6.1 ± 1.3
I1171T	122 ± 29	57 ± 12	14 ± 4.0	6.8 ± 0.59	21 ± 3.4	196 ± 60	4.2 ± 0.90
I1171S	65 ± 21	78 ± 21	11 ± 4.1	7.1 ± 1.7	38 ± 9.2	294 ± 89	2.9 ± 1.0
F1174I	92 ± 60	28 ± 16	30 ± 17	16 ± 10	10 ± 6.4	852 ± 630	4.7 ± 3.3
F1174V	68 ± 58	22 ± 17	20 ± 12	9.3 ± 8.1	6.2 ± 5.4	318 ± 223	3.4 ± 2.9
V1180L	32 ± 1.2	248 ± 25	2.2 ± 0.45	1.2 ± 0.32	2.3 ± 0.48	22 ± 4.5	1.4 ± 0.12
L1196M	249 ± 18	90 ± 19	21 ± 2.8	14 ± 1.0	73 ± 34	147 ± 46	20 ± 6.8
L1196Q	181 ± 39	336 ± 91	42 ± 18	21 ± 12	33 ± 8.5	405 ± 57	26 ± 4.5
L1198F	3.1 ± 0.80	36 ± 12	38 ± 8.3	15 ± 3.1	14 ± 1.8	3.2 ± 0.92	0.10 ± 0.02
G1202R	415 ± 61	838 ± 148	218 ± 32	69 ± 10	111 ± 8.0	5204 ± 2190	168 ± 37
D1203N	157 ± 96	28 ± 18	41 ± 18	20 ± 5.9	14 ± 5.1	168 ± 54	53 ± 17
F1245V	38 ± 9.1	14 ± 4.5	13 ± 0.41	5.0 ± 0.78	2.6 ± 0.62	127 ± 15	1.4 ± 0.26
L1256F	422 ± 115	2.6 ± 0.36	185 ± 25	16 ± 4.4	8801 ± 6443	2424 ± 1111	0.34 ± 0.07
G1269A	98 ± 21	18 ± 7.2	3.4 ± 0.63	1.1 ± 0.41	15 ± 3.8	59 ± 15	1.4 ± 0.19
I1171N+F1174I	343 ± 101	1188 ± 303	167 ± 20	120 ± 44	338 ± 41	5753 ± 2548	24 ± 4.4
I1171N+F1174L	73 ± 11	320 ± 124	32 ± 7.9	18 ± 8.0	37 ± 14	1112 ± 158	3.2 ± 0.24
I1171N+L1196M	299 ± 63	229 ± 110	12 ± 0.76	23 ± 10	355 ± 116	379 ± 169	14 ± 0.79
I1171N+L1198F	18 ± 2.8	860 ± 167	125 ± 27	59 ± 23	419 ± 99	60 ± 17	1.6 ± 0.16
I1171N+L1198H	100 ± 18	799 ± 163	218 ± 54	111 ± 28	358 ± 63	908 ± 132	6.9 ± 0.24
I1171N+L1256F	398 ± 30	37 ± 11	182 ± 26	18 ± 4.4	5012 ± 1857	3076 ± 572	0.41 ± 0.09
I1171N+G1269A	607 ± 49	880 ± 232	14 ± 2.8	6.4 ± 1.2	625 ± 85	2521 ± 419	11 ± 3.2
I1171S+G1269A	543 ± 75	617 ± 192	14 ± 1.7	5.1 ± 1.8	855 ± 90	2446 ± 346	13 ± 2.3
G1202R+L1196M	361 ± 37	834 ± 109	318 ± 81	190 ± 45	1050 ± 583	1711 ± 215	117 ± 4.1
G1202R+L1198F	35 ± 17	296 ± 80	299 ± 130	366 ± 152	317 ± 107	183 ± 78	32 ± 21
D1203N+L1196M	304 ± 15	202 ± 34	140 ± 13	101 ± 0.64	266 ± 52	278 ± 5.2	109 ± 21
D1203N+F1245V	170 ± 95	46 ± 31	87 ± 38	39 ± 2.1	30 ± 9.5	810 ± 263	64 ± 5.9

Supplementary Table 1. Summary of IC_{50} values in EML4-ALK mutations expressing Ba/F3 cells

Parental Ba/F3 cells and each EML4-ALK mutations expressing Ba/F3 cells were treated with indicated inhibitors for 72 h and the cell viability was assessed using the CellTiter-Glo assay. Results are expressed as mean ± SD.

	ALK positive NSCLC						ALK negative NSCLC			
	H3122		JFCR-028-3		MCC-003		A549		H460	
Rank	Phosphosite	Fold change	Phosphosite	Fold change	Phosphosite	Fold change	Phosphosite	Fold change	Phosphosite	Fold change
1	IRS2_Y576	-5.59	ALK_Y1584	-5.92	ALK_Y1586	-5.63	RPS6_S244	-2.18	NUDC_S139	-1.93
2	ALK_Y1586	-5.37	ALK_Y1586	-5.70	PTPRB_S293	-5.54	XIAP_T359	-2.09	NUDC_T145	-1.93
3	ALK_Y1604	-5.16	IRS1_Y87	-5.55	PTPRB_T287	-5.54	HNRNPA1_Y289	-2.06	NUDC_T145	-1.80
4	ALK_Y1586	-5.06	PTPRB_S293	-5.50	DLG5_Y78	-4.97	FLII_T913	-2.00	SULT2B1_S333	-1.70
5	EML4_Y265	-5.03	PTPRB_T287	-5.50	SPDEF_Y313	-4.41	HDGFRP2_S625	-1.98	SCAMP3_Y83	-1.24
6	RBM4B_Y37	-4.96	DLG5_Y78	-5.34	SCEL_Y58	-4.40	HDGFRP2_S613	-1.98	NUP153_S257	-1.19
7	SOS2_Y646	-4.77	IRS1_Y632	-5.24	ALK_Y1586	-4.04	SPAG9_S732	-1.89	ERCC6L_S1069	-1.15
8	ALK_Y1584	-4.50	ALK_Y1604	-4.88	PTPN11_Y546	-3.95	PRPF4B_S427	-1.85	CASKIN2_S303	-1.13
9	IRS1_Y87	-4.32	SHC3_Y256	-4.58	ALK_Y1604	-3.83	STX4_S14	-1.84	ATF2_S248	-1.12
10	ALK_Y1096	-4.28	RBM4B_Y37	-4.49	CBL_Y674	-3.83	STX4_S15	-1.84	MYO1E_Y971	-1.11
11	ALK_Y1092	-4.27	ALK_Y1586	-4.36	ALK_Y1584	-3.72	MSL1_S362	-1.83	TP53BP1_S398	-1.08
12	SPDEF_Y313	-4.25	INPP5D_Y915	-4.24	SH2B1_Y439	-3.60	PARVA_S8	-1.76	CNNM1_Y856	-1.04
13	RPS6_S235	-4.17	SOS2_Y646	-4.17	IRS1_Y87	-3.57	NUP153_S192	-1.75	AFAP1L2_S344	-1.03
14	RBM4B_Y113	-4.03	RBM4B_Y113	-4.04	STAT3_Y705	-3.44	NUCKS1_S73	-1.75	CAP1_S307	-1.02
15	ARHGEF5_Y641	-4.02	IRS2_Y576	-3.99	PEAK1_Y531	-3.43	NUCKS1_S75	-1.75	ARL6IP4_S225	-1.02
16	SHC1_Y410	-4.02	PELO_Y99	-3.86	IRS1_Y632	-3.28	NUCKS1_S79	-1.75	PARD3_S1123	-1.01
17	FRMD4B_Y816	-3.90	CRK_Y136	-3.74	RBM4B_Y113	-3.23	ZC3H18_T162	-1.59	MKI67_S827	-1.01
18	PTPRB_S293	-3.85	IRS2_Y111	-3.72	INPPL1_Y886	-3.14	CDCA5_S21	-1.59	MGA_S645	-1.00
19	PTPRB_T287	-3.85	IRS2_Y116	-3.72	RBM4B_Y37	-3.09	CCDC97_S264	-1.57		
20	IRS2_Y111	-3.73	EML4_Y265	-3.69	INPPL1_Y1135	-2.94	PSIP1_T122	-1.57		
21	IRS2_Y116	-3.73	RPS6_S244	-3.48	ARHGEF5_Y641	-2.92	CCDC132_S494	-1.52		
22	SCEL_Y58	-3.69	IRS2_Y116	-3.45	SHC3_Y256	-2.91	ILF3_S382	-1.47		
23	TNS4_Y644	-3.64	CBL_Y674	-3.36	PTPN11_Y584	-2.86	NTHL1_S56	-1.45		
24	SHC3_Y256	-3.63	IRS1_S636	-3.33	IRS2_Y576	-2.83	OTUD7B_S467	-1.45		
25	CRK_Y136	-3.57	SPDEF_Y313	-3.22	FRS2_Y306	-2.82	ABCF1_S166	-1.44		
26	DLG5_Y78	-3.43	HSPB1_Y133	-3.17	SOS2_Y646	-2.78	COL17A1_S148	-1.44		
27	ALK_Y1096	-3.40	PTPN11_Y546	-3.13	IRS2_S577	-2.74	MAP1A_S1600	-1.44		
28	GAB1_Y406	-3.31	ALK_Y1096	-3.09	RPS6_S244	-2.72	CTNNAL1_S454	-1.43		
29	ALK_Y1507	-3.27	GRB10_Y346	-3.07	TNS4_Y644	-2.71	SON_S1784	-1.43		
30	CBL_Y674	-3.22	ALK_Y1096	-3.05	IRS2_Y116	-2.66	RALGPS1_S298	-1.42		

Log_2 FC

-6 -1

Supplementary Table 2. Summary of downregulated phosphoproteins by gilteritinib treatment

Top 30 significantly downregulated phospho-sites ($p < 0.05$, Log_2(gilteritinib/DMSO) FC < -1) were identified in phosphoproteomics analysis. The average of each gilteritinib treated group (H3122, n=3, JFCR-028-3, n = 3, MCC-003, n= 3, A549, n=3, H460, n=2) were compared with the averages of each DMSO treated group.

A

IC50 ≦ 50 nM	50 nM < IC50 ≦ 120 nM	120 nM < IC50 ≦ 300 nM	300 nM < IC50

IC50±SD ; nM	Crizotinib	Alectinib	Ceritinib	Brigatinib	Lorlatinib	Entrectinib	Gilteritinib
H3122	60±0.19	23±6.4	15±2.1	7.9±3.7	1.6±0.23	44±12	1.6±0.10
JFCR-018-1	136±14	48±8.0	30±3.0	7.7±0.46	2.1±0.33	80±18	2.2±0.25
JFCR-028-3	49±8.1	23±11	12±3.3	6.2±1.5	1.6±0.21	44±17	1.6±0.04
H2228	28±14	3.8±1.2	10±3.6	3.5±1.0	0.46±0.20	19±4.1	0.52±0.14
MCC-003	269±10	765±97	74±22	63±11	18±3.9	496±147	22±7.8
KARPAS299	51±5.3	43±7.5	16±6.9	7.1±2.0	2.3±1.0	42±8.5	2.6±0.32

B

IC50±SD ; nM	Lorlatinib	Entrectinib	Gilteritinib
KM12	138±6.0	2.6±0.28	22±5.3
NTRK WT	133±15	2.0±0.22	13±1.3
G667C	>1000	44±3.6	13±0.32
G595R	>1000	420±24	262±28

Supplementary Table 3. Summary of IC_{50} values in ALK or NTRK1 positive cancer cells. (A) Various ALK positive cancer cells were treated with indicated inhibitors for 72 h and the cell viability was assessed using the CellTiter-Glo assay. Results are expressed as mean ± SD. (B) KM12 cells and each TPM3-NTRK1 mutations expressing Ba/F3 cells were treated with indicated inhibitors for 72 h and the cell viability was assessed using the CellTiter-Glo assay. Results are expressed as mean ± SD.
