# Supplementary material for: Gilteritinib overcomes lorlatinib resistance in ALK-rearranged cancer
Source: Nat Commun. 2021 Feb 24;12:1261. doi: 10.1038/s41467-021-21396-w (PMC7904790; doi:10.1038/s41467-021-21396-w)
Supplement: Supplementary file 3 — Reporting Summary [file 41467_2021_21396_MOESM3_ESM.pdf]

nature research

Corresponding author(s): Ryohei Katayama (NCOMMS-20-19430A)

Last updated by author(s): Jan 22, 2021

Reporting Summary

Nature Research wishes to improve the reproducibility of the work that we publish. This form provides structure for consistency and transparency in reporting. For further information on Nature Research policies, see our Editorial Policies and the Editorial Policy Checklist.

Statistics

For all statistical analyses, confirm that the following items are present in the figure legend, table legend, main text, or Methods section.

n/a	Confirmed	
☐	☒	The exact sample size (*n*) for each experimental group/condition, given as a discrete number and unit of measurement
☐	☒	A statement on whether measurements were taken from distinct samples or whether the same sample was measured repeatedly
☐	☒	The statistical test(s) used AND whether they are one- or two-sided<br>*Only common tests should be described solely by name; describe more complex techniques in the Methods section.*
☒	☐	A description of all covariates tested
☒	☐	A description of any assumptions or corrections, such as tests of normality and adjustment for multiple comparisons
☐	☒	A full description of the statistical parameters including central tendency (e.g. means) or other basic estimates (e.g. regression coefficient) AND variation (e.g. standard deviation) or associated estimates of uncertainty (e.g. confidence intervals)
☐	☒	For null hypothesis testing, the test statistic (e.g. *F*, *t*, *r*) with confidence intervals, effect sizes, degrees of freedom and *P* value noted<br>*Give P values as exact values whenever suitable.*
☒	☐	For Bayesian analysis, information on the choice of priors and Markov chain Monte Carlo settings
☒	☐	For hierarchical and complex designs, identification of the appropriate level for tests and full reporting of outcomes
☒	☐	Estimates of effect sizes (e.g. Cohen's *d*, Pearson's *r*), indicating how they were calculated

Our web collection on statistics for biologists contains articles on many of the points above.

Software and code

Policy information about availability of computer code

Data collection	No software was used
Data analysis	GraphPad Prism version 7.0.4, MaxQuant version 1.6.14.0, FlowJo version 10.6.2, GOLD version 5.5, MOE version 2016.8, AMBER Tools version12, GROMACS version4

For manuscripts utilizing custom algorithms or software that are central to the research but not yet described in published literature, software must be made available to editors and reviewers. We strongly encourage code deposition in a community repository (e.g. GitHub). See the Nature Research guidelines for submitting code & software for further information.

Data

Policy information about availability of data

All manuscripts must include a data availability statement. This statement should provide the following information, where applicable:

- Accession codes, unique identifiers, or web links for publicly available datasets
- A list of figures that have associated raw data
- A description of any restrictions on data availability

The phosphoproteomics data used in this manuscript have been deposited to the jPOSTrepo with the identifier JPST001063. All the other data supporting the findings of this study are available within the article and its Supplementary Information files and from the corresponding authors upon reasonable request.

Field-specific reporting

Please select the one below that is the best fit for your research. If you are not sure, read the appropriate sections before making your selection.

- [x] Life sciences
- [ ] Behavioural & social sciences
- [ ] Ecological, evolutionary & environmental sciences

For a reference copy of the document with all sections, see nature.com/documents/nr-reporting-summary-flat.pdf

Life sciences study design

All studies must disclose on these points even when the disclosure is negative.

Sample size	We decided the sample size to verify satisfactory interanimal reproduciblity in reference to the report by Hata AN., et al. (Nature Medicine 22, 262–269 (2016)).
Data exclusions	No data were excluded.
Replication	All data presented were obtained from three or two independent experiments with similar outcomes. (see Figure legends and Methods)
Randomization	For in vitro experiments, cells were seeded identically at the onset of the experiments and randomized into the various treatment groups prior to the beginning of treatment protocols. For mice experiments, we randomized the mice into the each treatment group based on the tumor size prior starting treatment.
Blinding	All experiments were not performed blind. Each experiment was designed with proper controls, and samples for comparison were collected and analyzed under the same conditions

Reporting for specific materials, systems and methods

We require information from authors about some types of materials, experimental systems and methods used in many studies. Here, indicate whether each material, system or method listed is relevant to your study. If you are not sure if a list item applies to your research, read the appropriate section before selecting a response.

Materials & experimental systems

n/a	Involved in the study	
☐	☒	Antibodies
☐	☒	Eukaryotic cell lines
☒	☐	Palaeontology and archaeology
☐	☒	Animals and other organisms
☒	☐	Human research participants
☒	☐	Clinical data
☒	☐	Dual use research of concern

Methods

n/a	Involved in the study	
☒	☐	ChIP-seq
☐	☒	Flow cytometry
☒	☐	MRI-based neuroimaging

Antibodies

Antibodies used

Primary antibodies for western blot:
anti-ALK Ab: Cell Signaling Technology, Cat# 3633, Lot: 9
anti-phospho-ALK Ab (Y1604): Cell Signaling Technology, Cat# 3Y1282/1283); Cell Signaling Technology, Cat# 9687, Lot: 1
anti-S6 ribosomal protein Ab: Cell Signaling Technology, Cat# 217, Lot: 10
anti-phospho-S6 ribosomal protein Ab: Cell Signaling Technology, Cat# 5364, Lot: 8
anti-p42/44 ERK/MAPK Ab: Cell Signaling Technology, Cat# 9102, Lot: 27
anti-phospho-p42/44 ERK/MAPK Ab: Cell Signaling Technology, Cat# 9101, Lot: 30
anti-AKT Ab: Cell Signaling Technology, Cat# 4691, Lot: 20
anti-phospho-AKT Ab: Cell Signaling Technology, Cat# 4060, Lot: 25
anti-EGFR Ab: Cell Signaling Technology, Cat# 4267, Lot: 17
anti-phospho-EGFR Ab: Abcam, Cat# ab5644, Lot: GR3333992-1
anti-MEK1/2 Ab: Cell Signaling Technology, Cat# 9122, Lot: 12
anti-phospho-MEK1/2 Ab: Cell Signaling Technology, Cat# 9121, Lot: 56
anti-PARP Ab: Cell Signaling Technology, Cat# 9542, Lot: 15
anti-AXL Ab: Cell Signaling Technology, Cat# 4566, Lot: 2
anti-phospho-AXL Ab: Cell Signaling Technology, Cat# 5724, Lot:1
anti-KRAS Ab: Sigma-Aldrich, Cat# WH0003845M1, Lot: J5281-S2
anti-NTRK1 Ab: Cell Signaling Technology, Cat# 4609, Lot: 3
anti-phospho-NTRK1 Ab: Cell Signaling Technology, Cat# 4621, Lot: 3

anti-STAT3 Ab: Cell Signaling Technology, Cat# 4904, Lot: 7
anti-phospho-STAT3 Ab: Cell Signaling Technology, Cat# 9145, Lot: 22
anti-GAPDH Ab: Millipore, Cat# MAB374, Lot: 3527693

Secondary antibodies for western blot:
anti-rabbit IgG Ab: Sigma-Aldrich, Cat# NA934V, Lot: 17197685
anti-mouse IgG Ab: Sigma-Aldrich, Cat# NA931V, Lot: 17061154

Validation

All antibodies were obtained commercially and had been validated by the companies.

Eukaryotic cell lines

Policy information about cell lines

Cell line source(s)

293FT cell line (purchased from Thermo Fischer Scientific)
Ba/F3 cell line (purchased from Riken BRC cell bank)
NIH3T3 cell line (kindly gifted by Dr Tsuruo T)
A431 cell line (kindly gifted by Dr Tsuruo T)
H2228 cell line (purchased from ATCC)
H3122 cell line (kindly gifted by Dr. Engelman JA)
KM12 cell line (obtained from NCI)
HCC827 cell line (purchased from ATCC)
PC9 cell line (kindly gifted by Dr. Nishio K)
A549 cell line (obtained from NCI)
H460 cell line (obtained from NCI)
HCC78 cell line (purchased from DSMZ)
KARPAS299 cell line (purchased from ECACC)
TIG-3 cell line (kindly gifted by Dr. Kaji K)
JFCR-018-1 cells were established from EML4-ALK positive NSCLC patient
JFCR-028-3 cells were established from EML4-ALK positive NSCLC patient
MCC-003 cells were established from EML4-ALK positive NSCLC patient
JFCR-168 cells were established from CD74-ROS1 positive NSCLC patient
JFCR-256-3 cells were established from BRAF V600E positive NSCLC patient
MR347 cells were established from EML4-ALK positive NSCLC patient
JFCR-098 cells were established from EML4-ALK positive NSCLC patient

Authentication

Patient derived cell lines were confirmed by the sequencing of driver oncogenes. Other cells were obtained from public bioresources bank or company with the information of authentication, and gifted cells were examined by STR analysis before making the cell stock.

Mycoplasma contamination

All public cell lines were not detected mycoplasma by the PCR based assay kit. Patient derived cells were not tested for mycoplasma contamination.

Commonly misidentified lines
(See ICLAC register)

In this study, we have not used cell lines included in the commonly misidentified.

Animals and other organisms

Policy information about studies involving animals; ARRIVE guidelines recommended for reporting animal research

Laboratory animals

Balb-c/nu, females, 6 weeks of age .
SCID-beige, females, 6 weeks of age .

Wild animals

The study did not involve wild animals.

Field-collected samples

The study did not involve samples collected from the field.

Ethics oversight

All mice studies were conducted in line with the protocols approved by the Committee for the Use and Care of experimental animals of the Japanese Foundation for Cancer Research

Note that full information on the approval of the study protocol must also be provided in the manuscript.

Flow Cytometry

Plots

Confirm that:

- [x] The axis labels state the marker and fluorochrome used (e.g. CD4-FITC).
- [x] The axis scales are clearly visible. Include numbers along axes only for bottom left plot of group (a 'group' is an analysis of identical markers).
- [x] All plots are contour plots with outliers or pseudocolor plots.
- [x] A numerical value for number of cells or percentage (with statistics) is provided.

Methodology

Sample preparation	H3122, KM12, or MCC-003 cells (1.0×10^5) were seeded into 6-well plates and cultured in appropriate medium. After 24 h, cells were cultured in drug-containing medium (100 nM) for an additional 72 h. All floating and adherent cells were collected and stained with Alexa Fluor 647-labeled Annexin-V and propidium iodide using a Dead Cell Apoptosis Kit (Thermo Fischer Scientific) for 15min at room temperature in the dark. Each sample was evaluated using FACS Verse
Instrument	FACS Verse
Software	FlowJo
Cell population abundance	Over 10000 cells were counted for the apoptosis assay
Gating strategy	Gating strategy for AnnexinV-PI flow cytometry analysis was only with FSC and SSC

- [x] Tick this box to confirm that a figure exemplifying the gating strategy is provided in the Supplementary Information.
